# Extensive mutational ctDNA profiles reflect High-grade serous cancer tumors and reveal emerging mutations at recurrence

**DOI:** 10.1016/j.tranon.2023.101814

**Published:** 2023-11-02

**Authors:** Giovanni Marchi, Anna Rajavuori, Mai T.N. Nguyen, Kaisa Huhtinen, Sinikka Oksa, Sakari Hietanen, Sampsa Hautaniemi, Johanna Hynninen, Jaana Oikkonen

**Affiliations:** a Research Program in Systems Oncology, Faculty of Medicine, University of Helsinki, Helsinki 00291, Finland; b Department of Obstetrics and Gynecology, University of Turku and Turku University Hospital, 20521 Turku, Finland; c Satasairaala Central Hospital, Department of Obstetrics and Gynecology, 28500 Pori, Finland

**Keywords:** ctDNA, High-grade serous ovarian carcinoma, Liquid biopsy, Targeted sequencing, DNA mutations

## Abstract

**Objective:**

Circulating tumor DNA (ctDNA) offers a minimally-invasive alternative to study genomic changes in recurrent malignancies. With a high recurrence rate, the overall survival in high-grade serous ovarian carcinoma (HGSC) has remained low. Our objectives were to determine whether ctDNA from plasma adequately represents HGSC, and to find mutational changes at relapse suggesting therapy options that could alter patient outcome.

**Methods:**

We collected 152 longitudinal plasma and 92 fresh tissue samples from 29 HGSC patients, sequencing and detecting mutations with a gene panel of more than 700 cancer-related genes. Tumor content was measured using *TP53* VAF. We analyzed the concordance between the mutations in tissue and plasma samples and calculated correlations to patient outcomes. We also searched for novel mutations appearing at relapse.

**Results:**

The concordance rate between mutations in plasma compared to tumor tissue was 83 % at diagnosis and 90 % at relapse. CtDNA was released similarly from the tubo-ovarian tumors, intra-abdominal metastases and ascites. CtDNA release was high when CA-125 level was elevated. The *TP53* VAF in ctDNA from plasma samples before the third cycle of primary chemotherapy showed a negative correlation to patient outcome. At relapse, 19 novel, pathogenic DNA mutations appeared, suggesting possible actionable alterations and biological mechanisms related to chemoresistance.

**Conclusion:**

Relapse ctDNA samples reflect tissue samples well and longitudinal sampling provides a timely source for mutational profiling. The emerging genetic mutations at recurrence propose that ctDNA accurately represents the widespread disease and provides possibilities for personalized therapy options.

## Highlights

• Mutations called from ctDNA are highly concordant with HGSC tumor tissue.
• Different anatomical locations release ctDNA similarly.
• Longitudinal ctDNA samples reveal targetable alterations appearing at relapse.

## Introduction

Recurrence affects 80 % of women with high grade serous ovarian cancer (HGSC) [1], making it the most lethal of gynecological malignancies in the Western world [2]. The high relapse rate is due to insufficient screening methods and late diagnosis [3], with 75 % diagnosed at a late stage IIIC or more [4]. Remarkably, if diagnosed early, the 5-year survival rate exceeds 90 % compared to 29 % for those diagnosed at stage IV [5]. Primarily, almost all patients undergo surgery, and diagnosis is histopathologically confirmed from tumor samples [6]. At relapse, however, sampling is often limited to ascites puncture, since secondary cytoreduction is limited to a few optimally chosen, platinum-sensitive patients [7,8].

Circulating tumor DNA (ctDNA) is the cancer-derived fraction of the extracellular DNA fragments present in the bloodstream [9]. Hence, ctDNA detection through liquid biopsy offers a minimally-invasive and widely accessible alternative to direct tumor sampling [10]. It has been shown that the ctDNA amount correlates to tumor burden, peaking at diagnosis and relapse [11]. Additionally, ctDNA has been explored as a potential biomarker of HGSC, demonstrating higher sensitivity and specificity than the currently most widely used clinical marker, CA-125 [12]. The first-line treatment of HGSC involves platinum-based chemotherapy and cytoreductive surgery [13]. While initial responses are often favorable, tumor heterogeneity and evolution allow the relapsed tumor to develop chemoresistance, which is ultimately fatal [14,15]. Consequently, examining the recurrent tumor is crucial for understanding the evolving cancer, especially when standard care proves ineffective. In such scenarios, ctDNA provides an opportunity to profile patient-specific gene mutations for individualistic therapy options [16,17]. However, before implementing ctDNA into clinical practice, it is essential to determine its efficacy in accurately representing HGSC tumors [10].

In this study, we analyzed a longitudinal ctDNA dataset composed of 152 plasma samples from 29 HGSC patients, sequenced with a panel of more than 700 cancer-related genes. To our knowledge, this is the largest published cohort on ctDNA in HGSC in terms of samples and genes. We demonstrate that plasma samples show a high concordance to fresh tissue regardless of tumor site and that they better represent tumor heterogeneity and evolution at relapse.

## Methods

### Patient enrolment and sample selection

**Fig. 1:**
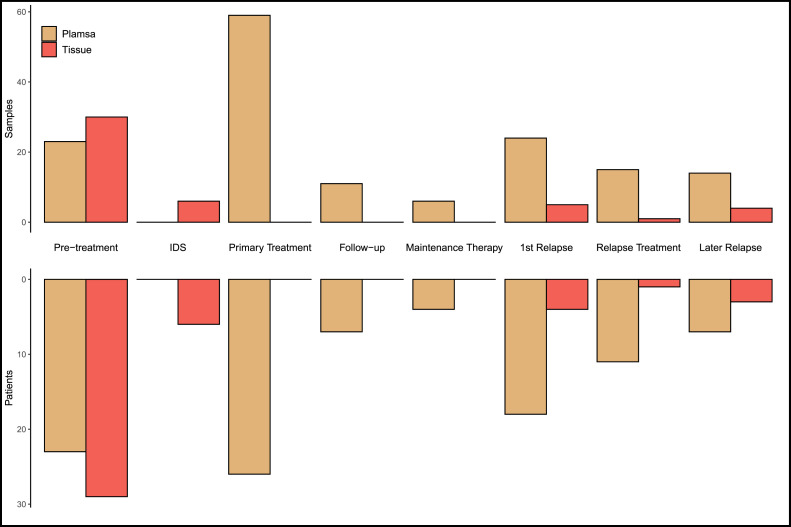
Distribution of samples and patients during the longitudinal sampling. “1st Relapse” refers to samples taken at the first disease recurrence, before the start of relapse treatment. “Relapse Treatment” includes samples taken during any line of chemotherapy following the 1st relapse. “Later Relapse” samples were taken when later recurrences were detected. IDS - interval debulking surgery after neoadjuvant chemotherapy.

We longitudinally collected plasma (*n* = 152) and tissue (*n* = 92) samples from 29 unselected patients participating in the on-going prospective study DECIDER [18] (Fig. 1). Of these, samples from 12 patients were also analyzed in a previously published study by our group [17]. All patients signed informed consent and the study had the permission of the Ethics committee of the Hospital District of Southwest Finland. Clinical features and CA-125 levels were gathered from medical records.

All patients were histologically diagnosed with HGSC and were treated according to standard care. The pre-treatment tissue samples were gathered from primary debulking surgery or laparoscopy for patients allocated to neoadjuvant chemotherapy (NACT). Additionally, samples were gathered during primary treatment from interval debulking surgery (IDS) after NACT. The fresh tissue samples were either from the ovaries and fallopian tubes (subsequently referred to as adnexa) or other intra-abdominal regions such as the omentum, peritoneum and bowel mesentery. The fresh samples at relapse were tumor cells extracted from ascites from six patients that needed an ascites puncture to relieve their symptoms. None of the patients underwent surgery at relapse. Both fresh tissue and ascites samples will be referred to as tissue samples from here onwards. The pre-treatment plasma samples were taken before initial surgery while the other plasma samples were collected during chemotherapy and clinical follow-up visits. No patient was burdened by unnecessary sampling.

### Sample collection and DNA sequencing

A volume of 5–6 ml whole blood was collected into EDTA-coated tubes, followed by mechanical agitation and centrifugation (repeated twice, 2000 x *g* for 10 min). The processed plasma samples were then aliquoted (1 to 2 mL), stored at −80 °C within 2 h and finally used to extract cell free DNA (cfDNA). In addition to cfDNA, genomic DNA was isolated from liquid nitrogen snap-frozen tumor tissue, ascites and whole blood buffy coat samples. The whole blood buffy coat samples were used as germline controls.

Both cfDNA and genomic DNA were sequenced at BGI Europe A/S (Copenhagen, Denmark) with the Oseq™ panel. Sequencing was performed on plasma samples with 1000x coverage, and on genomic DNA with 200x coverage. Due to upgrades in the Oseq™ panel, two targets of 427 and 584 protein-coding cancer-related genes, amounting to 722 unique ones (Supplementary Table S1), were used to sequence two corresponding groups of DNA samples composed of 100 plasma, 29 tissue and 13 normal blood samples and 52 plasma, 17 tissue and 16 normal control samples, respectively.

### Data processing

DNA samples were analyzed through bioinformatic processing, including quality control, alignment to reference human genome, deduplication, cross-sample contamination estimation and variant discovery. A comprehensive description of this entire pipeline is provided in the Supplementary text. Briefly, somatic mutation detection was performed using Somatic Alterations in Genome (SAGE) tool [19] in a paired tumor-normal mode with default parameters. A series of custom filters were applied to the called mutations to remove false positives increasing specificity. The whole data processing step was performed in the Anduril2 workflow platform [20].

### Plasma-tissue concordance evaluation

To evaluate the alignment between ctDNA and tissue samples, we computed the concordance with two different equations. First, for tissue-based concordance, we determined the ratio of shared mutations in both plasma (p) and tissue samples to mutations found in tissue (t) ($Ct=\frac{Mp\cap Mt}{Mt}$). Second, for plasma-based concordance, shared mutations were compared to those identified in plasma ($Cp=\frac{Mp\cap Mt}{Mp}$). Concordance was analyzed for pre-treatment (*n* = 24) and relapse (*n* = 9) plasma-tissue pairs, with the samples in each pair belonging to the same patient and collected within a three-day window.

Furthermore, when assessing tissue-site concordance, samples from the adnexa (*n* = 11), ascites (*n* = 13) and other intra-abdominal sites (*n* = 21) were compared to corresponding plasma samples.

Notably, plasma samples that had a tumor content beneath the detection threshold (two from pre-treatment and four from relapse) were still factored into our concordance evaluations.

### Clinical correlation

Platinum-free interval (PFI) is defined as the time to progression from the end of primary therapy and overall survival (OS) as the time from diagnosis to death. First progression was determined clinically either by a two-fold increase in the CA-125 level or radiologically [21]. We correlated the *TP53* variant allele frequency (VAF) of plasma samples taken before treatment (*n* = 23) and during primary therapy before the third cycle (*n* = 12) to PFI and OS. We also explored the relationship between *TP53* VAF and CA-125 levels, using paired samples gathered within a three-day window.

### Detection of novel mutations at relapse

Functional mutations emerging at relapse were prioritized by estimating pathogenicity (CADD [22] score >10) of events detected from relapse samples and absent in pre-relapse samples. Over-representation analysis was conducted using the Kyoto Encyclopedia of Genes and Genomes (KEGG) database [23] to identify biological pathways enriched in genes involved in the relapse-emerging events. Pathway analysis was performed using the ConsensusPathDB [24] algorithm, providing the list of genes sequenced in the Oseq panel as background gene list. We also screened for the presence of drug targets by querying the list of events emerging at relapse into the Molecular Tumor Board database [25].

### Statistical analyses

We conducted statistical analyses using R software (version 4.3.1). Spearman's rank correlation method was employed for correlation analyses. A *p*-value threshold of 0.05 was set to determine significant statistical comparisons.

## Results

### Patients and samples

The 29 patients enrolled in our study (Table 1 and Supplementary Table S2) had a median age of 68. All except two had debulking surgery either as primary debulking surgery (PDS) or after neoadjuvant chemotherapy (NACT). Most of the patients had 5–10 cycles of platinum-based chemotherapy as primary treatment, typically a combination of carboplatin and paclitaxel. The two patients with only 2–3 cycles of primary chemotherapy progressed during the treatment, leading to a change in chemotherapy regimen. Half of the patients had a PFI under six months, i.e. were platinum-resistant. All 29 patients had a pre-treatment tumor sample, and 23 patients had a pre-treatment plasma sample (Fig. 1). One patient had an explorative laparoscopy and five patients an ascites puncture before pre-treatment tumor sampling, which could potentially influence ctDNA levels [10].

**Table 1:** Clinical features of the 29 HGSC patients.

	Platinum resistant (14)	Platinum sensitive (15)
Age (range)	59 – 81	57 – 80
**Treatment strategy** PDS NACT	3 11	11 4
**FIGO stage** IIB IIIC IVA IVB	0 7 4 3	1 12 1 1
**Primary therapy outcome** Complete response Partial response Stable disease Progressive disease	6 4 0 4	8 7 0 0
**Residual tumor at debulking surgery** 0 1–10 mm More than 10 mm No debulking	5 4 2 3	5 7 3 0
**Primary chemotherapy** Paclitaxel-carboplatin Carboplatin only Bevacizumab included	10 4 2	13 2 3
1st relapse (PFI range)	14 (2 – 76 days)	13 (195 – 1499 days)
**2nd line chemo** Platinum-based Paclitaxel weekly Other No further treatment	0 9 4 0	9 0 3 3
**3rd-5th line chemo** Platinum-based Paclitaxel weekly Other	*n* = 19 6 0 15	*n* = 17 6 3 8
Overall survival (range) Alive	348 – 1295 days 0	425–2167 days 4

### Tumor content in ctDNA correlates with CA-125 level

**Fig. 2:**
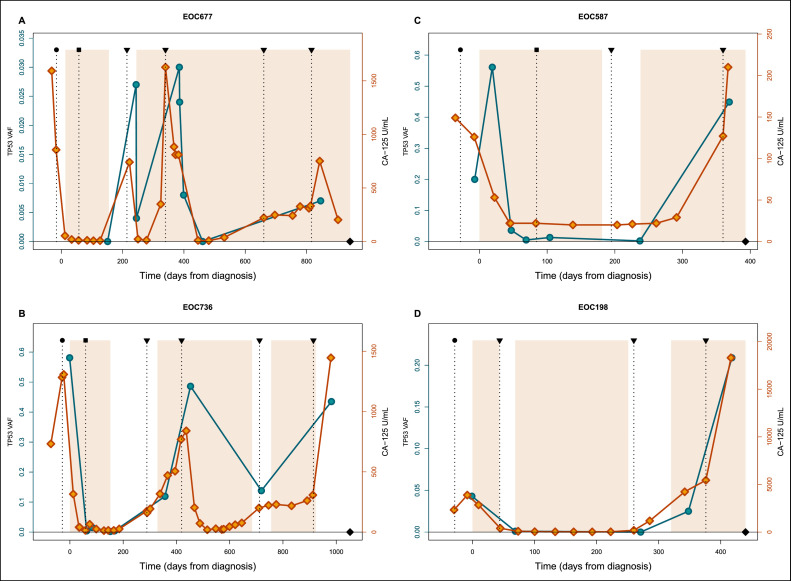
Examples of timelines with CA-125 and *TP53* VAF values from diagnosis to relapse in four HGSC patients. Timelines show *TP53* VAF (blue lines) detected from plasma samples and CA-125 (orange lines) levels for patients EOC677 (**A**), EOC587 (**B**), EOC736 (**C**) and EOC198 (**D**). Legend: black circle - primary operation (PDS or laparoscopy for NACT-patients); black square - IDS; triangle - progression; black diamond - death; the time during chemotherapy are depicted by the coloured areas.

All patients had HGSC with a pathogenic, truncal *TP53* mutation. Therefore we relied on *TP53* VAF, when estimating the tumor content in tissue, ascites, and plasma samples [12] (Fig. 2). Samples with zero *TP53* VAF were considered to have tumor content under the detection limit.

In our longitudinal plasma collection, we had 39 (20 %) ctDNA samples without detectable *TP53* mutation, of which more than half (*n* = 22) were collected either during chemotherapy or right after surgery, and six were follow-up samples without clinical signs of progression. The *TP53* VAF range was 0 % to 99.3 % in tissue samples, and 0 % to 58.1 % in plasma samples. We found no significant variation in the TP53 VAF range in plasma samples between pre-treatment (0.3 % to 58.1 %) and relapse (0.1 % to 55.8 %; *p* = 0.90, *t*-test).

The *TP53* VAF and CA-125 values significantly correlated (*R* = 0.62, *p* < 2.2e-16, Spearman's rank correlation, Figure S1), reinforcing the rationale for utilizing truncal *TP53* mutation VAF as a reliable tool for estimating tumor fractions. For CA-125 values lower than 35 U/mL, i.e.*,* the threshold for normal CA-125 levels, the corresponding plasma samples show an almost undetectable tumor content (range 0 % to 0.031 %, Figure S1). This finding emphasizes the critical role of optimal sampling timing: high-quality samples with substantial tumor fraction are generally obtained when CA-125 is elevated. Examples of patient timelines showing *TP53* VAF and CA-125 are depicted in Fig. 2.

### Mutations detected in ctDNA show a high concordance with tumor tissue

**Fig. 3:**
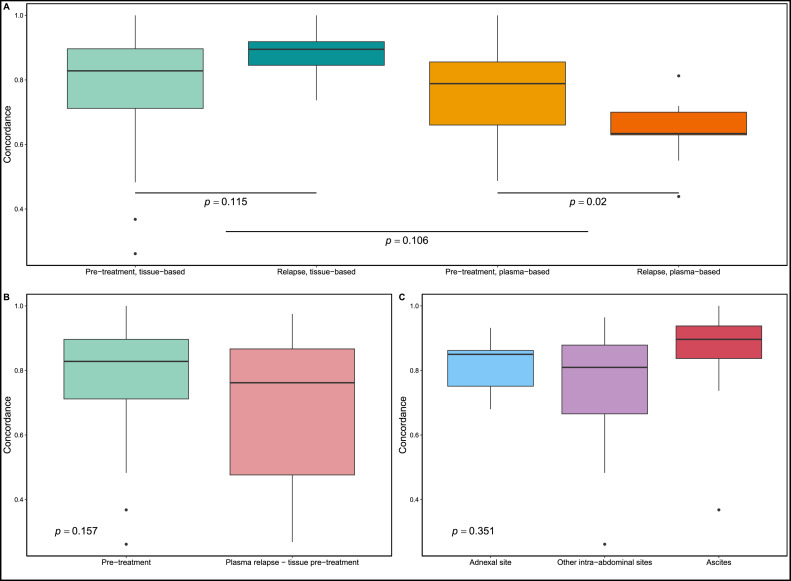
Plasma-tumor concordance analysis. A) Tissue-based and plasma-based concordances at pre-treatment and relapse. Wilcoxon signed-rank test used for two-group comparison and *t*-test for comparison with multiple groups. **B)** Concordances of mutations identified between different time-points and tissue and plasma. **C)** Comparison of concordance of mutations identified in plasma to tissue samples from the adnexa, other intra-abdominal sites and ascites, to determine ctDNA release from different anatomical regions.

We initially examined the reliability of ctDNA in monitoring genomic alterations by determining the proportion of mutations identified in tumor tissue and detected in ctDNA through tissue-based concordance. Detected high median concordance (86.2 %) means that the majority of mutations can be detected through ctDNA, validating the use of genomic information from ctDNA for further analysis. The detection rate was similar at pre-treatment and relapse (Fig. 3A, 82.8 % vs 89.5 %, *p* = 0.115). Tissue-specific mutations undetected in plasma samples had lower VAF than other mutations in tissue: 95 % of them had VAF below 0.09, possibly eluding detection in plasma due to limits in sensitivity. The proportion of functionally relevant mutations (CADD score > 10) was higher in shared mutations (60%  vs 45 %, *p* = 3.2e-05, Fisher's exact test), implying that most driver mutations were successfully identified in plasma samples.

We observed more plasma-unique mutations at relapse than before treatment (Fig. 3A, median plasma-based concordances 63.3%  vs 78.8 %, *p* = 0.02). This result suggests that the disease's heterogeneity is better captured through ctDNA than relapse ascites samples. Additionally, when comparing relapse plasma samples with pre-treatment tumor tissue (Fig. 3B), we observed their tissue-based concordance was not significantly different from that of pre-treatment plasma and tissue (*p* = 0.157, *t*-test). These results show that most pre-treatment mutations remain after primary treatment and unique emerging mutations are found at relapse. This encourages the use of ctDNA in relapse treatment planning.

### ctDNA is similarly released from the adnexa, intra-abdominal metastases and ascites

The impact of the tumor´s anatomical location on ctDNA release was assessed by comparing tissue-based concordance of mutations across three distinct anatomical regions; i) the adnexa, ii) other intra-abdominal sites, such as the bowel mesentery, the omentum and peritoneum, and iii) ascites. High concordances were detected for all three anatomical regions: 85.0 % for the adnexa, 81.0 % for other intra-abdominal sites and 89.6 % for ascites (Fig. 3C). The similar concordances suggest that comparable levels of ctDNA are released from different tissues, proposing that a liquid biopsy represents mutations from various locations.

### Tumor burden in plasma samples during primary treatment reflects patient outcome

There was no significant correlation of ctDNA-derived tumor burden of pre-treatment plasma samples with patient outcome (*R* = 0.03, *p* = 0.88 and *R* = 0.13, *p* = 0.57, Spearman's rank correlation for PFI and OS, respectively). Conversely, a higher tumor burden detected before the third chemotherapy cycle correlates to a less favorable outcome (*R* = −0.72, *p* = 0.008 for PFI and *R* = −0.74, *p* = 0.005 for OS, respectively, Spearman's rank correlation). This suggests that information derived from plasma samples during treatment could serve as a predictor of patient response to primary treatment.

### Changes in mutation profile during treatment

We examined relapse-specific mutations to identify if they were caused by platinum treatment. Contrary to our expectations, the relapse plasma samples did not demonstrate an increased prevalence of mutation types typically associated with platinum exposure, namely C>A and C>T[26], when compared to the pre-treatment samples (*p* = 0.56, Fisher's exact test). Overall, relapse specific mutations were more commonly detected in patients with higher tumor fraction at relapse (≥2 % TF, *p* = 0.042, Fisher's exact test).

**Fig. 4:**
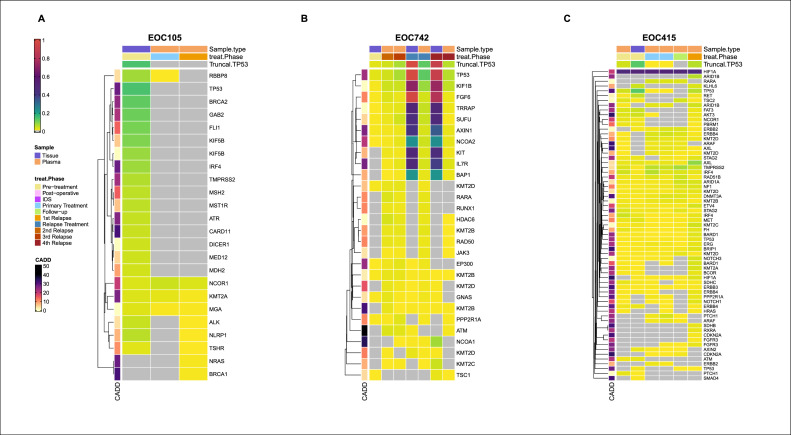
Mutational profiles enable the detection of events in different genes that appear at relapse. Mutation frequencies for patients EOC105, EOC415 and EOC742 are presented as an example. The mutations appearing at relapse were identified in *BRCA1* and *NRAS* for patient EOC105 **(A)**, in *IL7R, RAD50, ATM, KMT2D, RARA, KMT2B* and *RUNX1* for patient EOC742 **(B)** and in *SDHB, FGFR3, ARID1B, CDKN2A* and *RXRA* for patient EOC415 **(C)**.

To monitor the emergence of mutations during treatment, we pinpointed mutations that were undetectable in all samples prior to relapse. Screening for functionally relevant mutations resulted in 25 events appearing at relapse in eight patients: one was a frameshift deletion, two were stop-gain variants, 17 nonsynonymous, four synonymous SNVs, and one was an intronic mutation (Table 2, examples in Fig. 4). We assessed further the pathogenicity of the 25 relapse-appearing mutations from the Molecular Tumor Board database [25]. Variants classified as “Predicted pathogenic”, “Pathogenic”, “Likely pathogenic” or “Oncogenic” were considered pathogenic, resulting in 19 pathogenic relapse-appearing mutations in genes *KCNH2, JAK2, GRIN2A, FGFR3, ARID1B, CDKN2A, RXRA, FLT4, FLT3, TGFBR2, TBX3, NRAS, BRCA1, NCOA1, IL7R, RAD50, ATM, KMT2B*, and *SMO*.

**Table 2:** Complete list of the 25 relapse-appearing mutations. Variant and protein change information are derived from the MANE selected transcript for the corresponding gene; Function and Treatment are derived from the Molecular Tumor Board database.

Patient	Gene	Variant	Protein change	Function	Reported Treatment
EOC677	*KCNH2*	c.2905G>*A*	p.G969S	Predicted pathogenic	–
EOC677	*JAK2*	c.1849G>*T*	p.V617F	Pathogenic	Peginterferon Alfa-2b
EOC677	*GRIN2A*	c.1505A>*T*	p.Y502F	Predicted pathogenic	–
EOC415	*SDHB*	c.72G>C	p.Q24H	Uncertain significance	–
EOC415	*FGFR3*	c.1612A>*G*	p.I538V	Pathogenic	Erdafitinib; Gemcitabine - Cisplatin; Infigratinib
EOC415	*ARID1B*	c.1511A>*G*	p.Q504R	Predicted pathogenic	–
EOC415	*CDKN2A*	c.341C>T	p.P114L	Oncogenic	Abemaciclib; Palbociclib; Ribociclib
EOC415	*RXRA*	c.332T>*A*	p.L111Q	Predicted pathogenic	–
EOC736	*FLT4*	c.3736A>*T*	p.T1246S	Predicted pathogenic	–
EOC740	*FLT3*	c.1172C>T	p.P391L	Predicted pathogenic	–
EOC1120	*TGFBR2*	c.766G>*A*	p.A256T	Predicted pathogenic	–
EOC1120	*TBX3*	c.1310C>T	p.P437L	Predicted pathogenic	–
EOC105	*NRAS*	c.52G>*A*	p.A18T	Likely pathogenic	–
EOC105	*BRCA1*	c.5095C>T	p.R1699W	Pathogenic	Niraparib; Olaparib; Bevacizumab;Rucaparib
EOC742	*NCOA1*	c.669T>*A*	p.C223X	Predicted pathogenic	–
EOC742	*IL7R*	c.805A>*G*	p.K269E	Predicted pathogenic	–
EOC742	*RAD50*	c.2178T>*G*	p.R726R	Predicted pathogenic	–
EOC742	*ATM*	c.1075G>*T*	p.E359X	Predicted pathogenic	Olaparib
EOC742	*KMT2D*	c.6273C>T	p.G2091G	Likely neutral	–
EOC742	*KMT2D*	c.2066T>C	p.L689P	Likely neutral	–
EOC742	*RARA*	–	–	–	–
EOC742	*KMT2B*	c.3052delA	p.G1020Afs*4	Predicted pathogenic	–
EOC742	*RUNX1*	c.720A>C	p.P240P	Likely neutral	–
EOC295	*SMO*	c.1303G>*A*	p.G435R	Predicted pathogenic	–
EOC295	*KRAS*	c.217A>C	p.R73R	Likely neutral	–

The 19 pathogenic relapse-appearing mutations were enriched in several cancer-associated pathways. Notably, among them was *Pathways in cancer* (KEGG, FDR-corrected *p* = 0.059), a complex molecular network of events involved in various cellular processes, whose dysregulation is often observed in cancer. Additionally, we observed the enrichment of *PI3K-Akt signaling pathway* (FDR-corrected *p* = 0.059) that has been reported to play a major role in multidrug resistance, due to its activity in multiple processes, like regulation of apoptotic processes and induction of ABC transporters [27].

Screening for drug targets among events appearing at relapse in the plasma samples revealed the presence of four putative functionally relevant variants, namely missense, p.Arg1699Trp, in *BRCA1*, missense p.Pro114Leu, in *CDKN2A*, missense p.Ile538Val, *FGFR2* and missense p.Val617Phe, in *JAK2*.

Patient EOC105 (Fig. 4A) was known to have a somatic *BRCA2* mutation in the primary tumor sample and initially responded well to platinum-based chemotherapy. Curiously, we found a *BRCA1* mutation (p.R1699W) emerging at first relapse. She was treated again with platinum-taxane followed by Niraparib as maintenance therapy for two years without disease progression.

Patient EOC415 (Fig. 4C) underwent platinum-taxane chemotherapy following primary debulking surgery, showing a favorable response. This was followed by bevacizumab as maintenance therapy. At first and second relapse, she received additional platinum-taxane, and progressed during the last chemotherapy. At relapse she manifested novel mutations in *FGFR3* and *CDKN2A*. Given these mutations, a receptor tyrosine kinase inhibitor [28] could be a future option for patients with similar mutations and platinum resistance.

## Discussion

In this study, we analyzed an extensive longitudinal ctDNA dataset from HGSC patients with the comprehensive gene panel. Research into recurrent HGSC has the potential of identifying druggable mutations when standard care fails and is hence essential in enhancing patient survival. Chemotherapy and evolving resistance mechanisms modify the tumor resulting in altered genomic profiles [14,15]. Our study suggests that ctDNA plasma samples taken at relapse not only portray the primary lesion adequately, but also represent the evolved metastatic lesion better than a sample from the primary tumor tissue or relapse ascites. The emergence of new mutations present opportunities for individualistic therapy options when standard care has failed.

Previous studies on ctDNA in HGSC have been conducted using smaller gene-panels or focusing only on *TP53* [12,29]. Additionally, HGSC is often underrepresented in larger pan-cancer studies [30]. Hence, there has been a need for a more in-depth ctDNA analysis of HGSC, essential for validating the use of ctDNA as a biomarker [31]. Given the intrinsic heterogeneity of HGSC and the scarcity of recurrently mutated genes, the use of a broad and comprehensive gene panel was essential in our analysis. With the extensive panel we showed a high plasma to tumor tissue concordance, achieving a high detection rate exceeding 80 % at both pre-treatment and relapse. The significantly lower plasma-based concordance at relapse than at pre-treatment time-point, highlights the increasing genomic variance during recurrence. Thus, ctDNA appears to offer a more comprehensive perspective on the disease's heterogeneity at relapse than ascites-derived samples. Notably, we were able to detect mutations identified in tissues in plasma samples without detectable *TP53* mutation. This underlines the importance of targeting a larger number of mutations through a large panel when tumor content is small, which was common in HGSC patients in our cohort.

Studies using next generation sequencing on urothelial cancer [32] and GIST-tumors [33] show that different anatomical regions often provide varying genetic information in tissue biopsy. This obstacle could be overcome by using a ctDNA plasma sample since tumor-DNA is shed into the bloodstream from all locations [9]. While Bando *et al*. found that ctDNA levels in metastatic colorectal cancer are influenced by the metastasis site [34], Here we showed, for the first time in HGSC, that ctDNA is released similarly from different anatomical regions, suggesting that a ctDNA plasma sample can be used to represent the metastatic cancer regardless of tumor location.

The amount of ctDNA as a prognostic marker would be a clinically relevant tool. Parkinson *et al*., found that a low pre-treatment *TP53* VAF at relapse was associated with a significantly longer time to progression [10]. In contrast, we showed that pre-treatment ctDNA levels did not have a significant prognostic value, which is in line with the study of Pereira et al. [35]. However, our results presented significant negative correlations with *TP53* VAF detected before the third cycle of primary chemotherapy to PFI and OS. Similarly, Pereira *et al*. showed that undetectable levels of ctDNA after initial treatment predicted PFI and using ctDNA could be more accurate in predicting relapse than the currently used CA-125 and radiological imaging [35]. Additionally, Cohen *et al*. reported the clinical utility of ctDNA in detecting molecular residual disease and its connection to patient outcomes in multiple cancers [36]. In summary, these results highlight the potential of ctDNA as a prognostic marker of HGSC during or after treatment. Further studies with larger cohorts are needed to fully implement the use of ctDNA into clinical practice.

Individualized treatment options at relapse can profoundly impact patient wellbeing. Previously, we have shown that longitudinal patient sampling reveals alterations in genomic expression to optimize treatment [17]. In the current study, we identified 19 pathogenic mutations appearing at relapse in eight patients, providing possible drug targets. These include for example HRD-related mutations with implication to PARP inhibitors. Additionally, PI3K-Akt signaling mutations were enriched at relapse. This pathway plays pivotal roles in various cellular processes critical to cancer development, such as growth, survival, and proliferation [34]. Notably, alterations in the PI3K-Akt pathway are found in chemoresistant malignancies [25], offering potential drug targets [35]. For example, recent studies have shown that the modification of the PI3K-Akt pathway is necessary for *HER2* mediated tumorigenesis [36], which is targetable with trastuzumab that is in routine clinical use in breast cancer.

One limitation of our study is the small number of plasma samples at later relapses, where novel mutations could show possible drug targets. It is also important to acknowledge that we cannot definitively determine whether mutations found only in the relapse ctDNA arose during disease progression or if they simply became more prevalent, thus surpassing the detection threshold. A more thorough selection of samples prior to analysis, would have made the patients more comparable and possible similarities more accessible. Additionally, the inherent heterogeneity of HGSC hinders wider generalization of findings from 29 patients.

However, our study's strengths lie in the longitudinal ctDNA sampling from an unselected HGSC patient cohort, combined with sequencing using an extensive gene panel targeting cancer-related protein-coding genes. This shows the potential of liquid biopsy in informing clinical decisions and suggesting therapeutic interventions. The upgraded mutation discovery pipeline we applied (Data Supplement) enabled us to more precisely detect true positive alterations, reducing the overall noise in mutational profiles. Importantly, the sequencing coverage of 1000x, utilized in this work, is sufficient since we focused on the detection of genomic events during timepoints of clinically detectable tumor. The amount of ctDNA detected from plasma samples is consistent with other studies [37], making the results reliable and significant.

In conclusion, when a HGSC patient has relapsed, liquid biopsy offers an optimal alternative for tumor profiling. Since the recurrent tumor has undergone genetic changes, ctDNA covers the heterogenic disease better than pre-treatment tissue or relapse ascites.

## CRediT authorship contribution statement

**Giovanni Marchi:** Formal analysis, Writing – review & editing, Writing – original draft. **Anna Rajavuori:** Data curation, Writing – review & editing, Writing – original draft. **Mai T.N. Nguyen:** Formal analysis, Writing – review & editing. **Kaisa Huhtinen:** Writing – review & editing, Resources. **Sinikka Oksa:** Writing – review & editing, Resources. **Sakari Hietanen:** Writing – review & editing, Resources. **Sampsa Hautaniemi:** Writing – review & editing, Funding acquisition, Supervision, Conceptualization. **Johanna Hynninen:** Writing – review & editing, Resources, Funding acquisition, Writing – original draft, Supervision, Conceptualization. **Jaana Oikkonen:** Writing – review & editing, Writing – original draft, Supervision, Conceptualization.

## Declaration of Competing Interest

The authors declare that they have no known competing financial interests or personal relationships that could have appeared to influence the work reported in this paper.

## Appendix Supplementary materials

Image, application 1

Image, application 2

Image, application 3
