# Supplementary material for: Extensive mutational ctDNA profiles reflect High-grade serous cancer tumors and reveal emerging mutations at recurrence
Source: Transl Oncol. 2023 Nov 2;39:101814. doi: 10.1016/j.tranon.2023.101814 (PMC10641709; doi:10.1016/j.tranon.2023.101814)
Supplement: Supplementary file 3 [file mmc3.docx]

**Extensive mutational ctDNA profiles reflect High-grade serous cancer tumors and reveal emerging mutations at recurrence**

SUPPLEMENTARY MATERIAL

Giovanni Marchi^1^*, Anna Rajavuori^2^*, Mai T.N. Nguyen^1^, Kaisa Huhtinen^1^, Sinikka Oksa^3^, Sakari Hietanen^2^, Sampsa Hautaniemi^1^, Johanna Hynninen^2#^, Jaana Oikkonen^1#^

*,# These authors contributed equally

^1^Research Program in Systems Oncology, Faculty of Medicine, University of Helsinki, Helsinki 00291, Finland

^2^Department of Obstetrics and Gynecology, University of Turku and Turku University Hospital, 20521 Turku, Finland

^3^Satasairaala Central Hospital, Department of Obstetrics and Gynecology, 28500 Pori, Finland

Corresponding author:

**Jaana Oikkonen**

Research Program in Systems Oncology

Faculty of Medicine
P.O. Box 63
FI-00014 University of Helsinki

Tel. +358405493094

jaana.oikkonen@helsinki.fi

**Experimental Procedures**

**Data processing**

Sequenced DNA reads initially underwent quality control and trimming steps using FastQC [1] and Trimmomatic [2]; good quality reads were then aligned to the reference human genome GRCh38.d1.vd1 using BWA-MEM [3] with default parameters, subjected to deduplication with Picard tool [4] and base quality recalibration using the Genome Analysis Toolkit (GATK) version 4.1.9.0 [5]. Cross-sample contamination was finally estimated with GATK 4.1.9.0 as well: 10 % and 0.5 % for tissue and plasma samples respectively were set as contamination estimation thresholds.

**Mutation calling**

Somatic single nucleotide variants (SNVs) and indels were detected using Somatic Alterations in Genome (SAGE) tool [6] version 2.8 in a paired tumor-normal mode with default parameters disabling base quality recalibration including HTMF hg38 panel, high confidence and hotspot resources (<https://nextcloud.hartwigmedicalfoundation.nl/s/LTiKTd8XxBqwaiC?path=%2FHMFTools-Resources%2FDNA-Resources>) for corresponding arguments. Alterations with PASS filter were kept.

**Mutation filtering**

Once called, the somatic alterations underwent a filtering procedure to clean out the likely spurious, germline related and the mutations occurring with repeated sequences. First, alterations outside the merged Oseq target panels were filtered out. Then, a Panel of Normals (PoN) was used to remove systematic sequencing artefacts. Initially, mutation callings per patient were performed with SAGE in a tumor only mode using, individually, the control samples from the 29 HGSC patients sequenced with Oseq panel: SAGE was run with default parameters with the exception of 6 hard_min_tumor_raw_alt_support, 30 hard_min_tumor_raw_base_quality and disabling base quality recalibration. PoN was then created as a result of the aggregation of the single variant calling format (VCF) files (using HTMF tool <https://github.com/hartwigmedical/hmftools/tree/715254e226100220e8201022c3172c965ae341e5/sage/src/main/java/com/hartwig/hmftools/sage/pon>) and it was used to annotate each patient’s somatic alterations VCF with PON_MAX and PON_COUNT fields, the maximum read support in any one sample and the frequency of each variant in PoN respectively. PON_MAX and PON_COUNT fields were finally used to filter mutations applying different thresholds, depending on whether the single somatic alteration bears the PANEL, HIGH_CONFIDENCE or HOTSPOT field. Resulting mutations were then filtered to keep only events with variant allele frequency (VAF) < 0.4 % in the normal control sample and were annotated using Annovar (version 2019-10-24) with refSeq [7], MANE [8], cytoband [9], COSMIC v3.2 [10], Clinvar [11], dbnsfp [12], 1000 genomes [13], gnomAD [14] databases, and the CADD functional scores [15]. A clustering-based filter was applied to remove false positive variant calls, through identifying events with similar VAF patterns through sampling timeline. Clustering on VAF was performed using the K-means method (number of clusters = 800) and clusters including > 40 % of alterations that were shared in at least three patients and were not listed in the COSMIC database were excluded. Resulting SNVs and indels were cleaned from events appearing within repeated sequences according to RepeatMasker [16] and WindowMasker [17] databases, white-listing events appearing in exonic regions of *TP53* and with a CADD score > 10. Finally, somatic mutations were considered reliable if detected with at least 100 read depth.

**Additional figures**

**Figure S1.** ***TP53* VAF and CA-125 correlation**. CA-125 values are log10-scaled. Samples in each data-point are collected no longer than three days apart. Spearman's rank correlation statistics are shown.


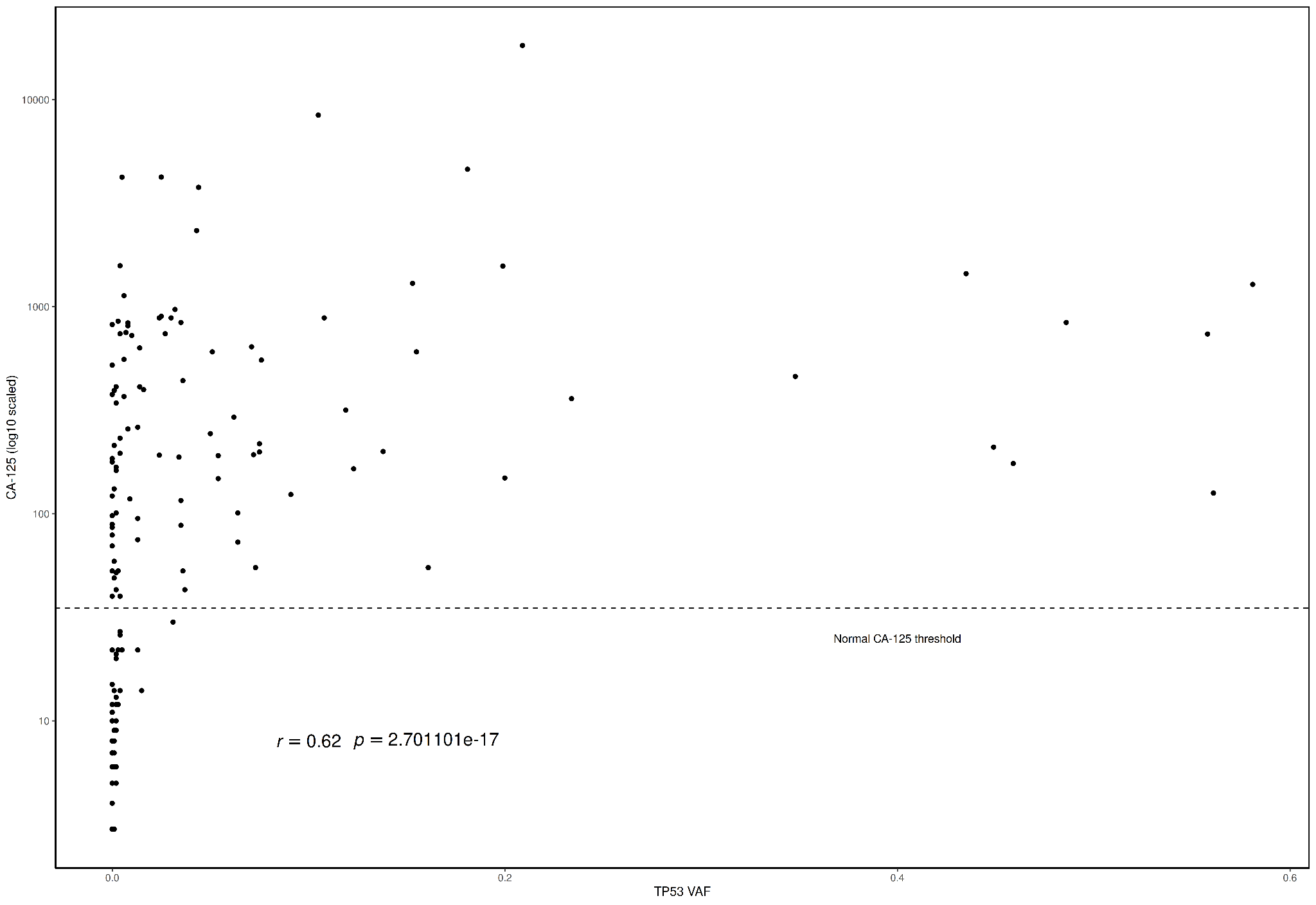


**Figure S2** Patient treatment timelines showing *TP53* VAF (blue lines) detected from plasma samples and CA-125 (red lines) levels. Coloured areas represent chemotherapy treatment intervals. IDS - interval debulking surgery

| 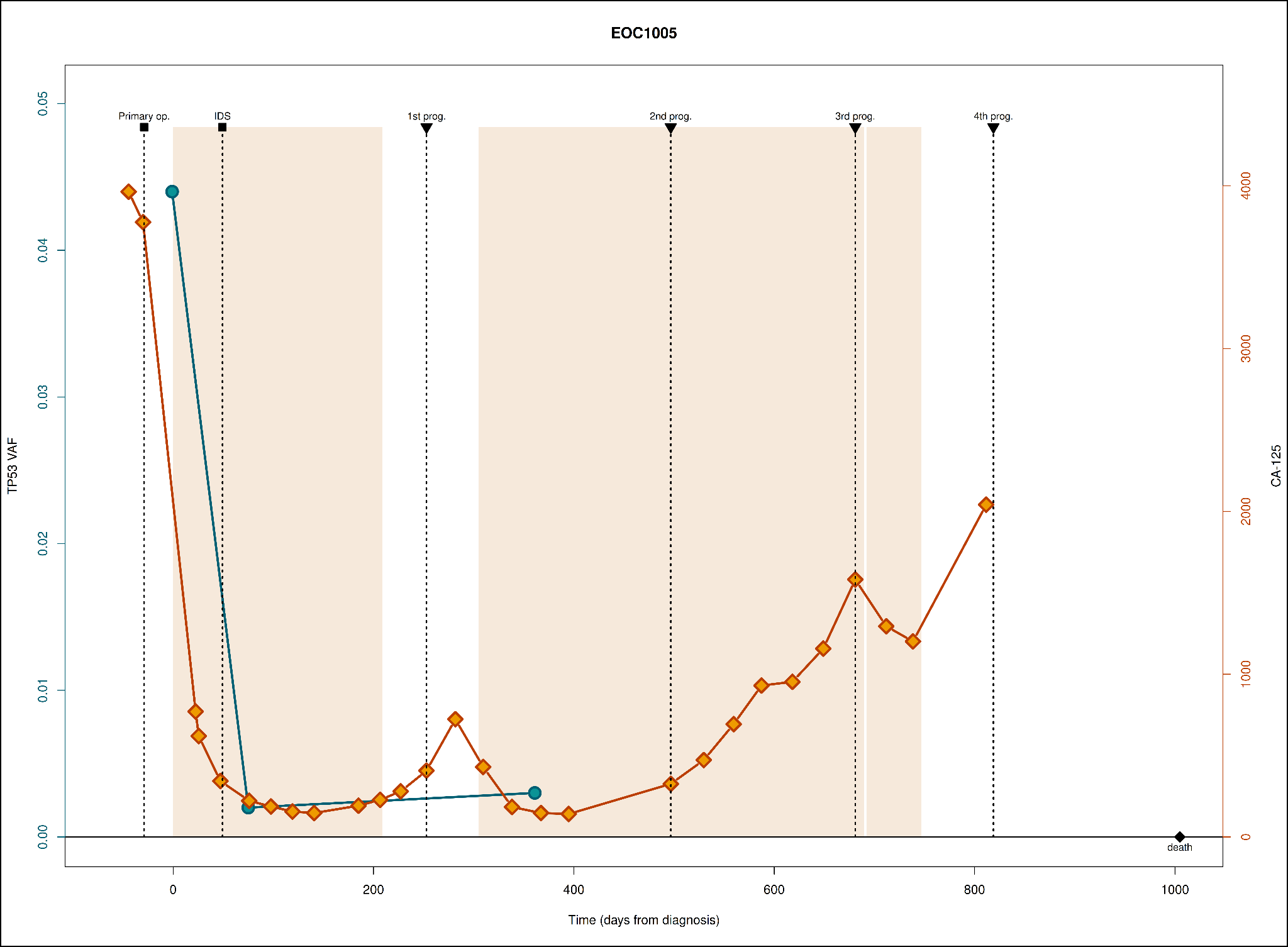 | 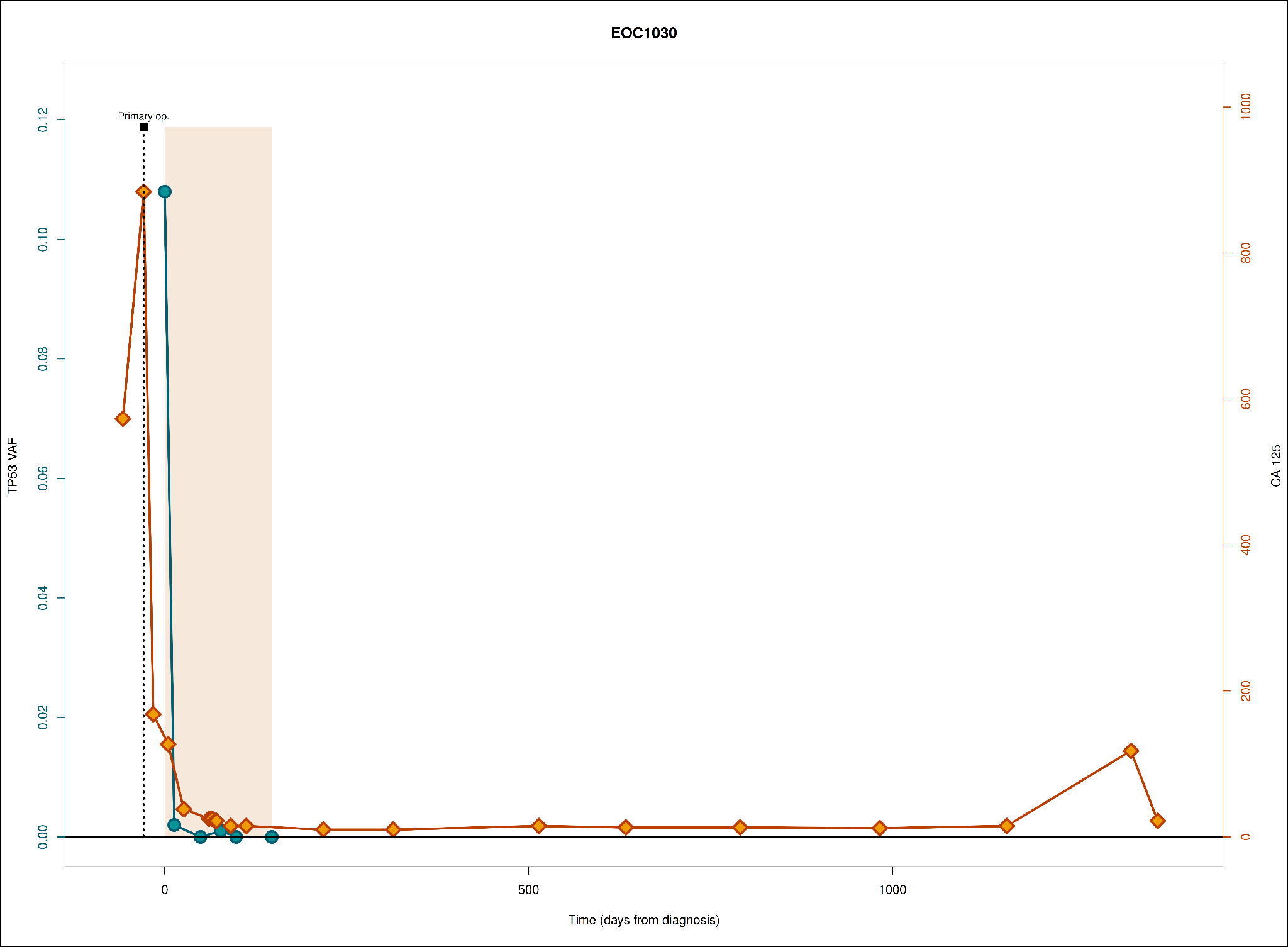 |
| --- | --- |
| 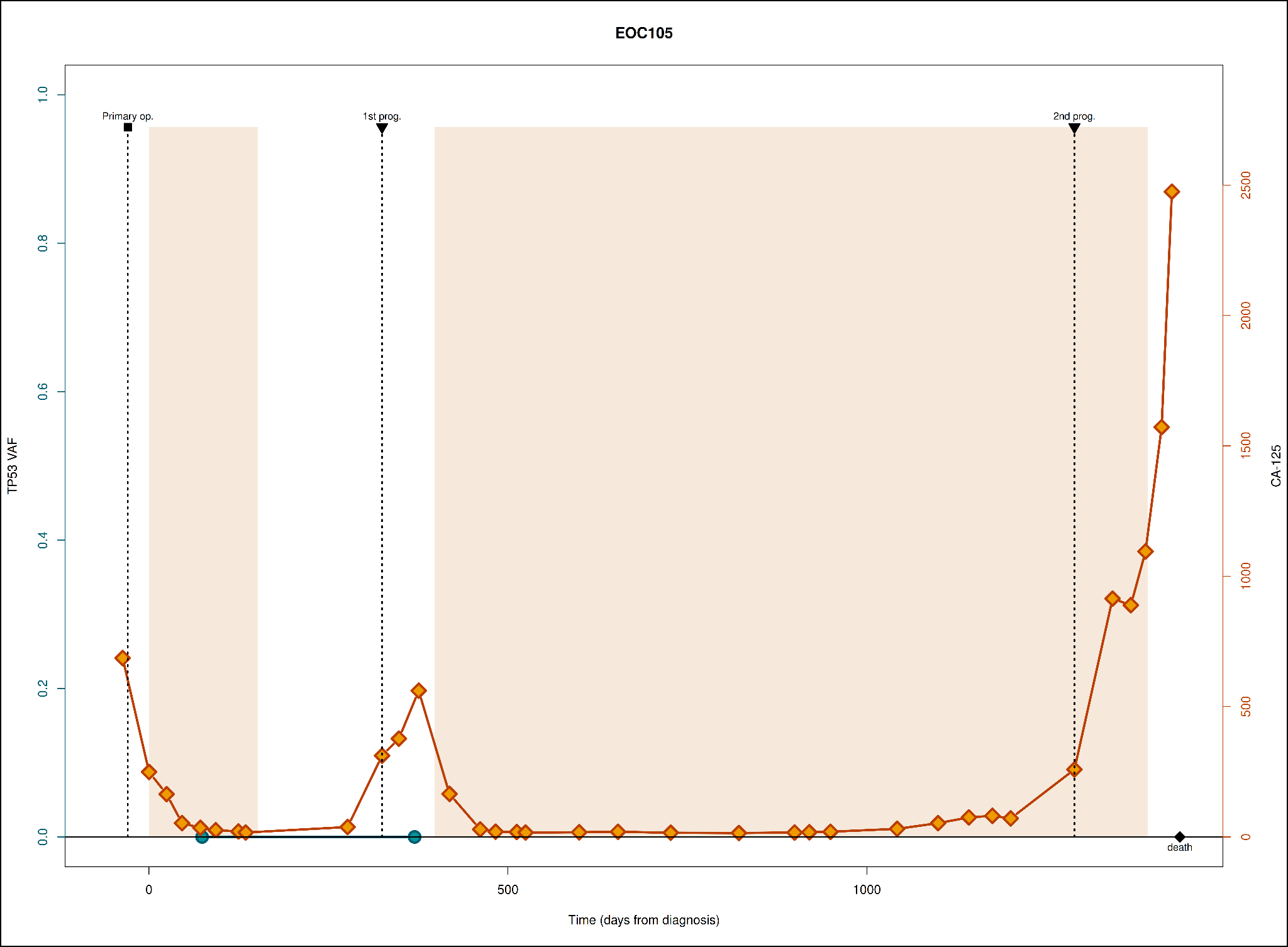 | 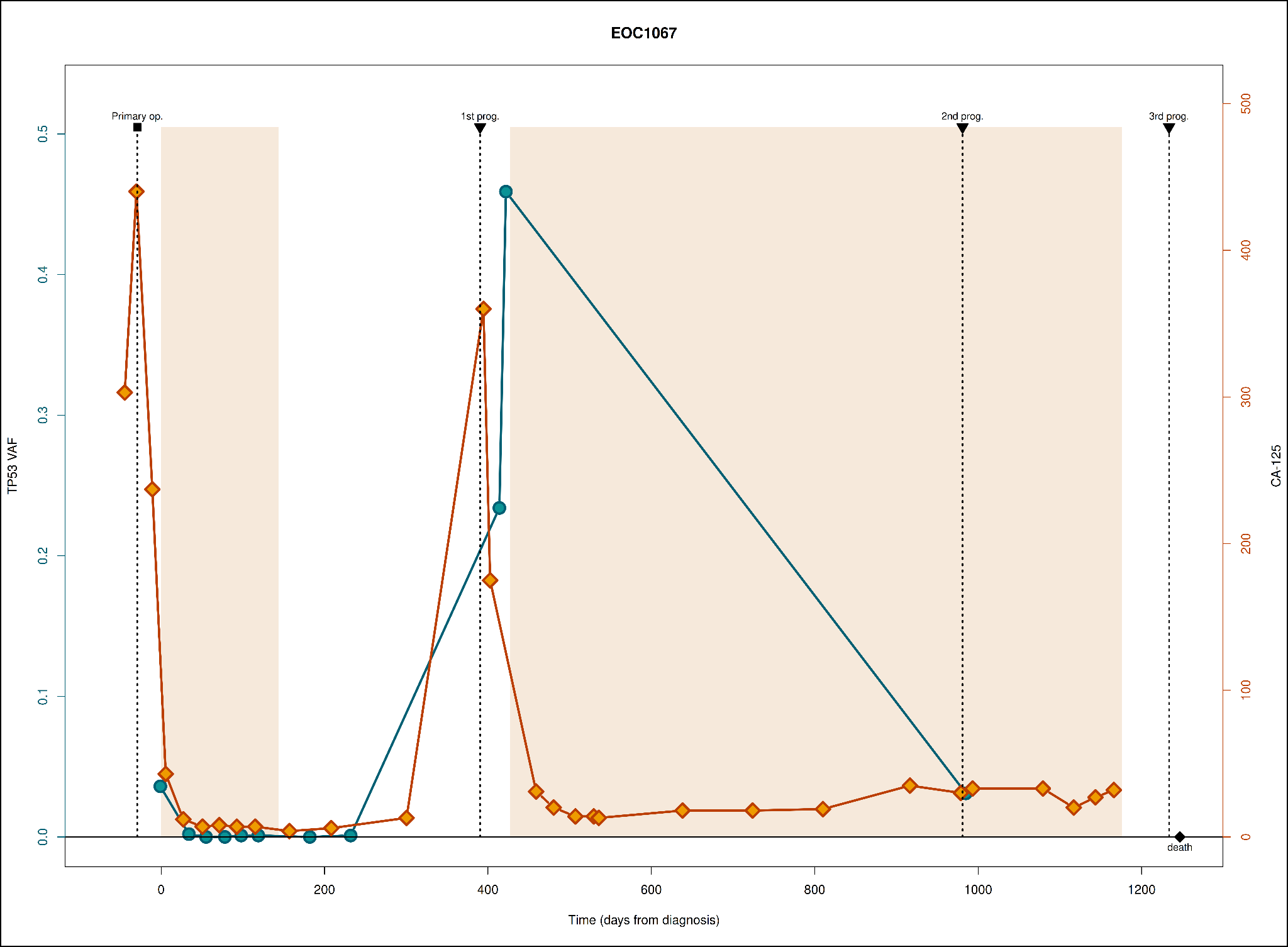 |
| 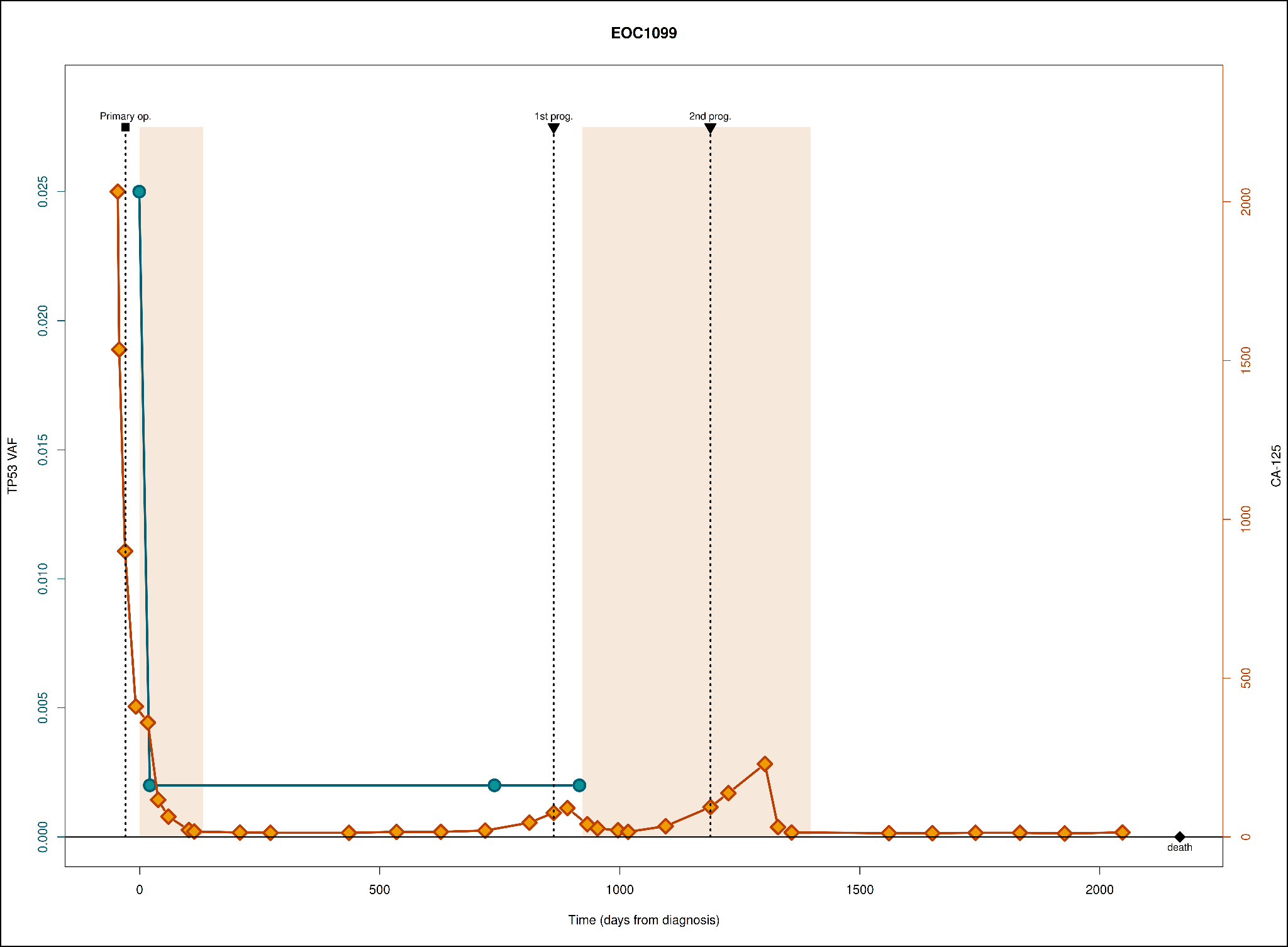 | 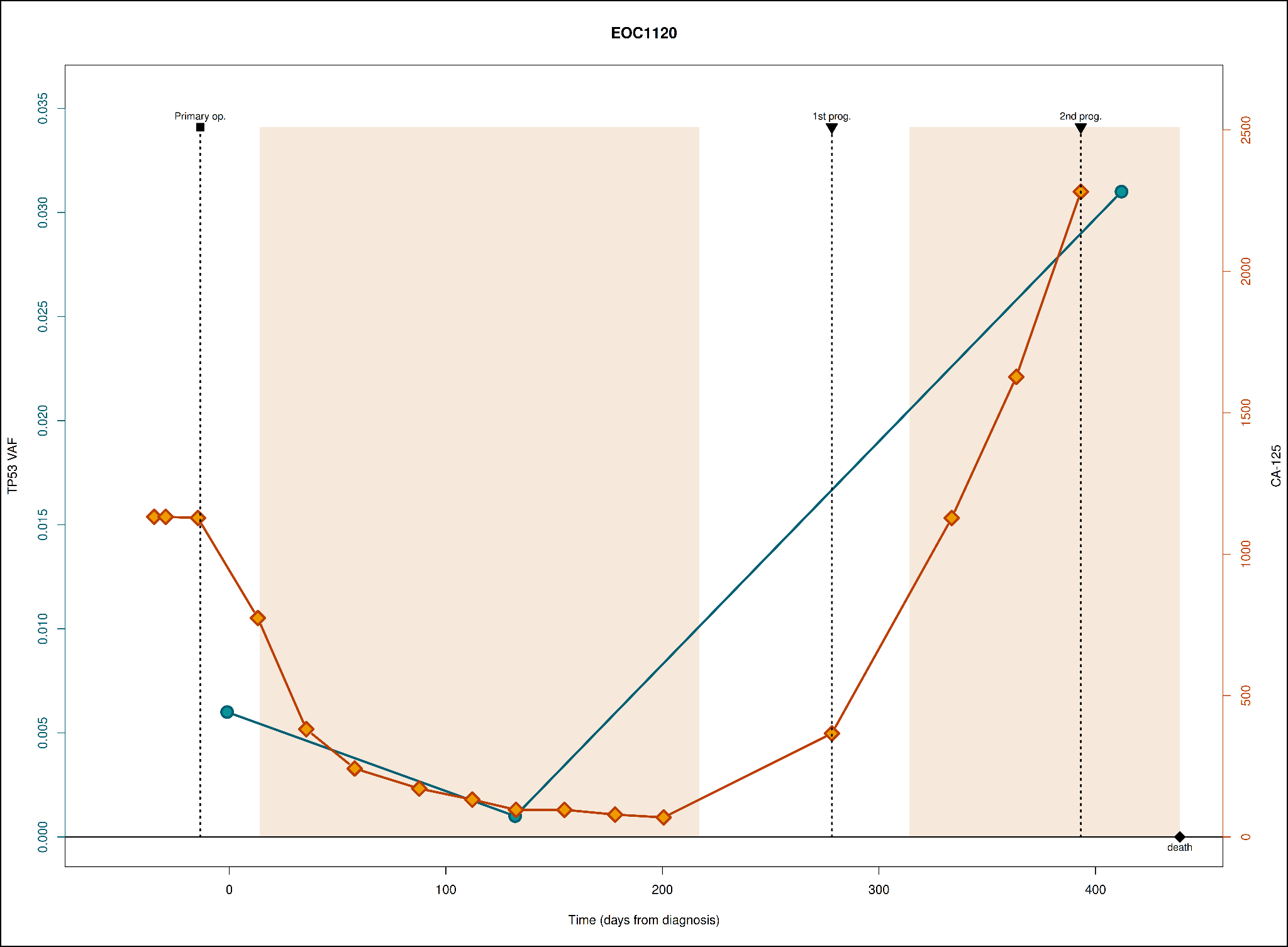 |
| 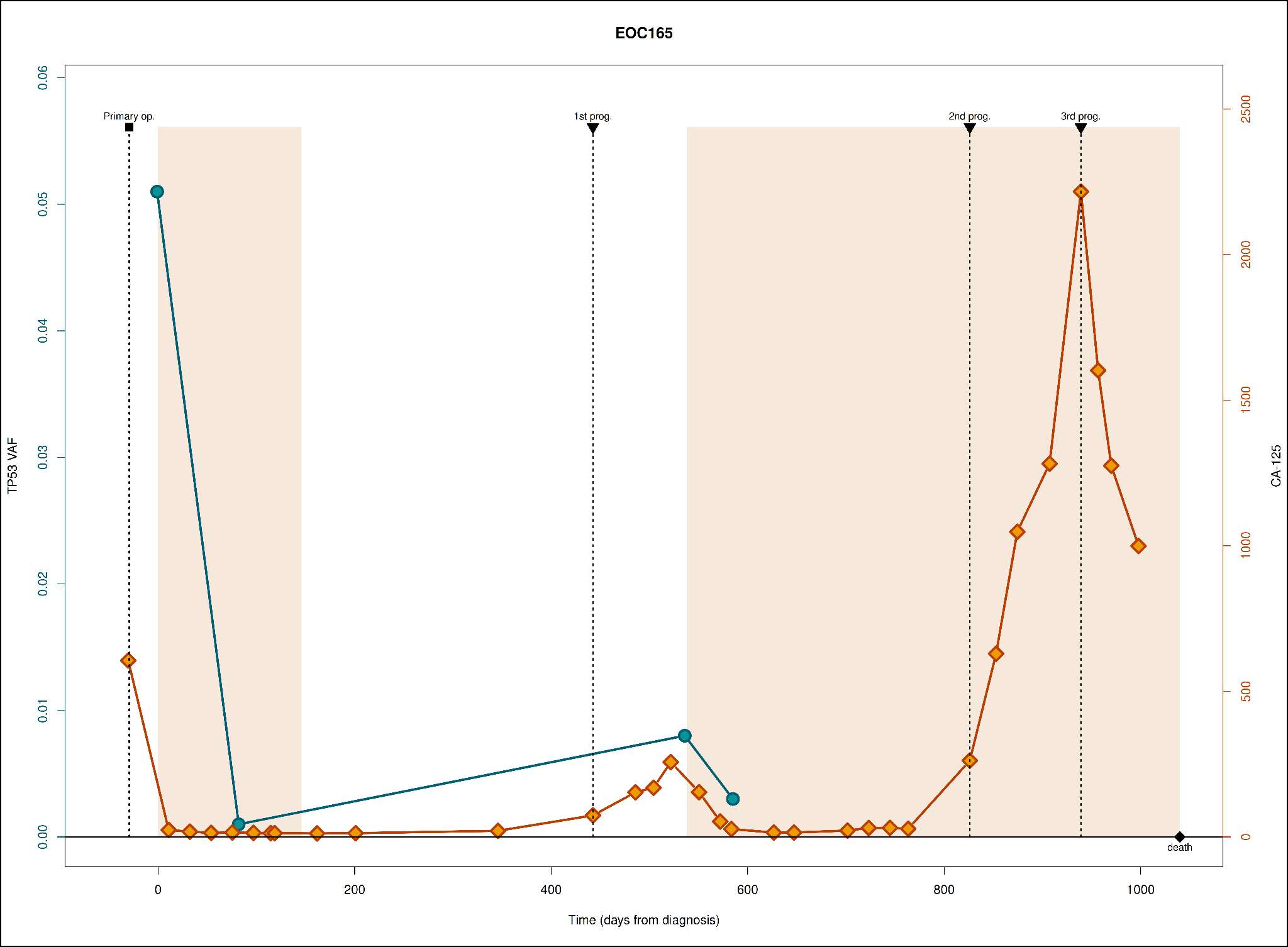 | 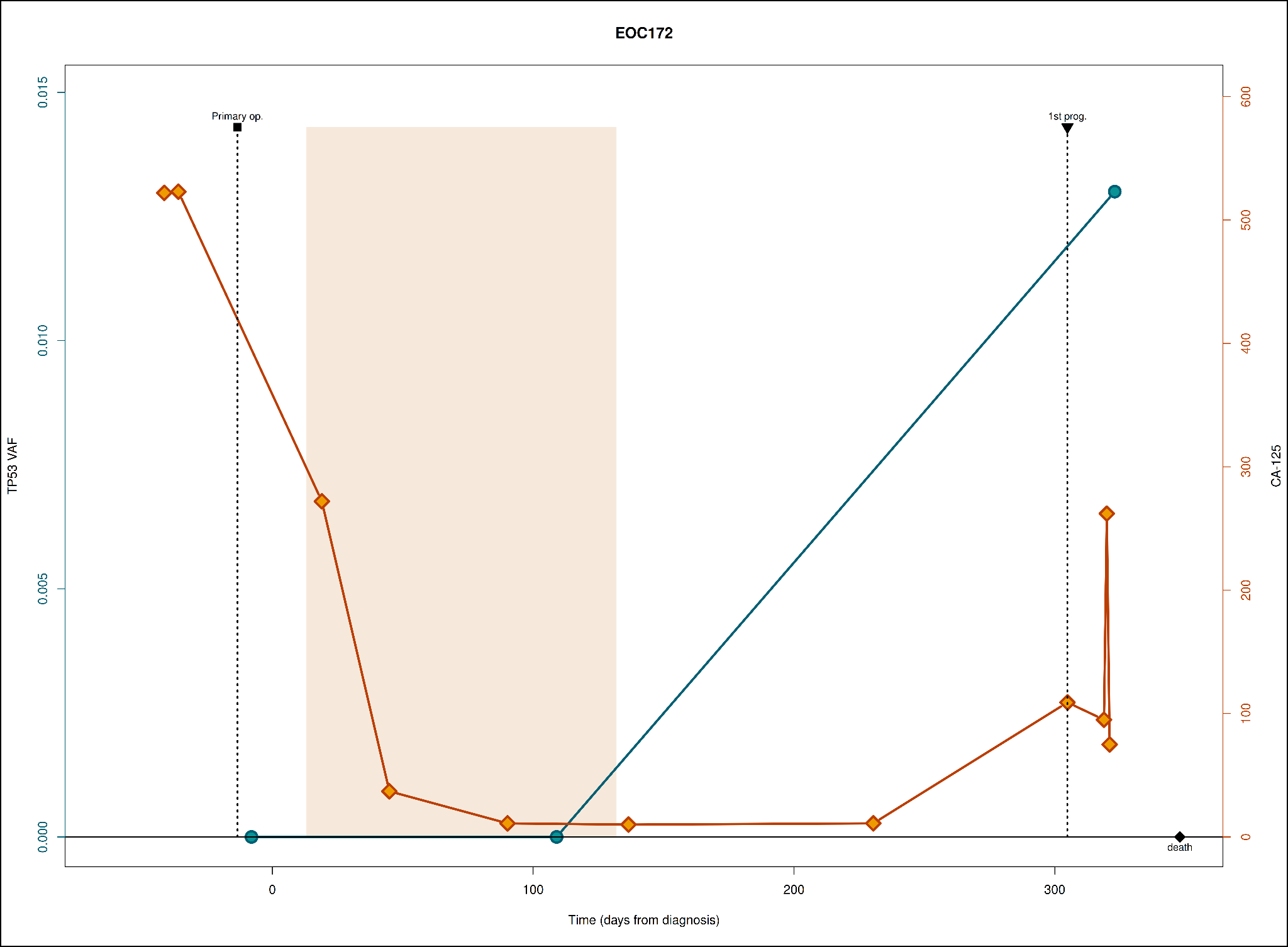 |
| 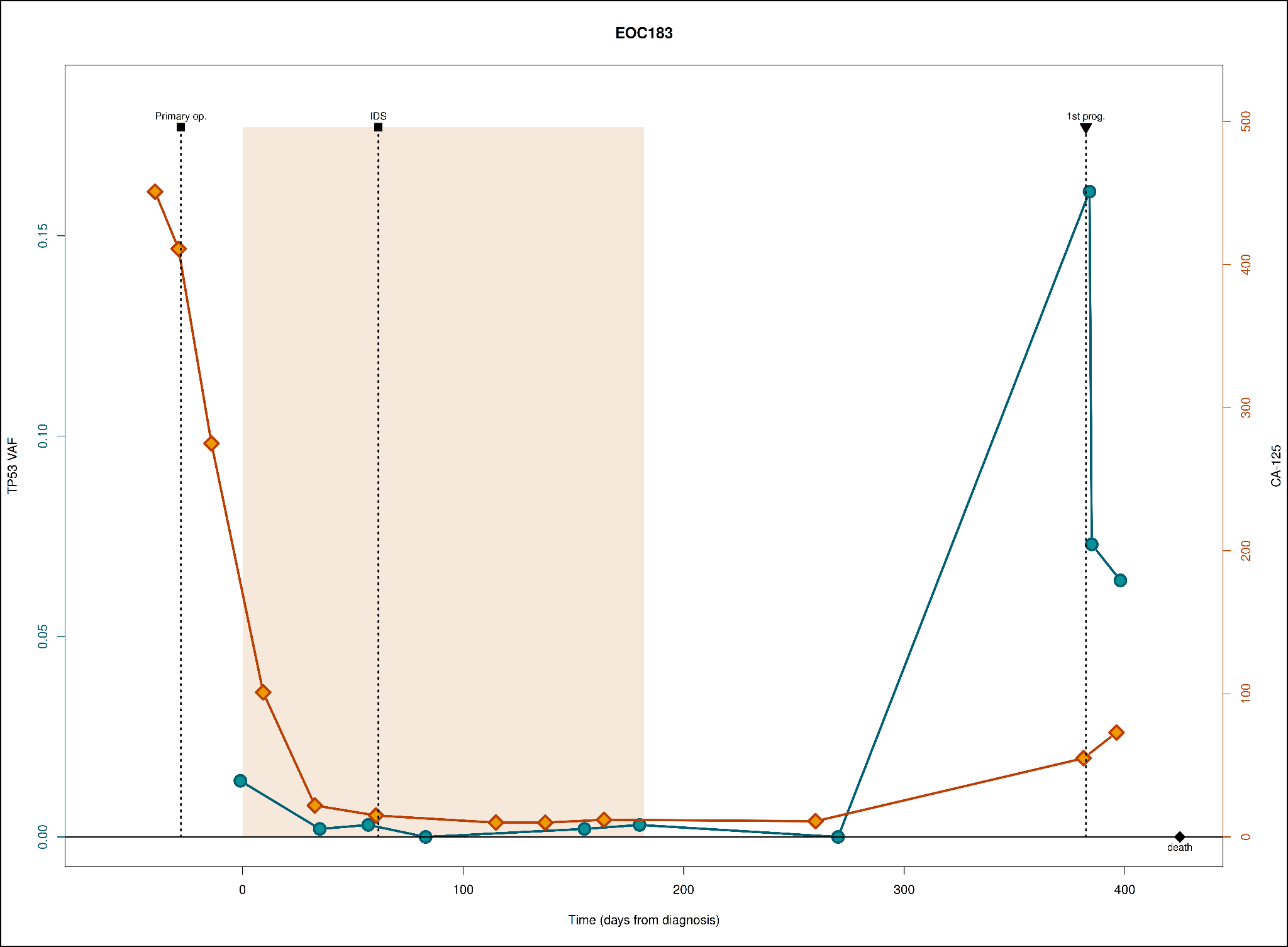 | 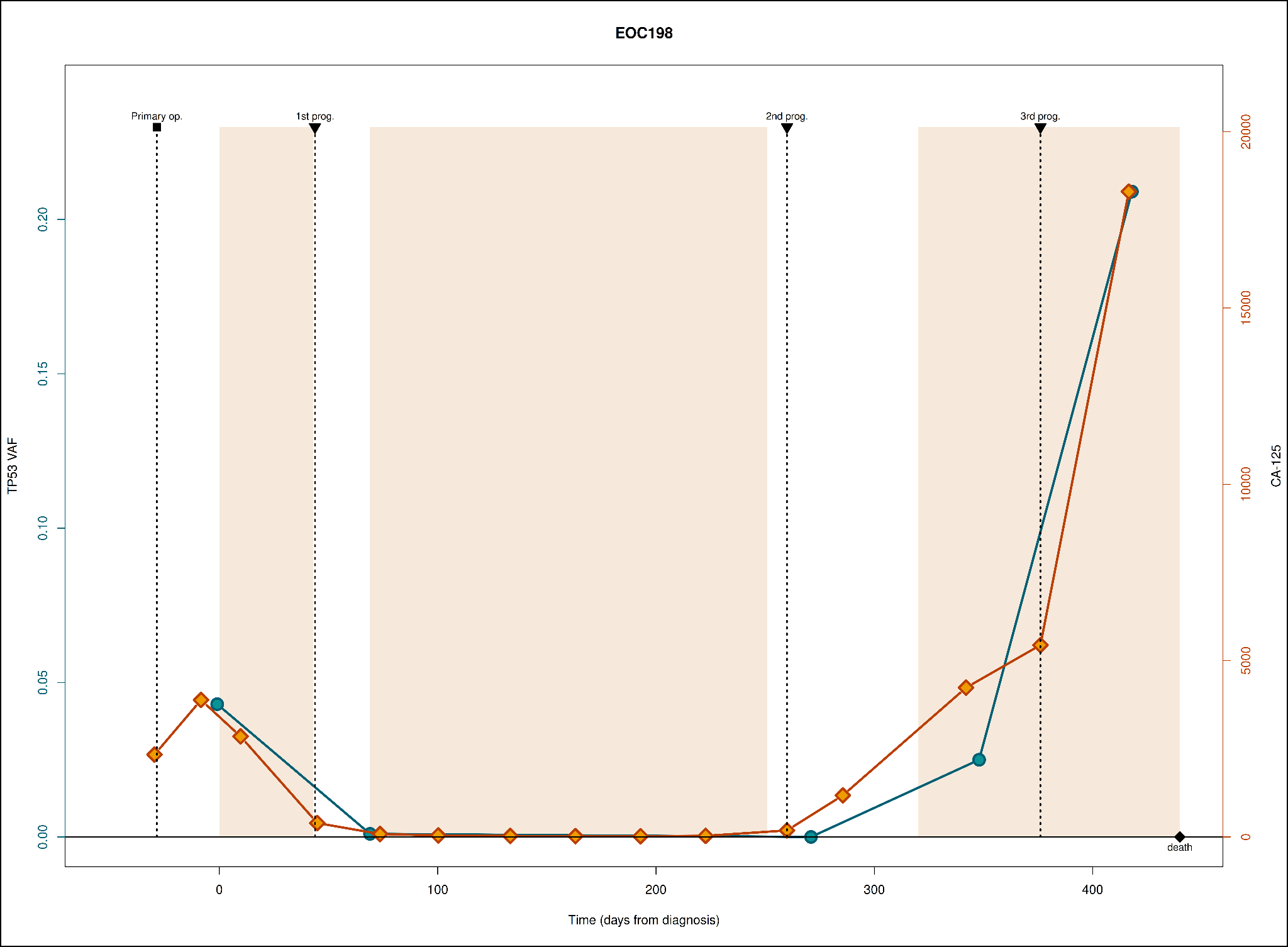 |
| 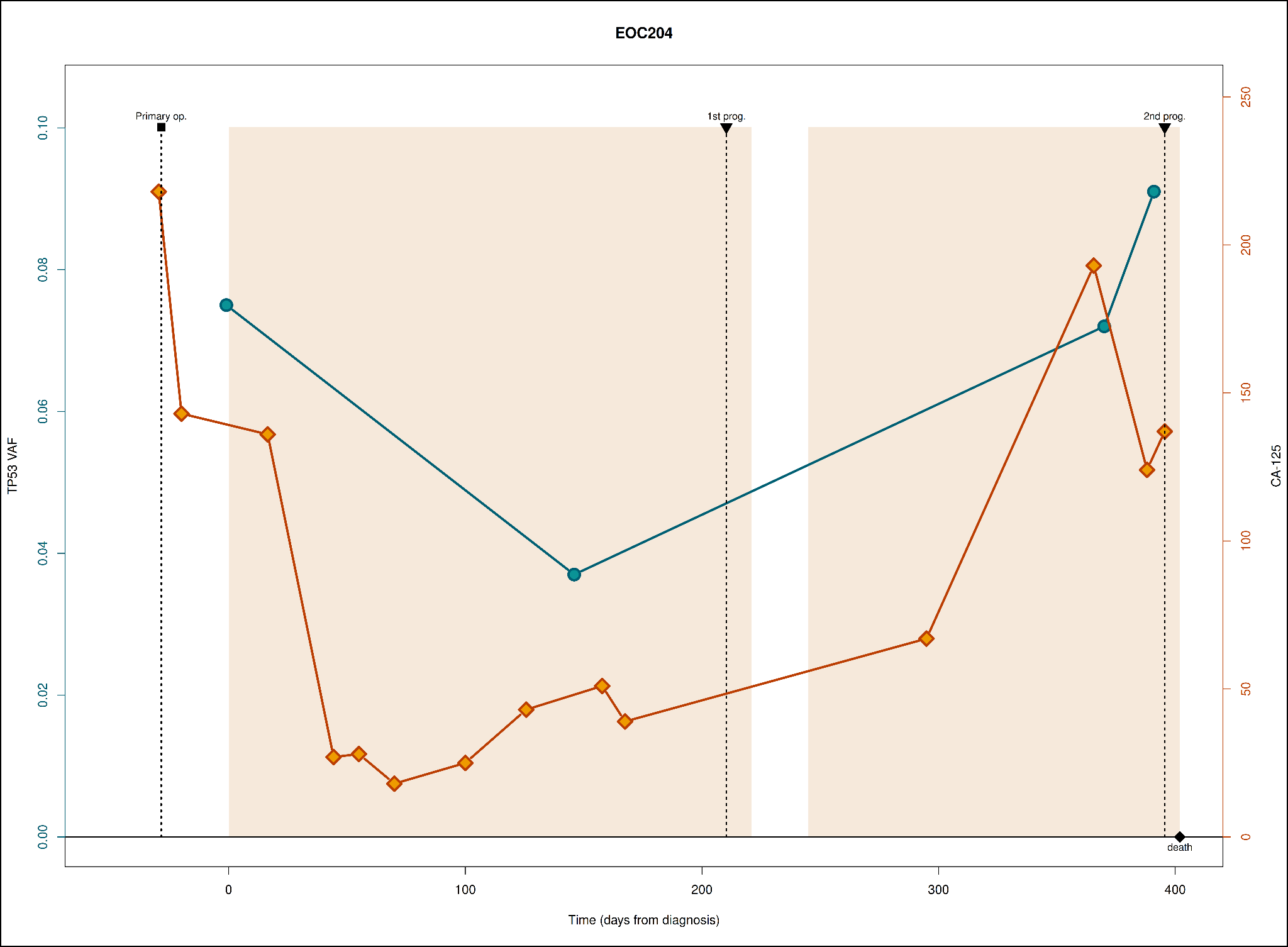 | 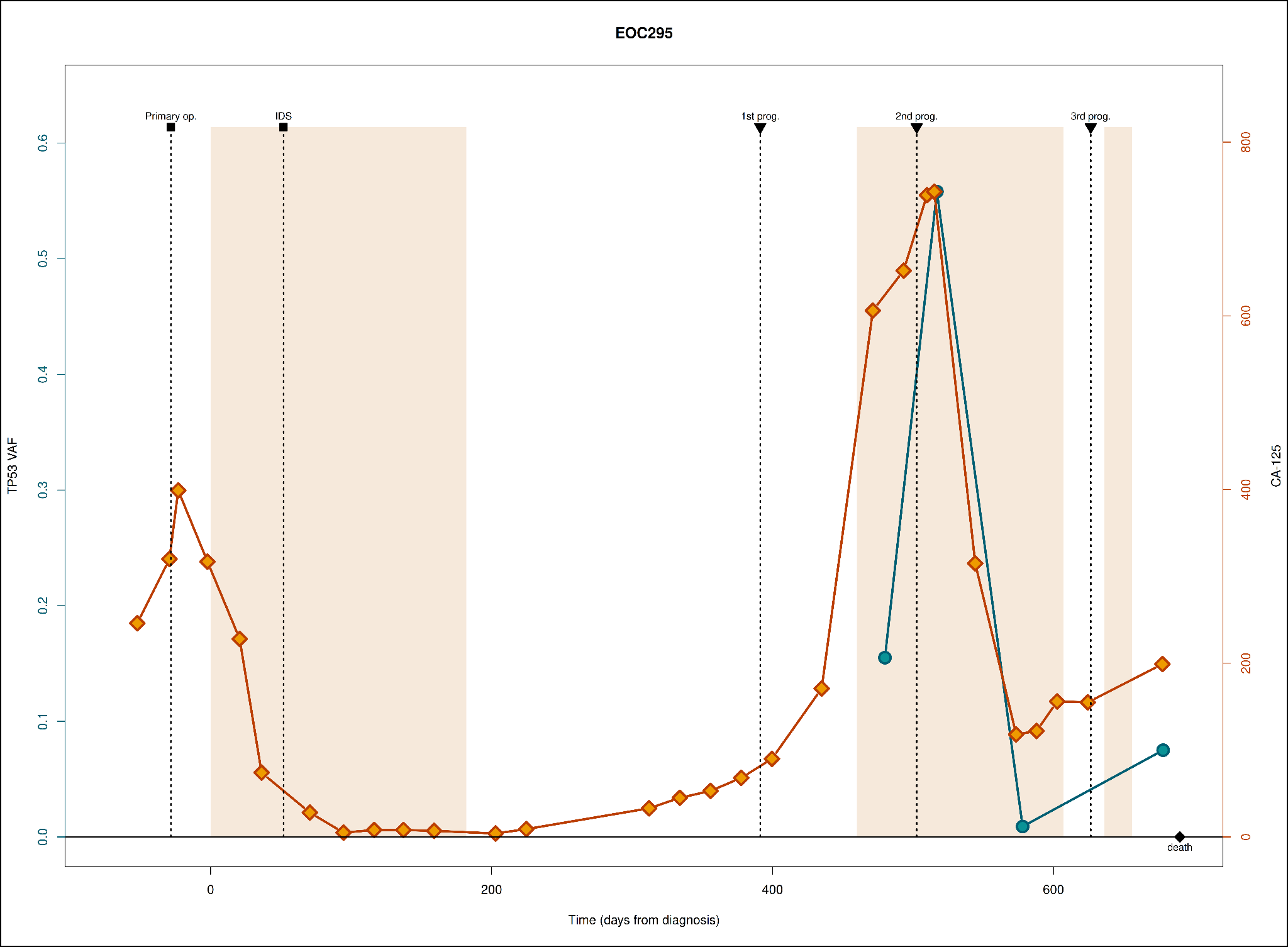 |
| 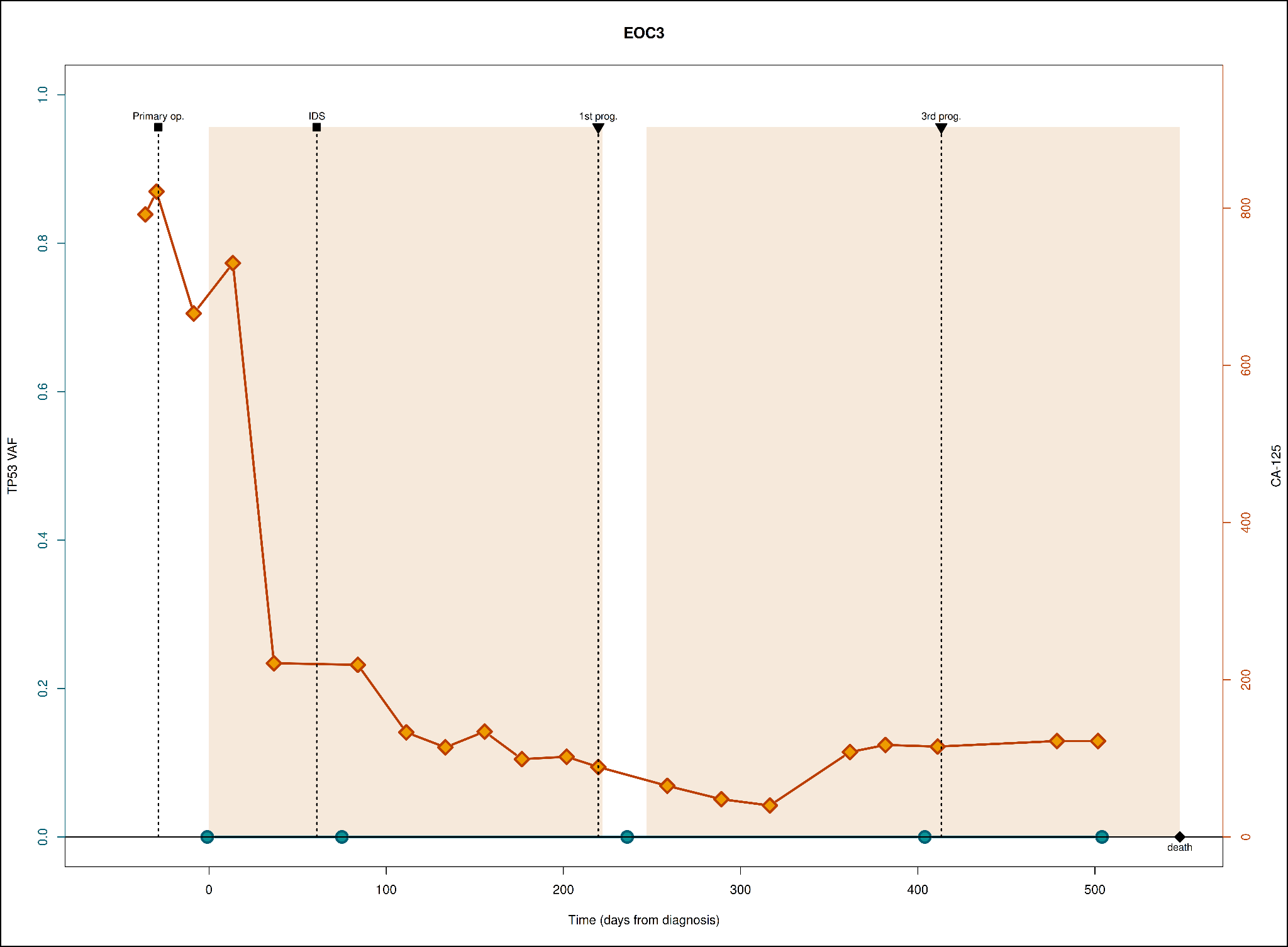 | 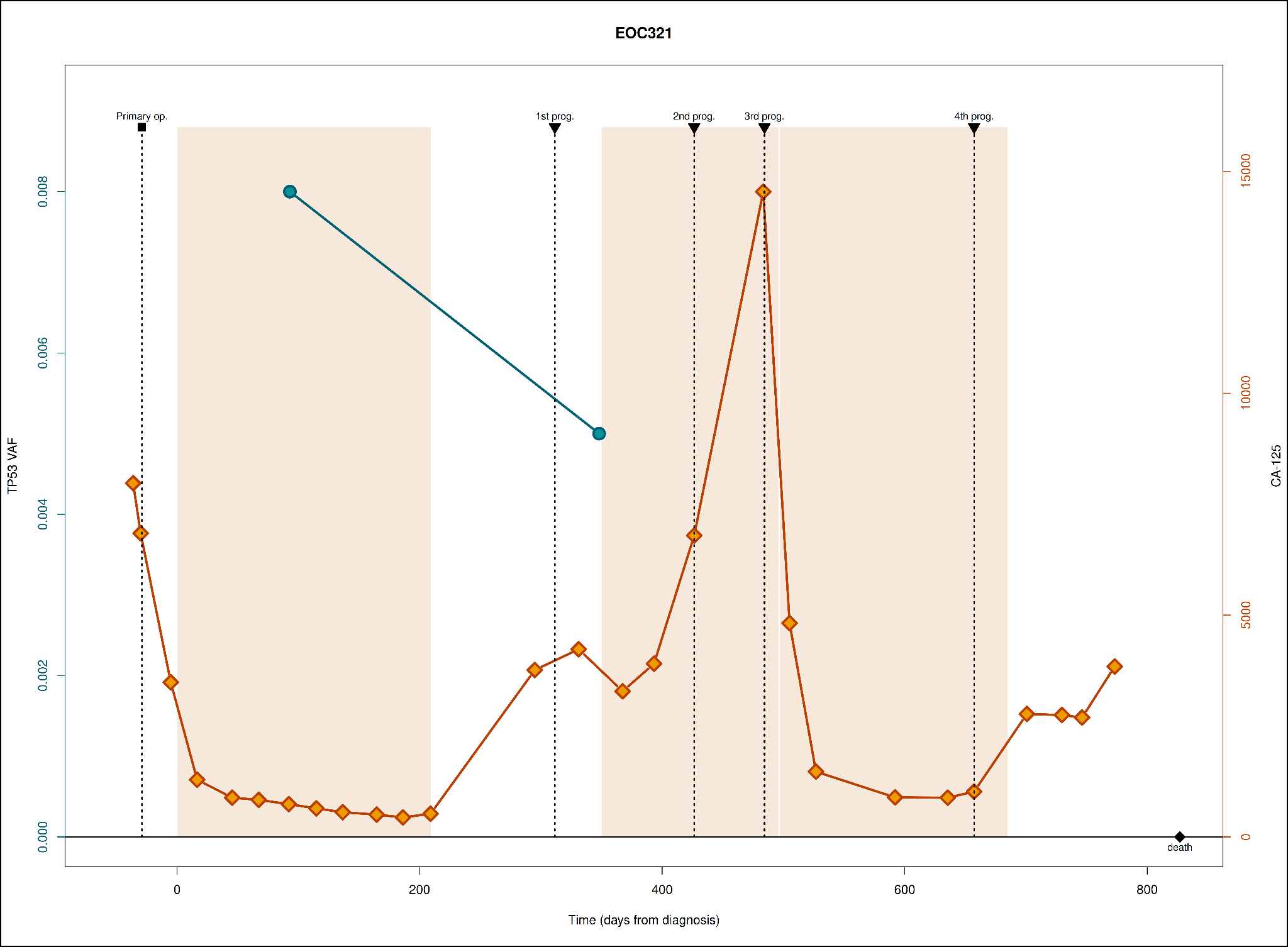 |
| 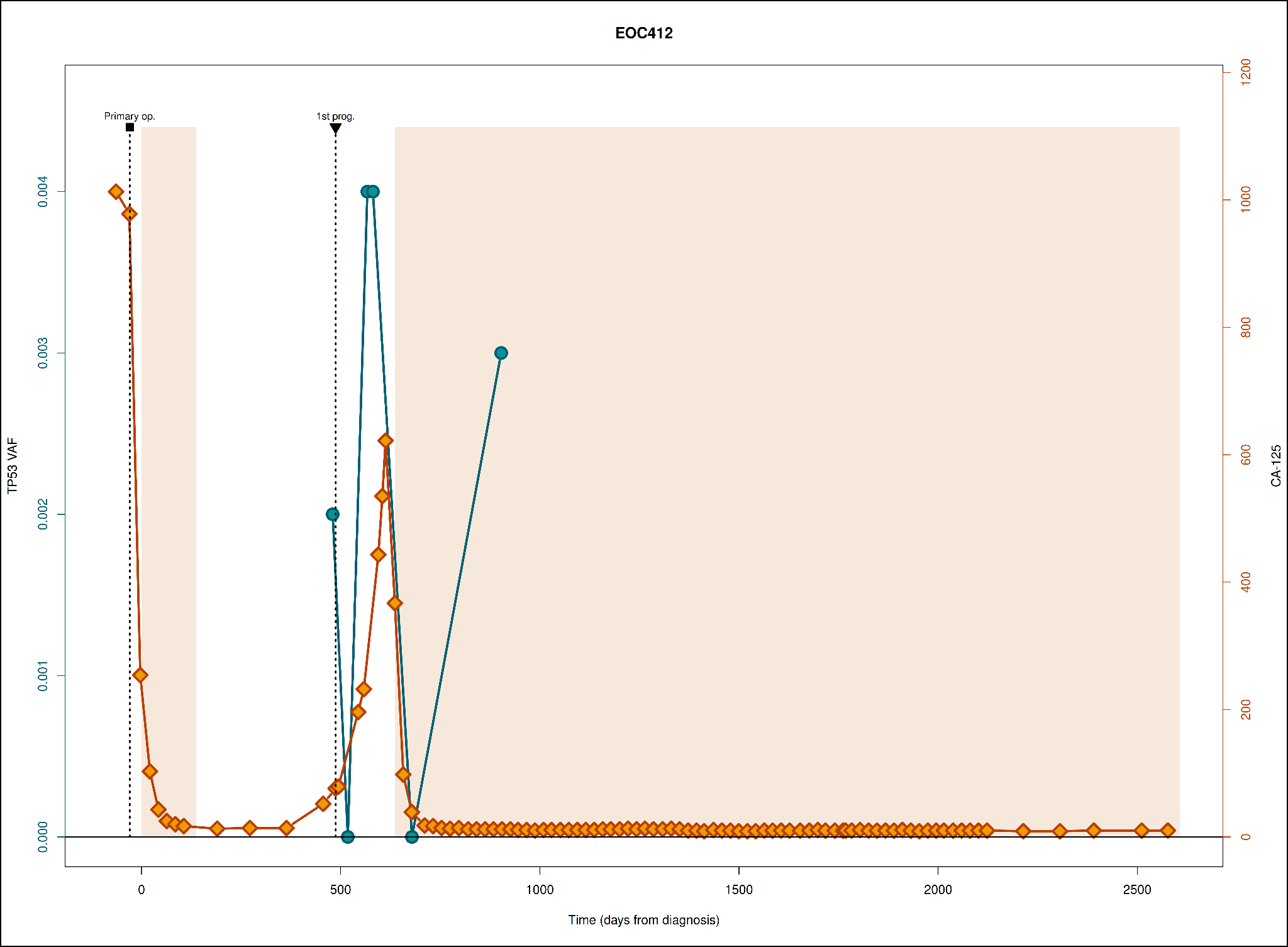 | 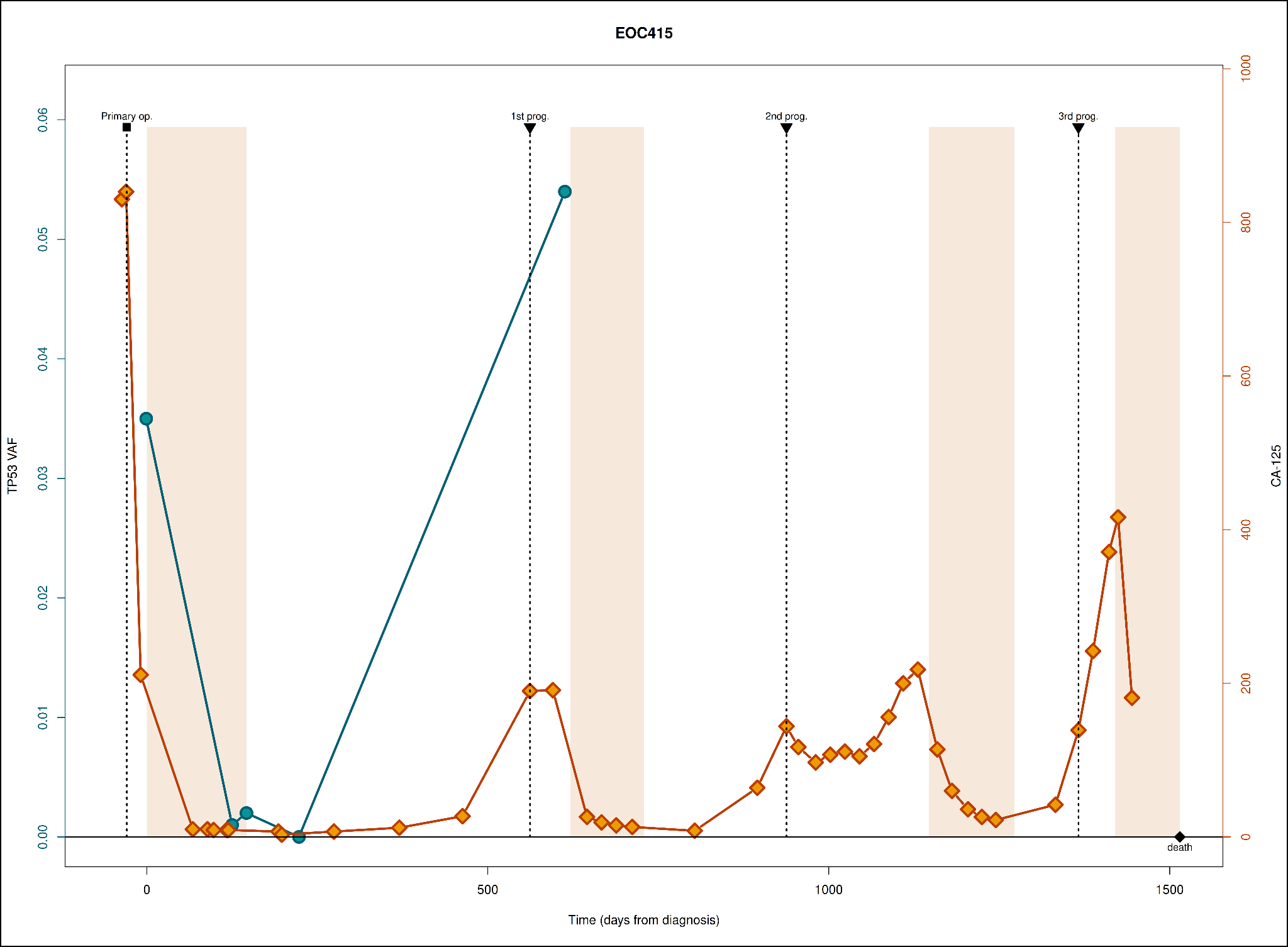 |
| 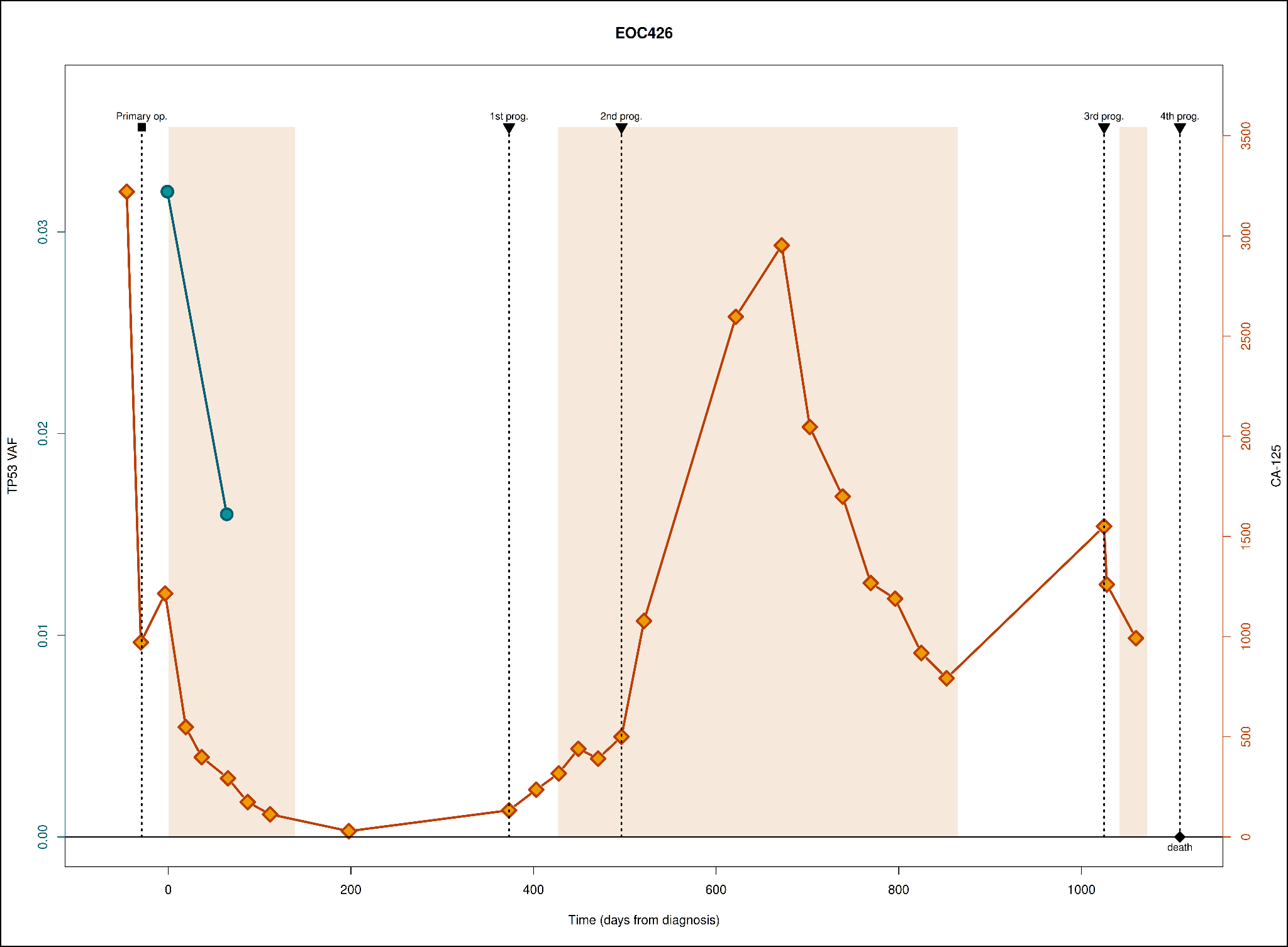 | 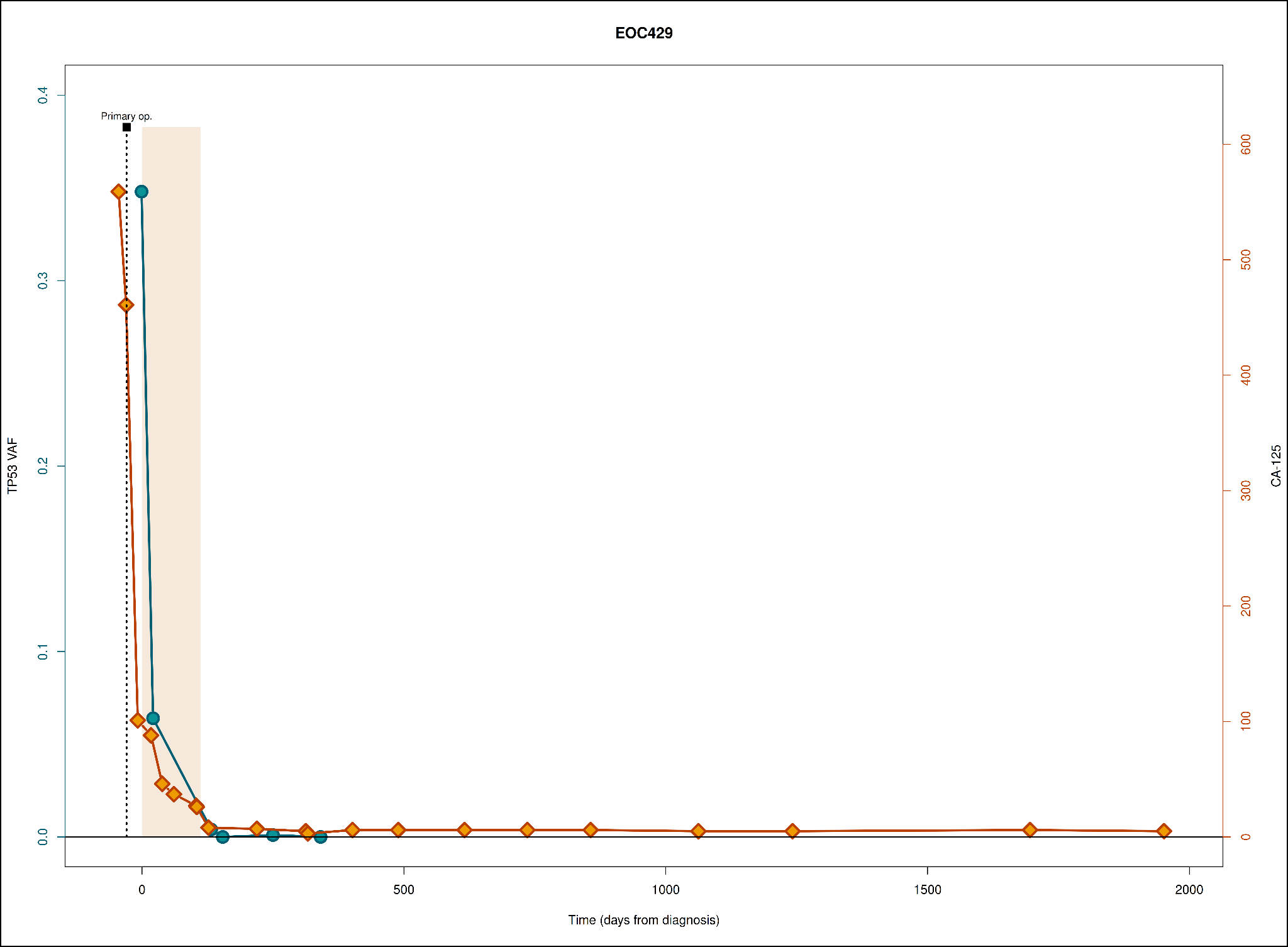 |
| 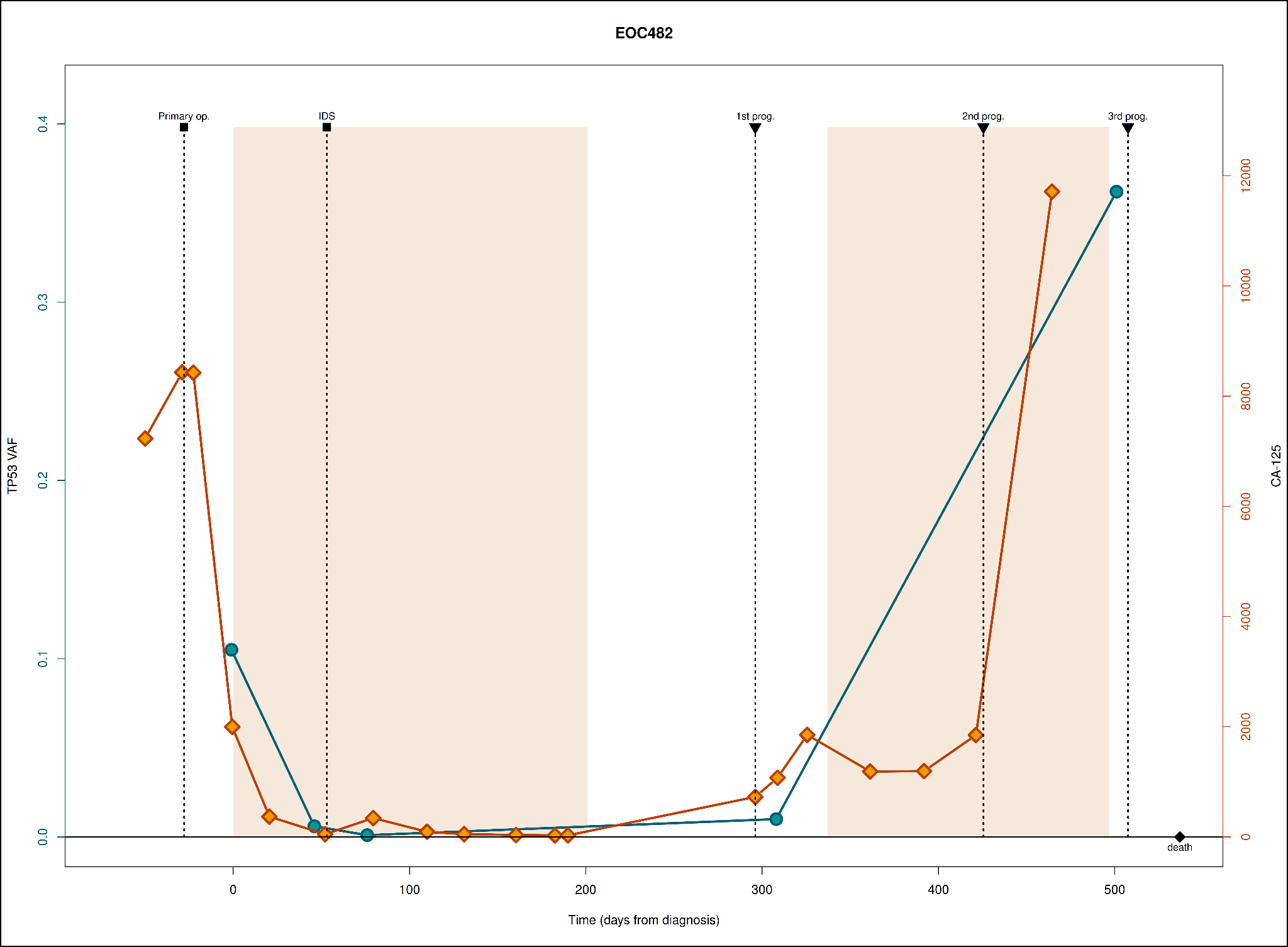 | 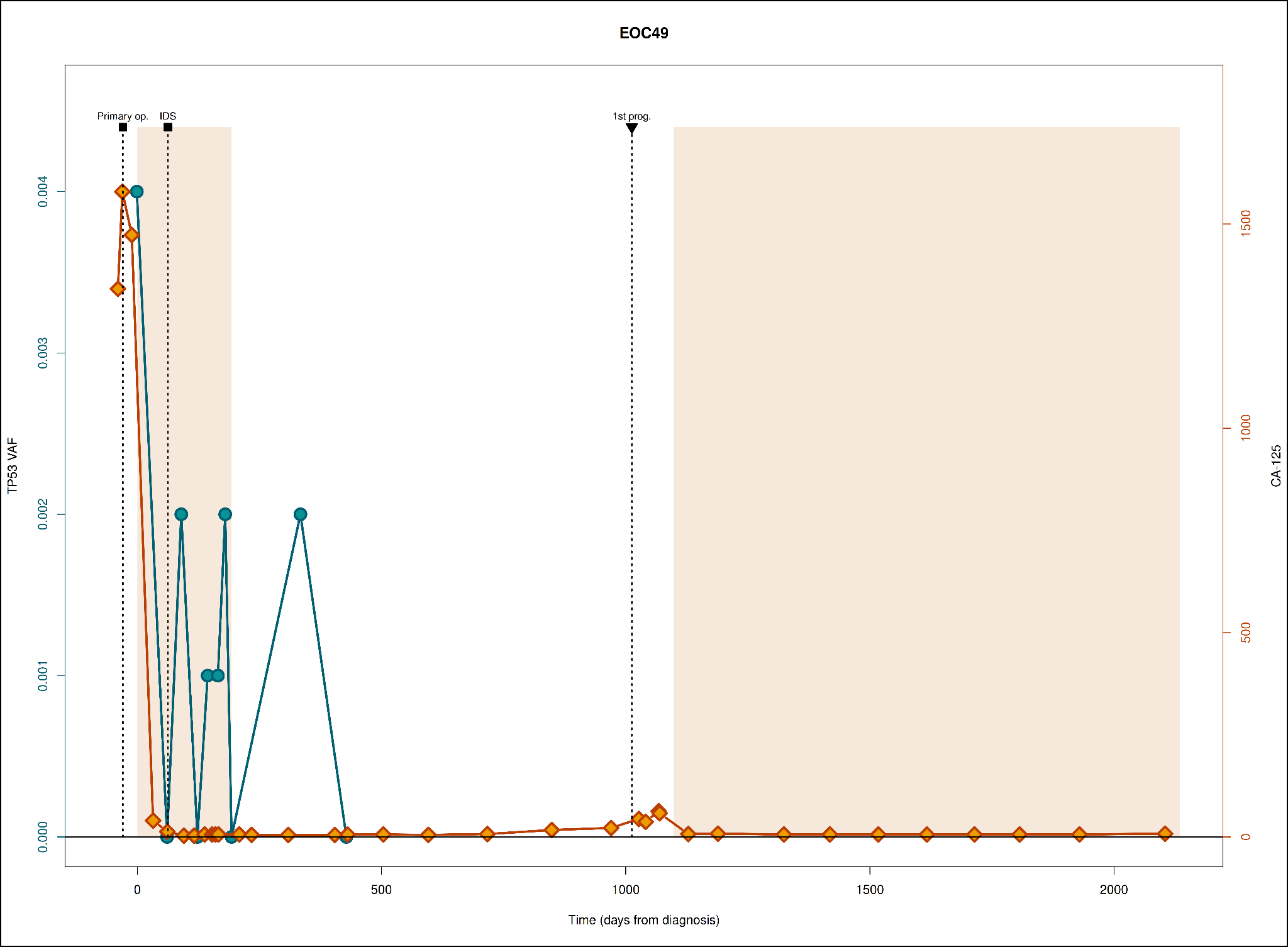 |
| 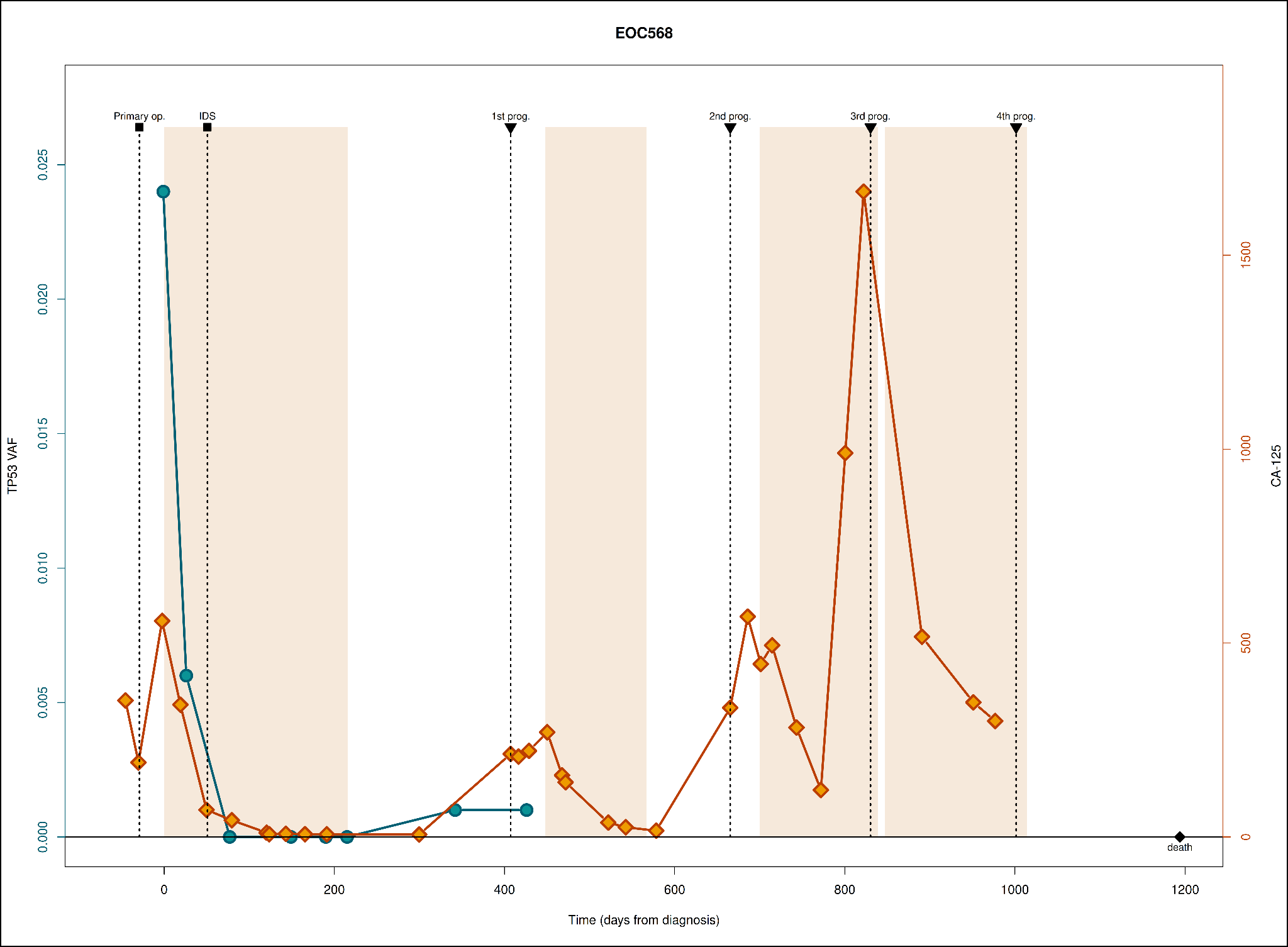 | 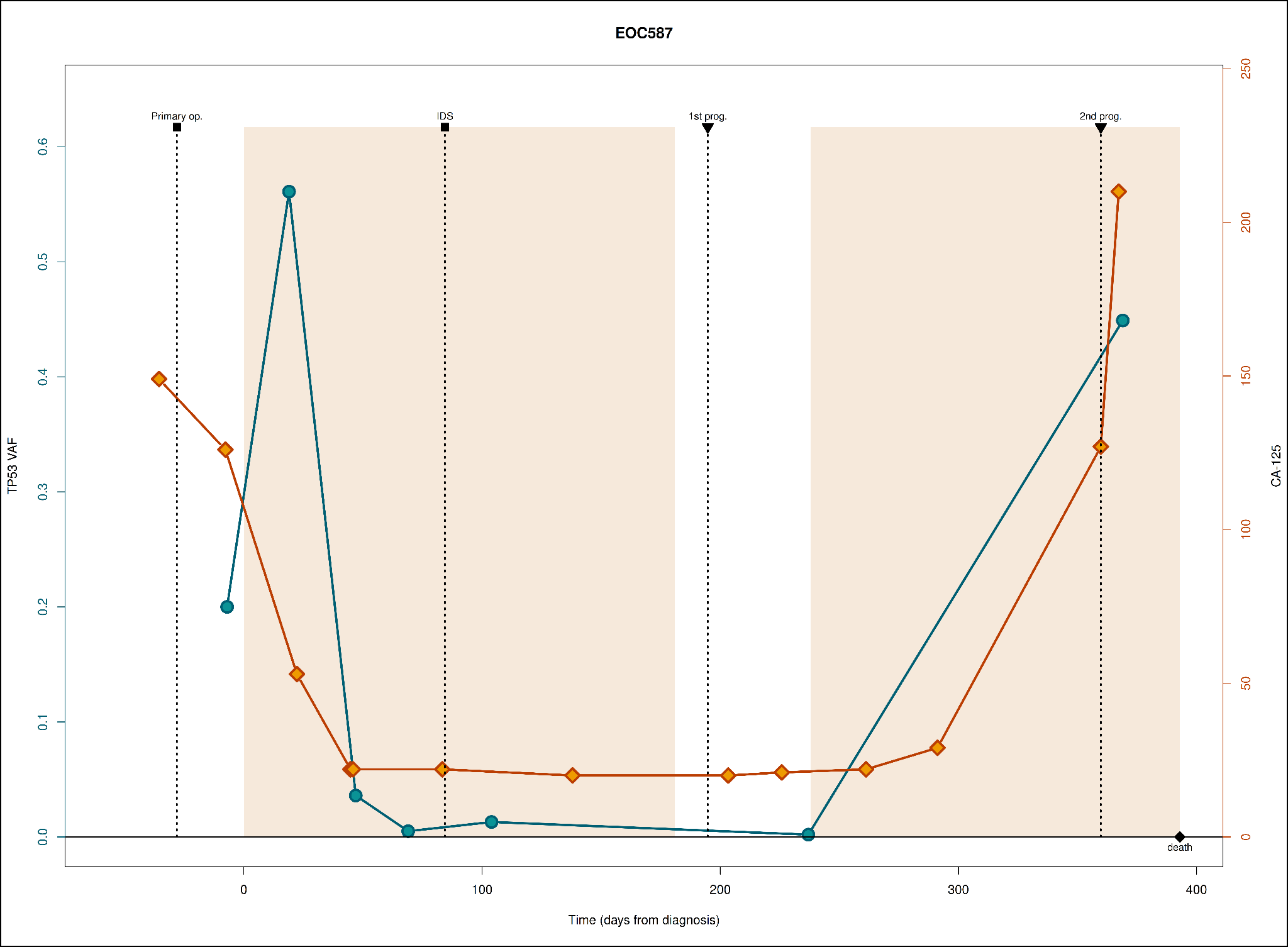 |
| 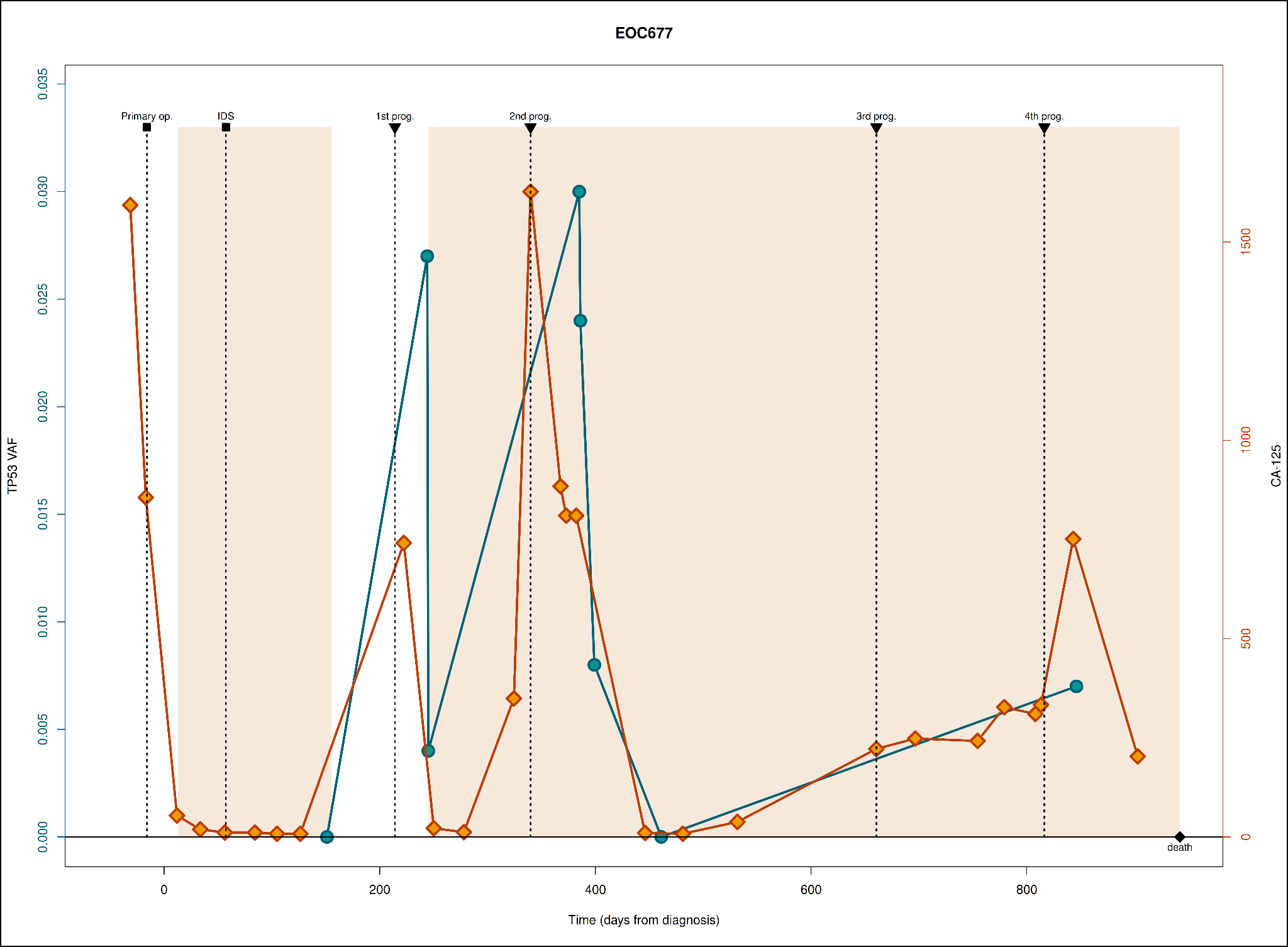 | 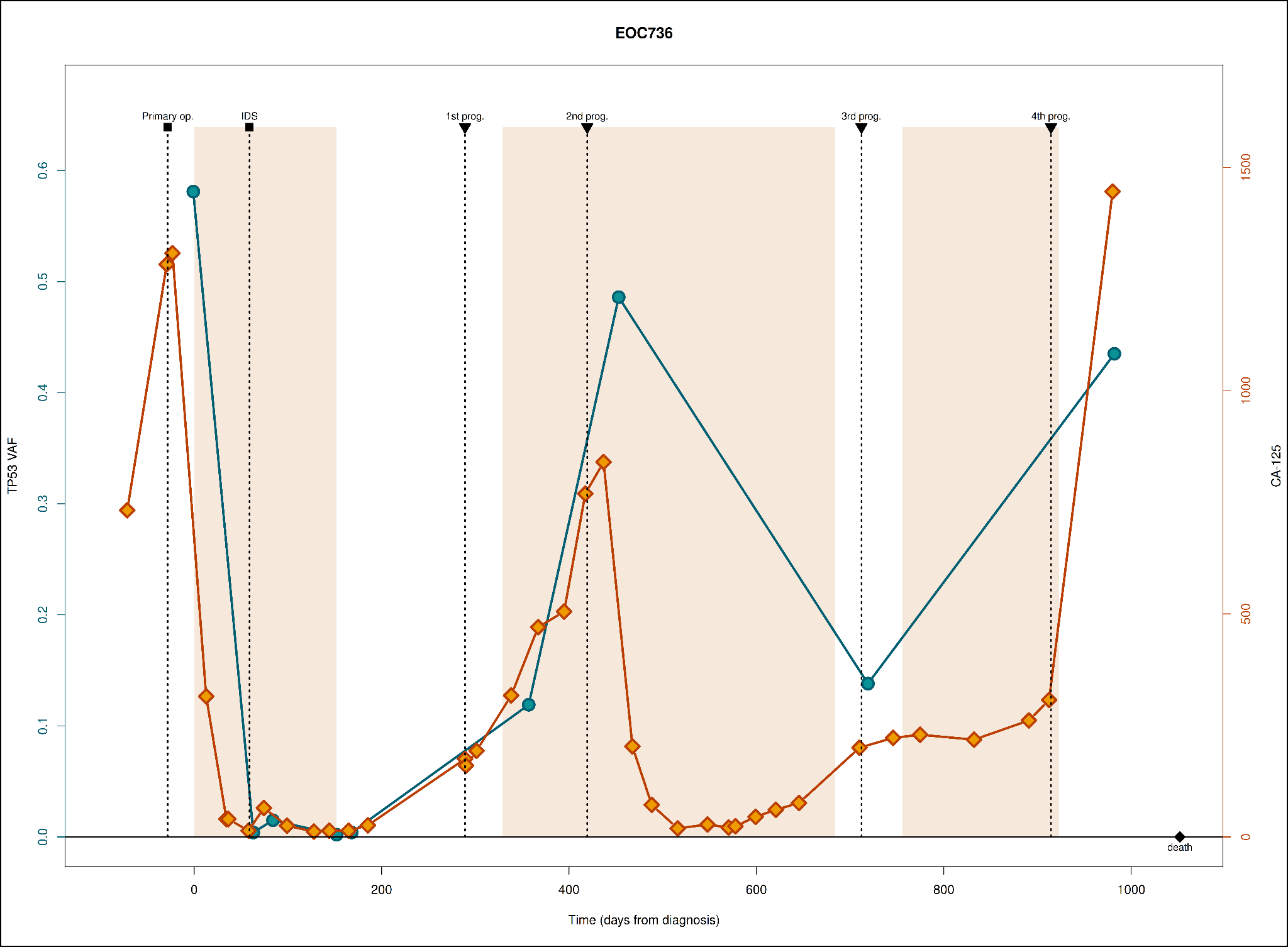 |
| 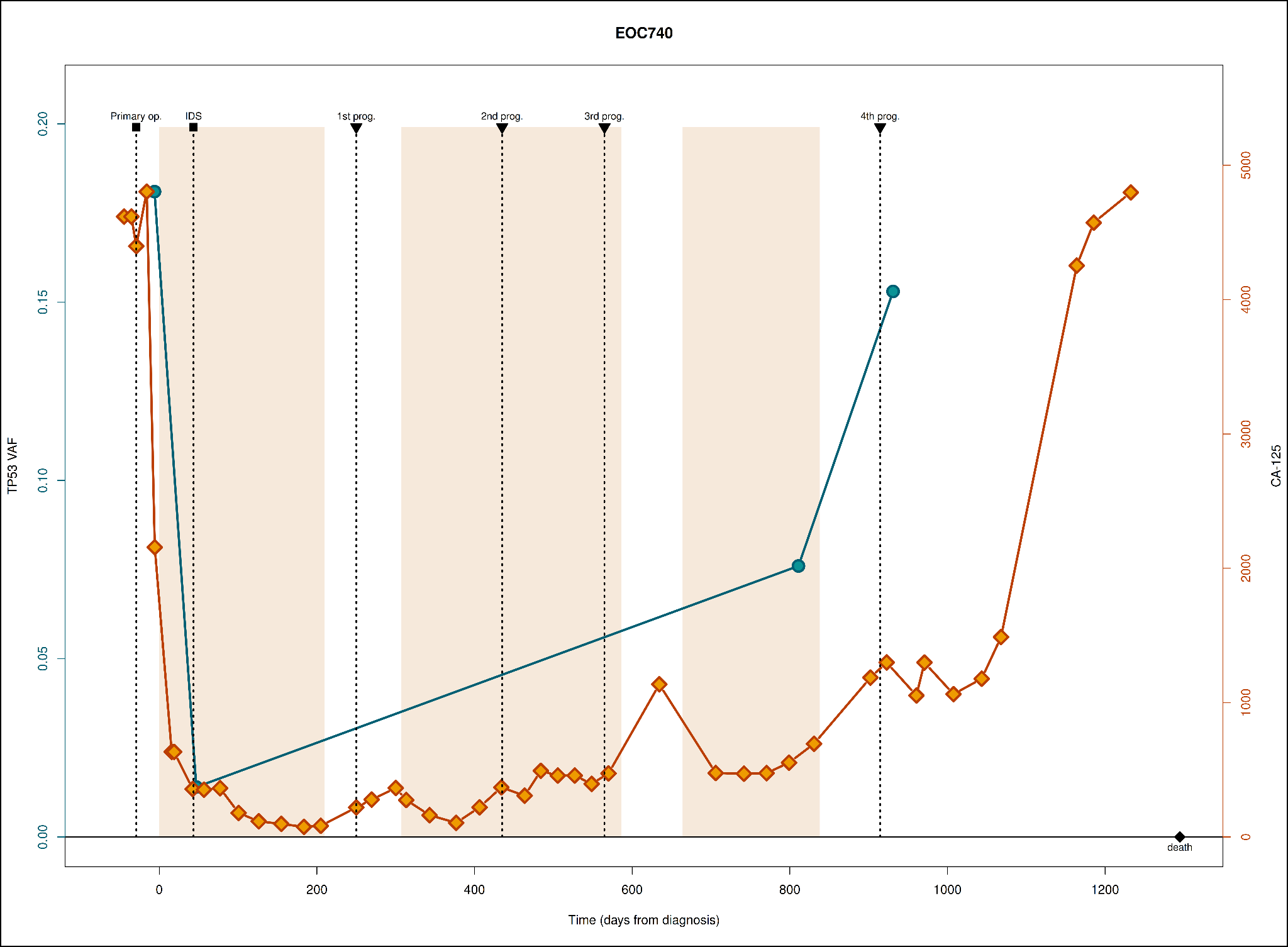 | 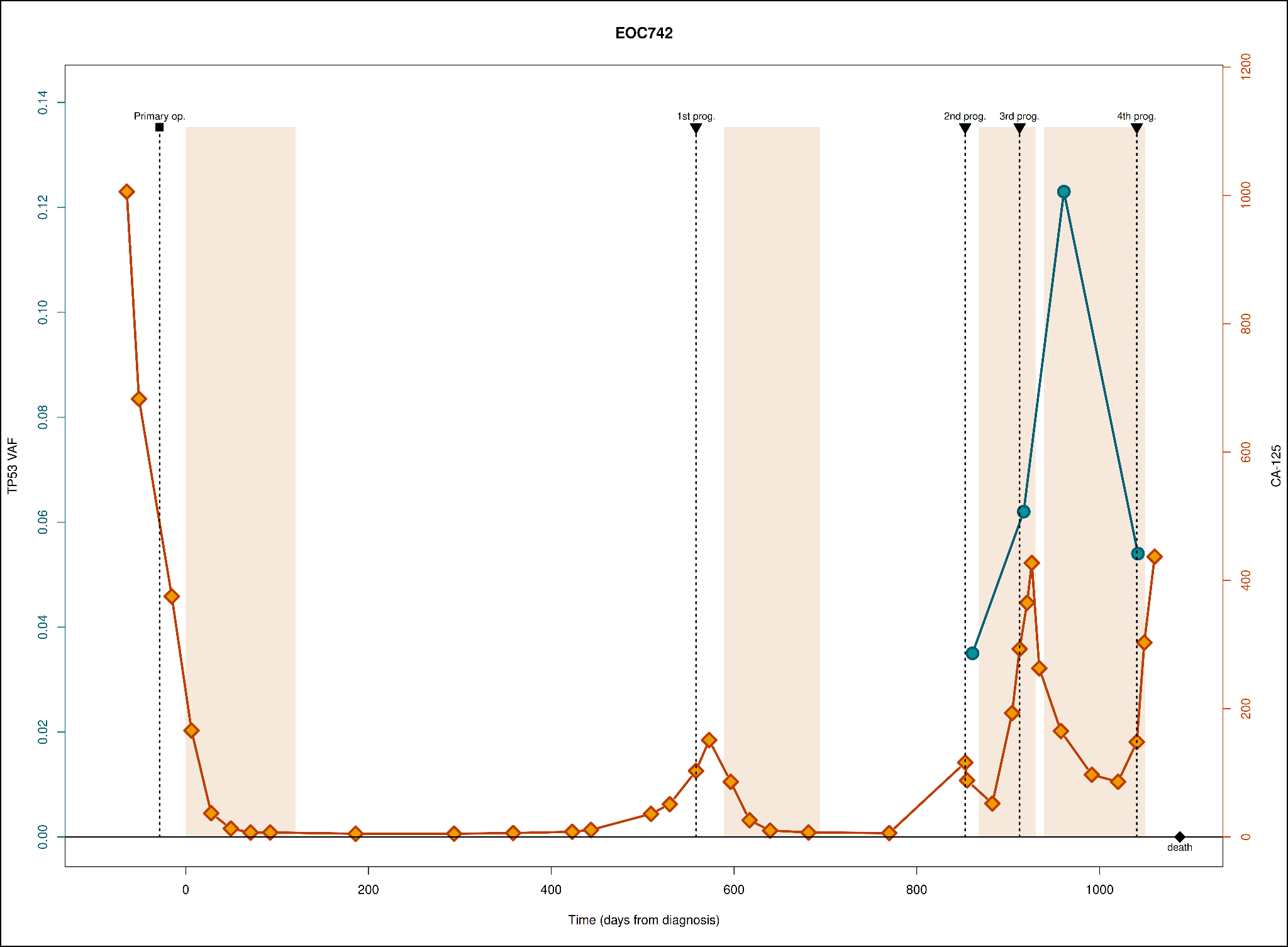 |
| 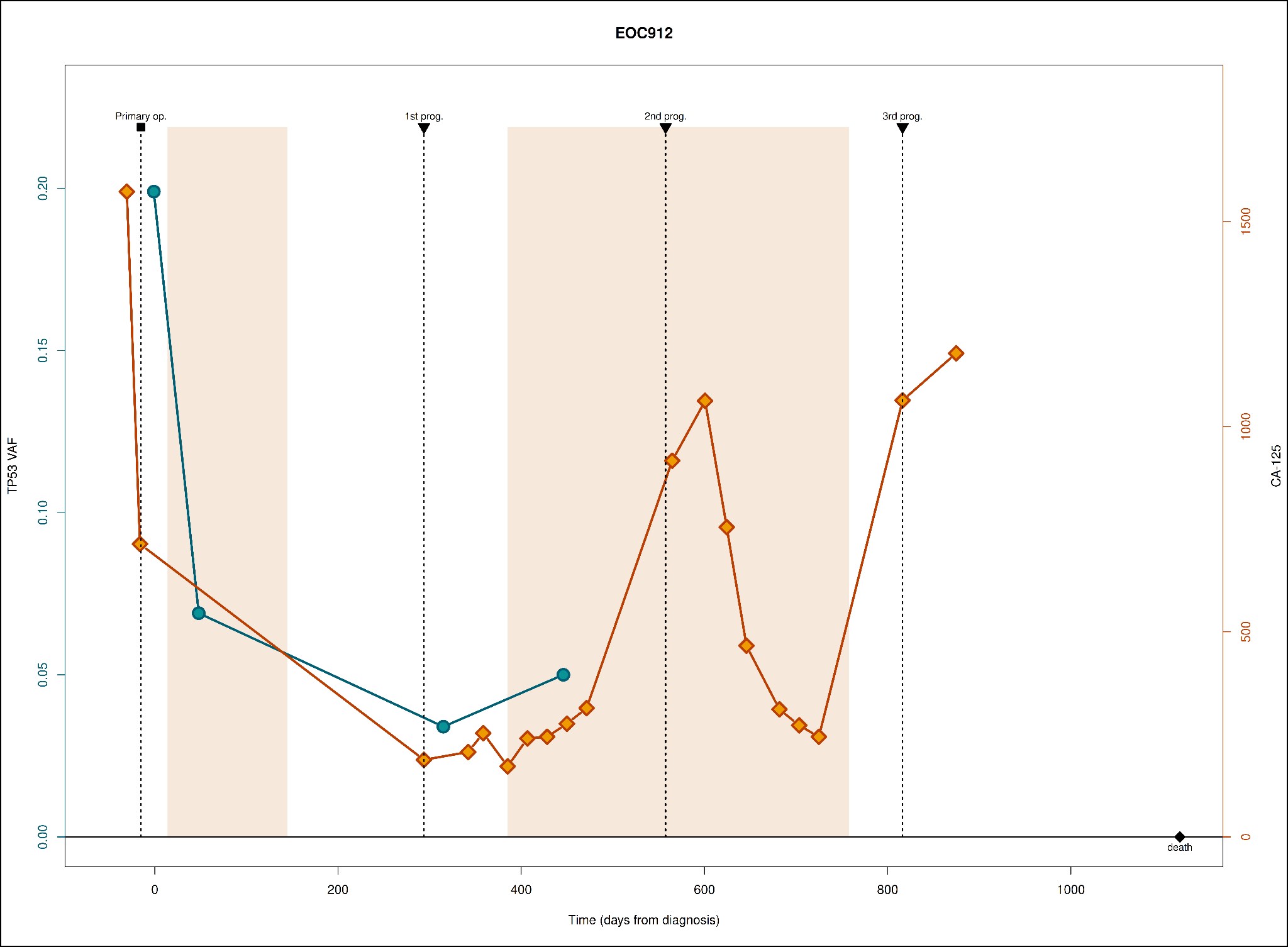 | 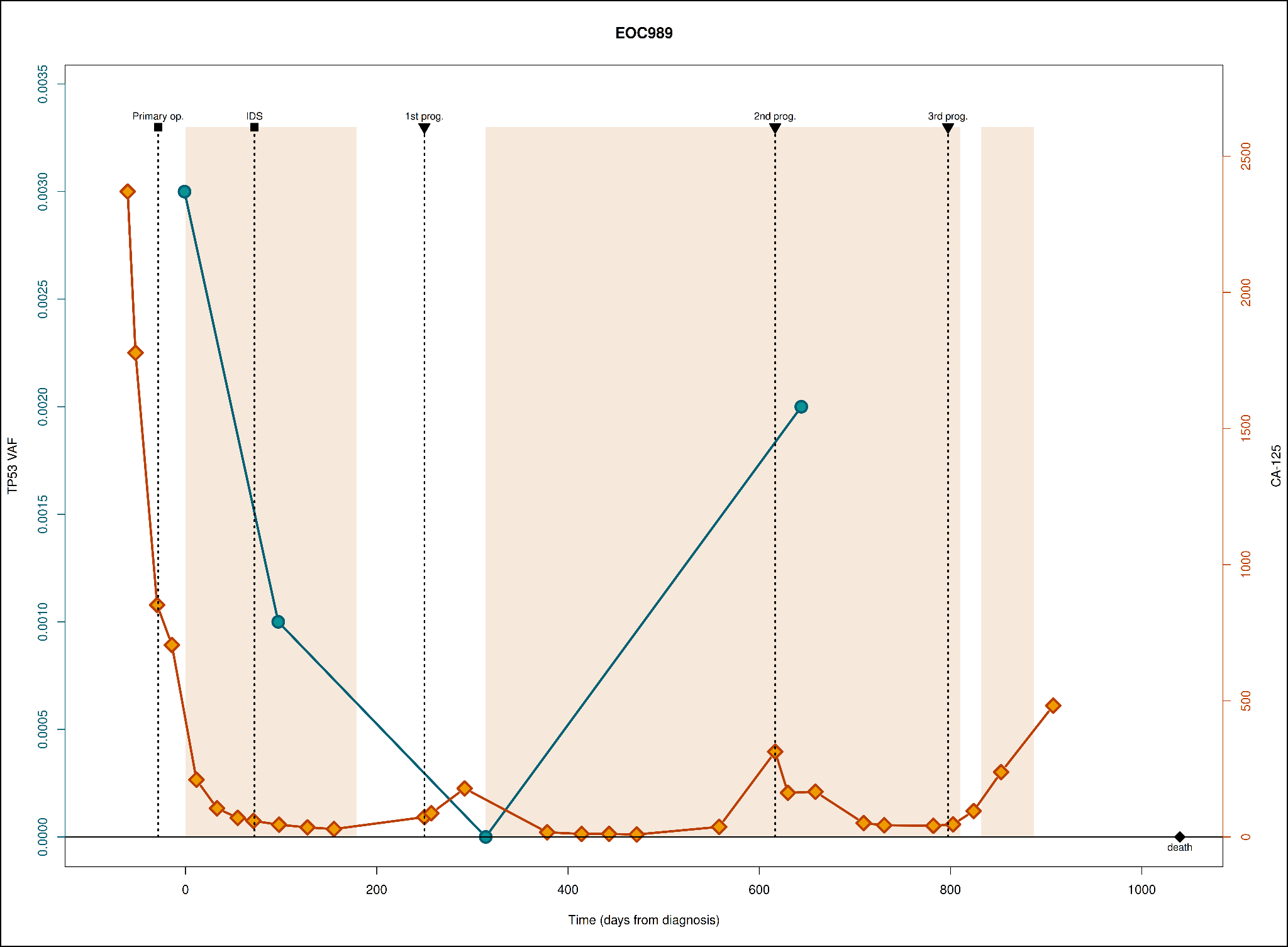 |
| 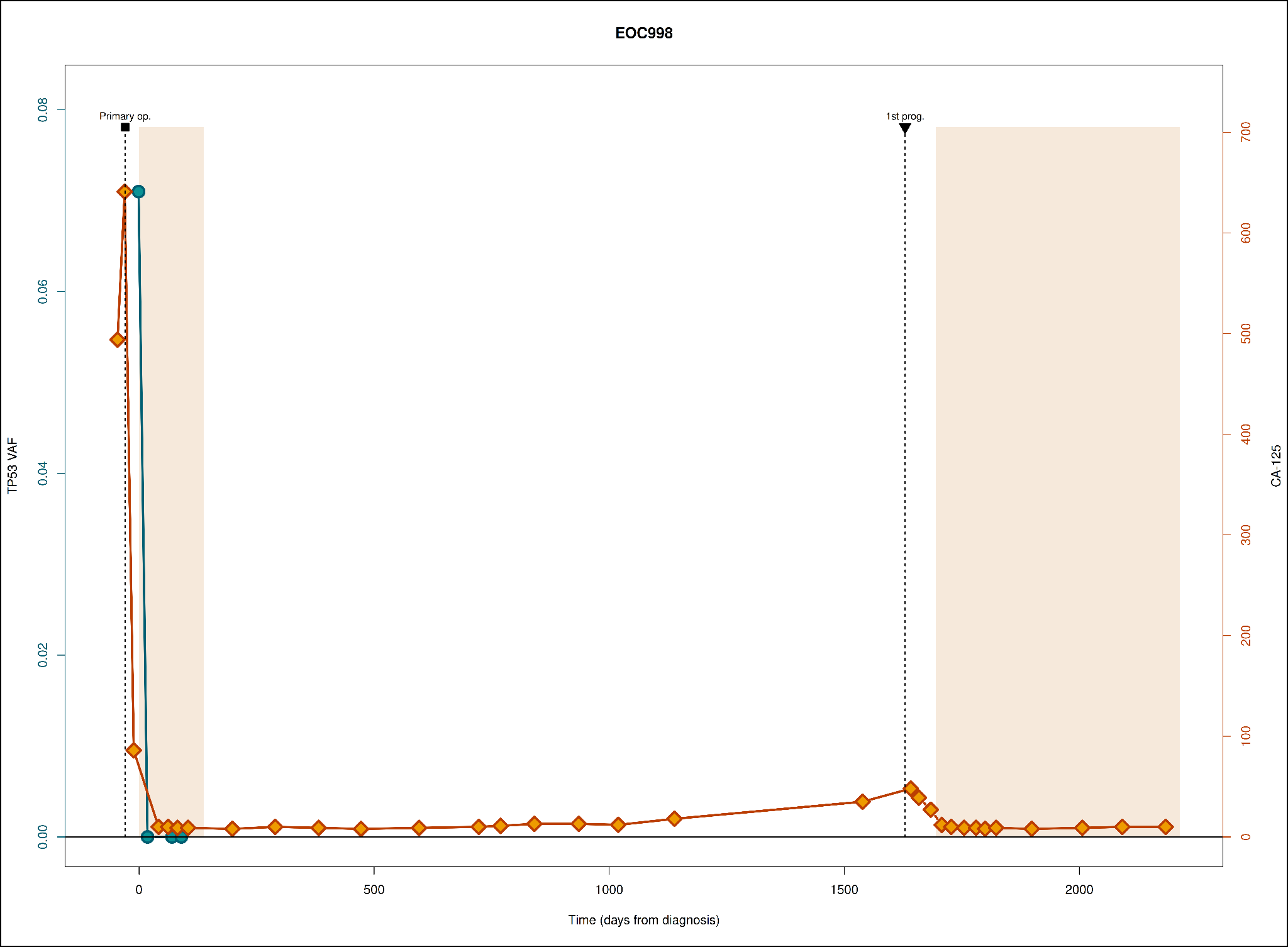 |  |

**Figure S3** Per patient mutation heatmaps. Tumor content as *TP53* VAF can be seen at the top of the heatmaps; additionally, columns are annotated with sample type (plasma or tissue sample) and treatment phase information, while rows for CADD score [15].

| 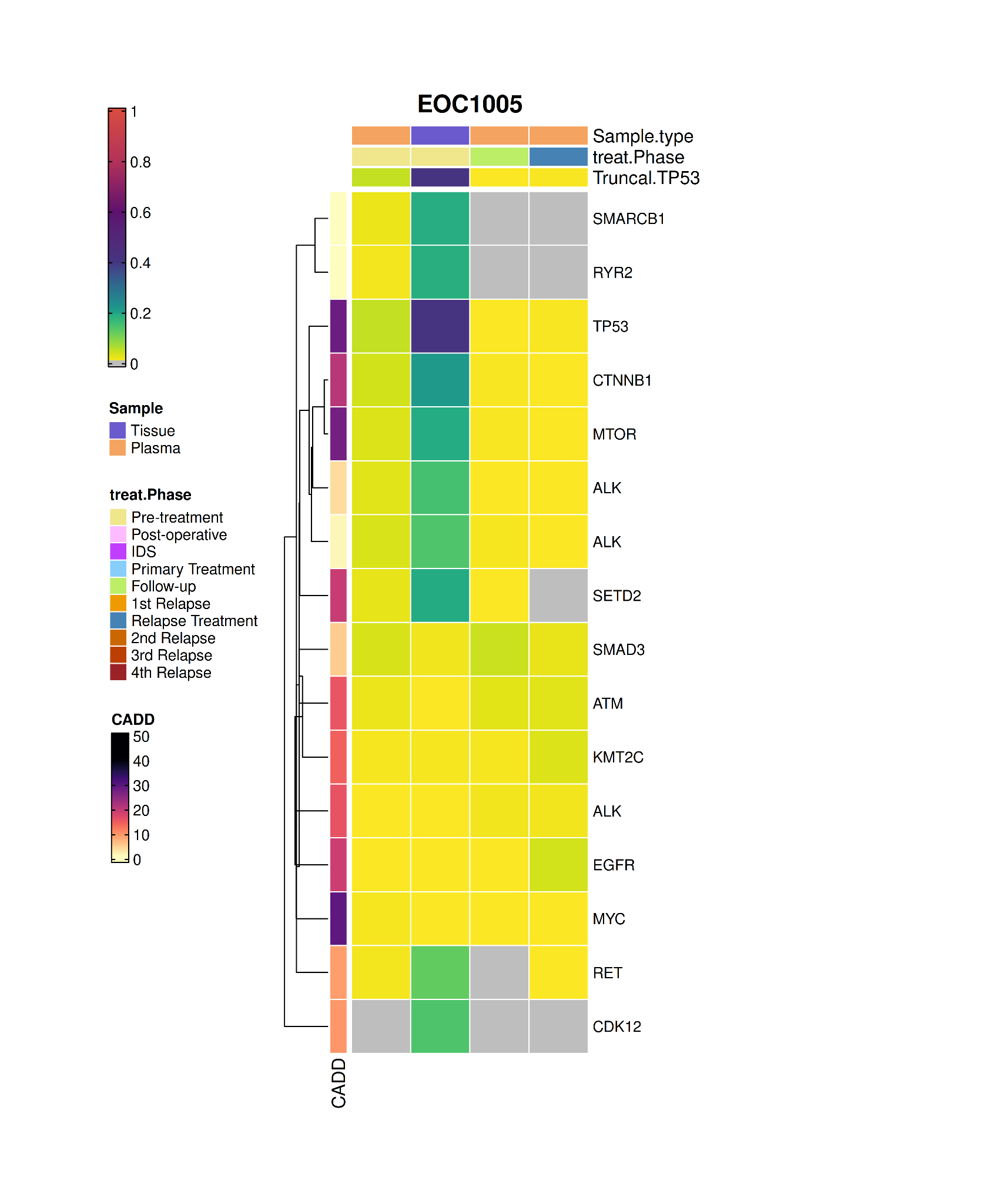 | 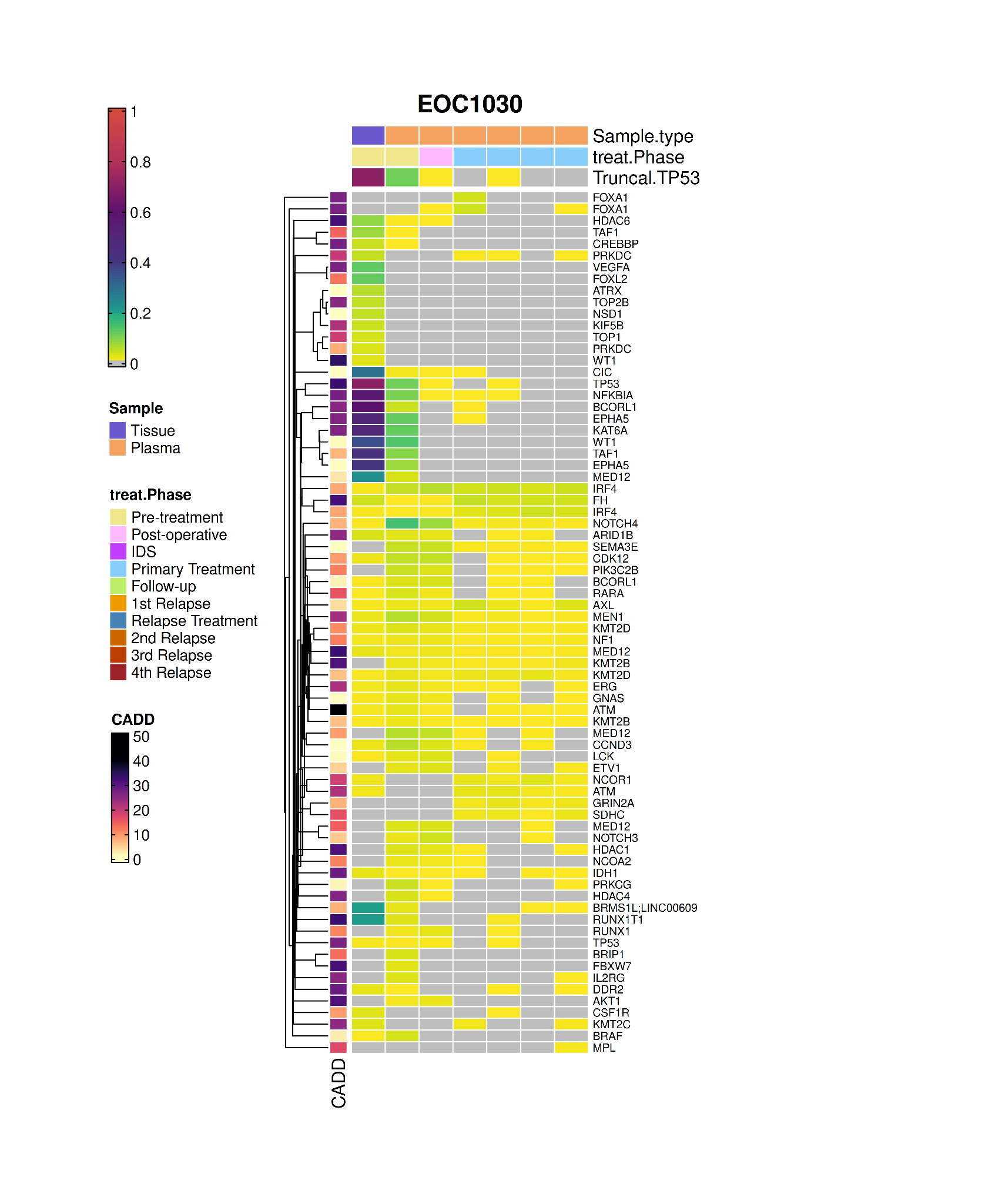 |
| --- | --- |
| 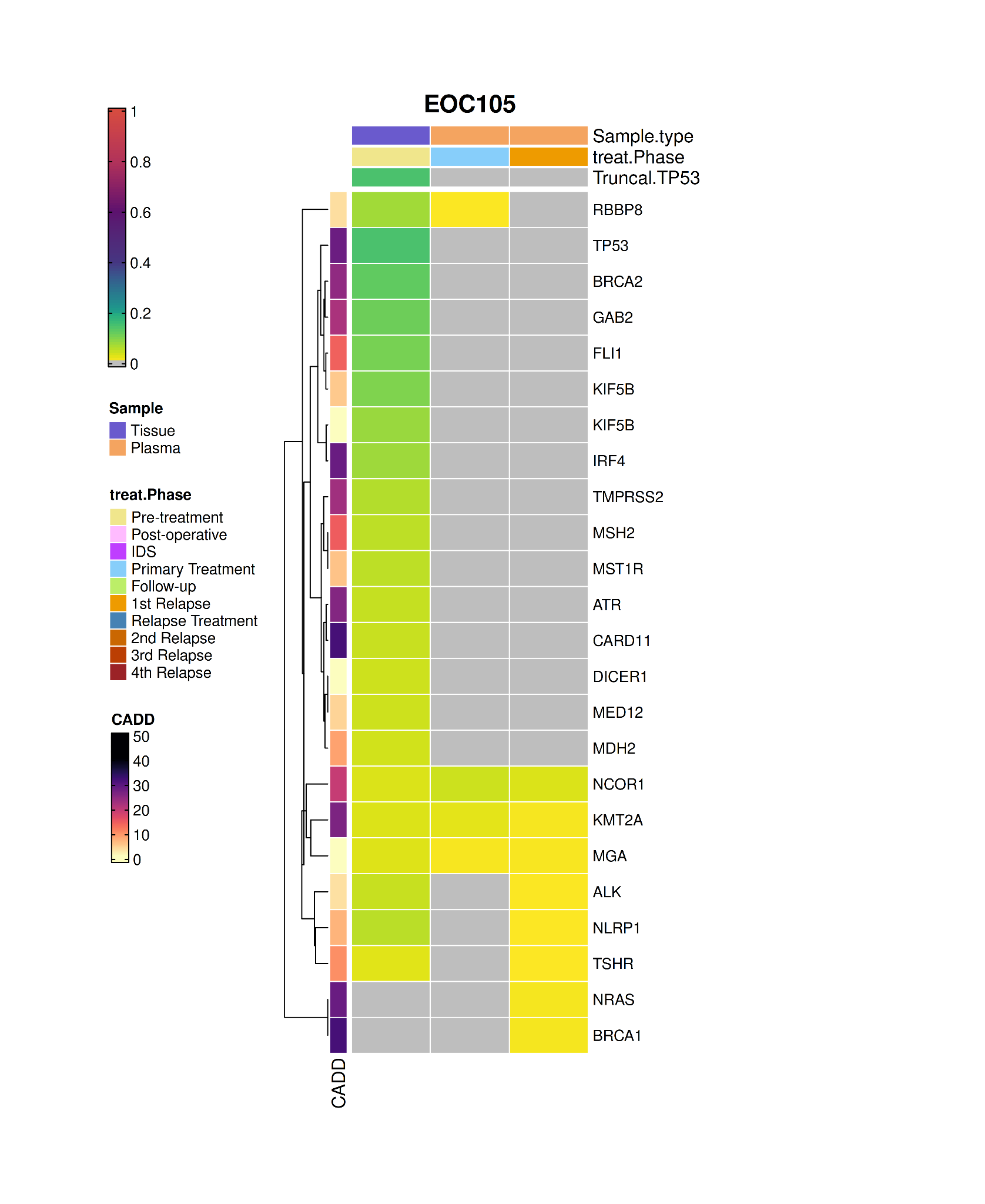 | 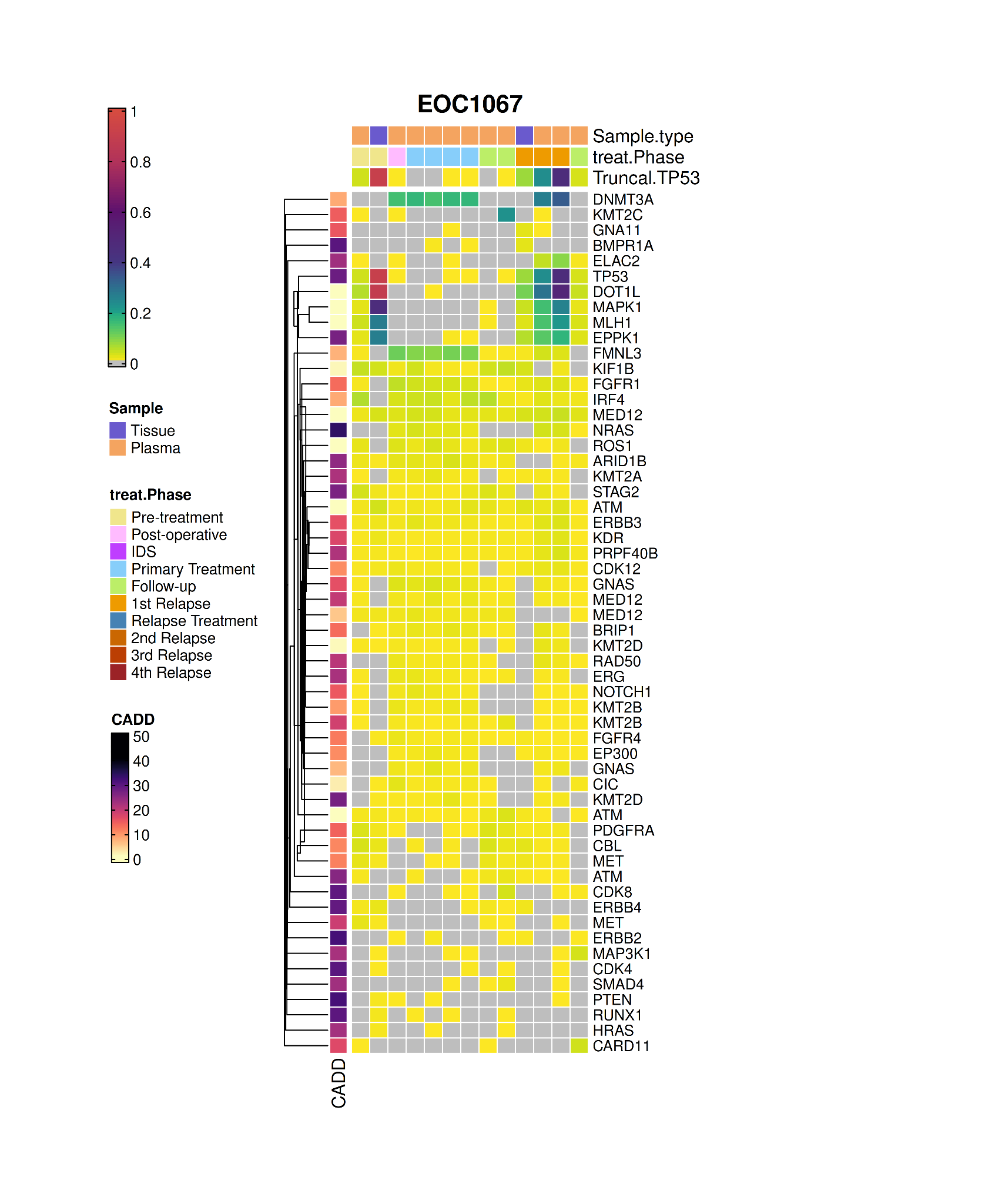 |
| 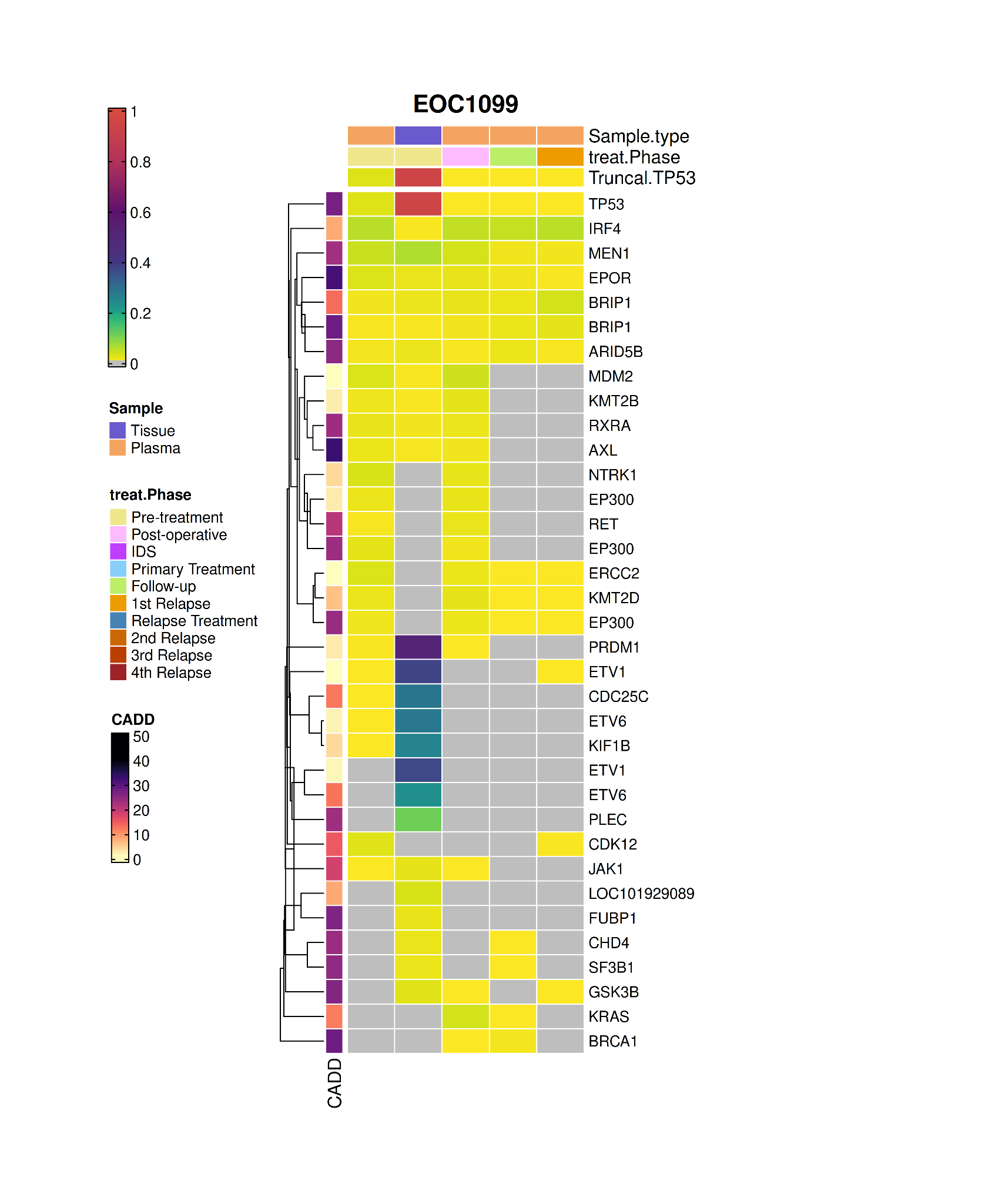 | 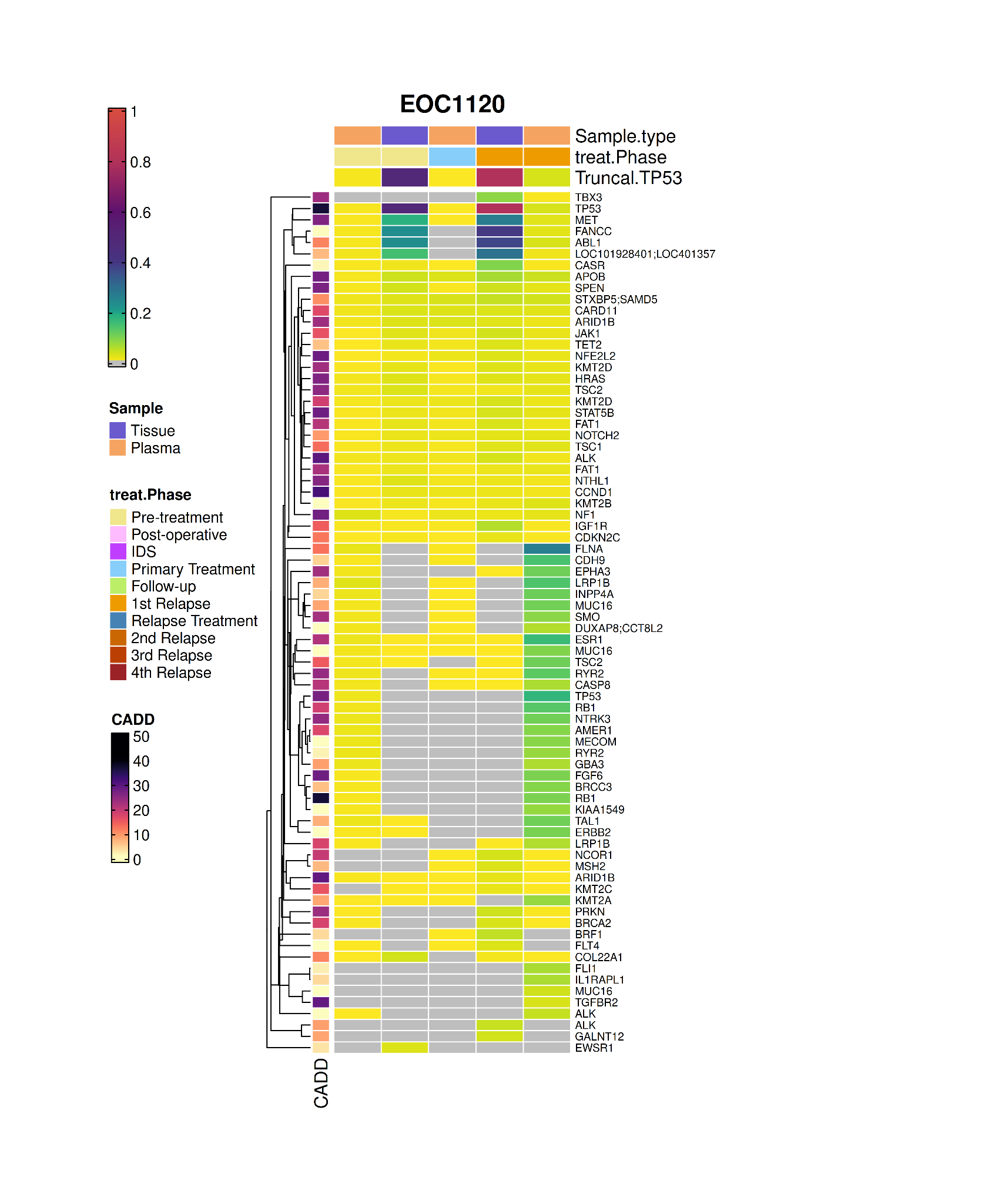 |
| 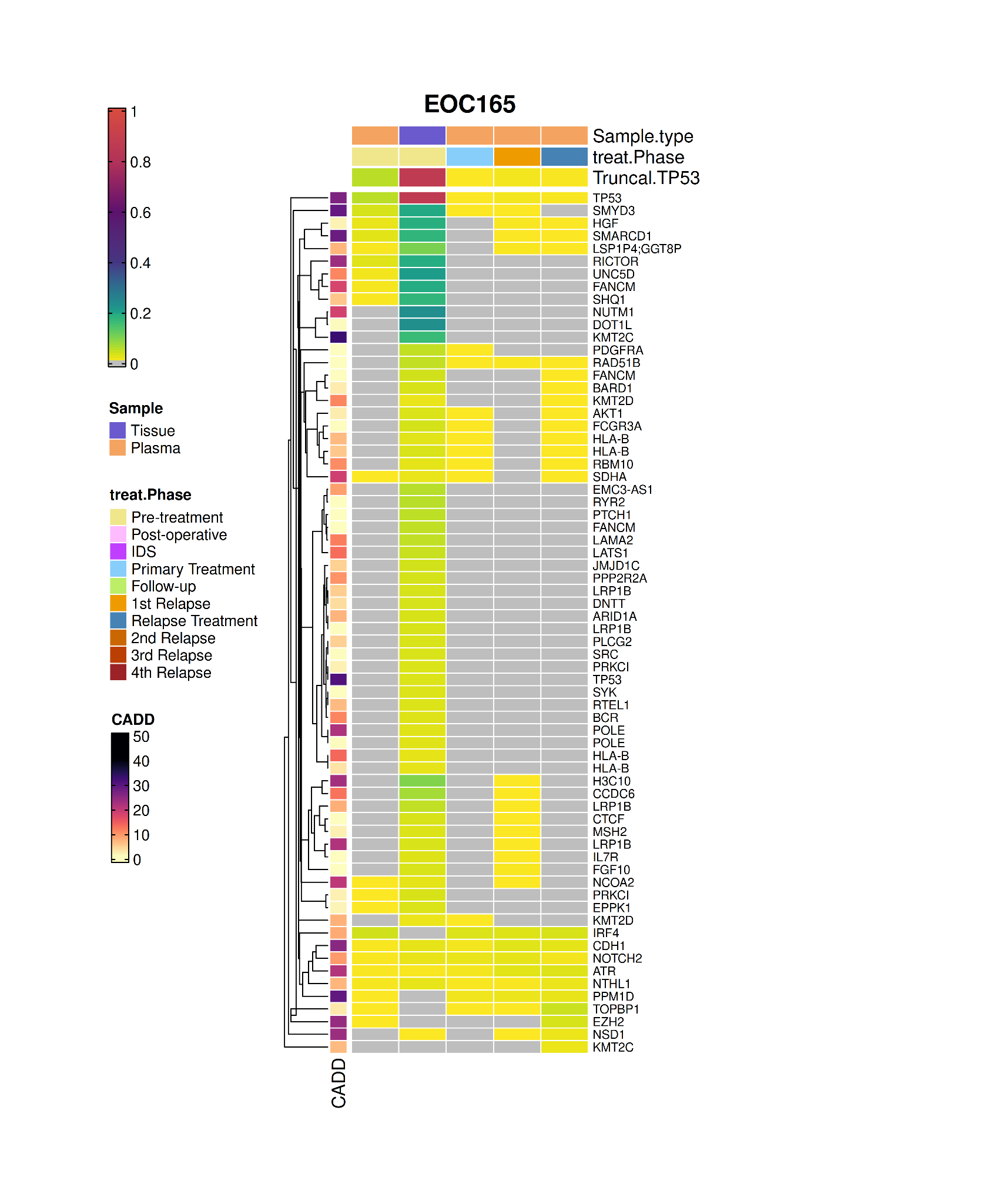 | 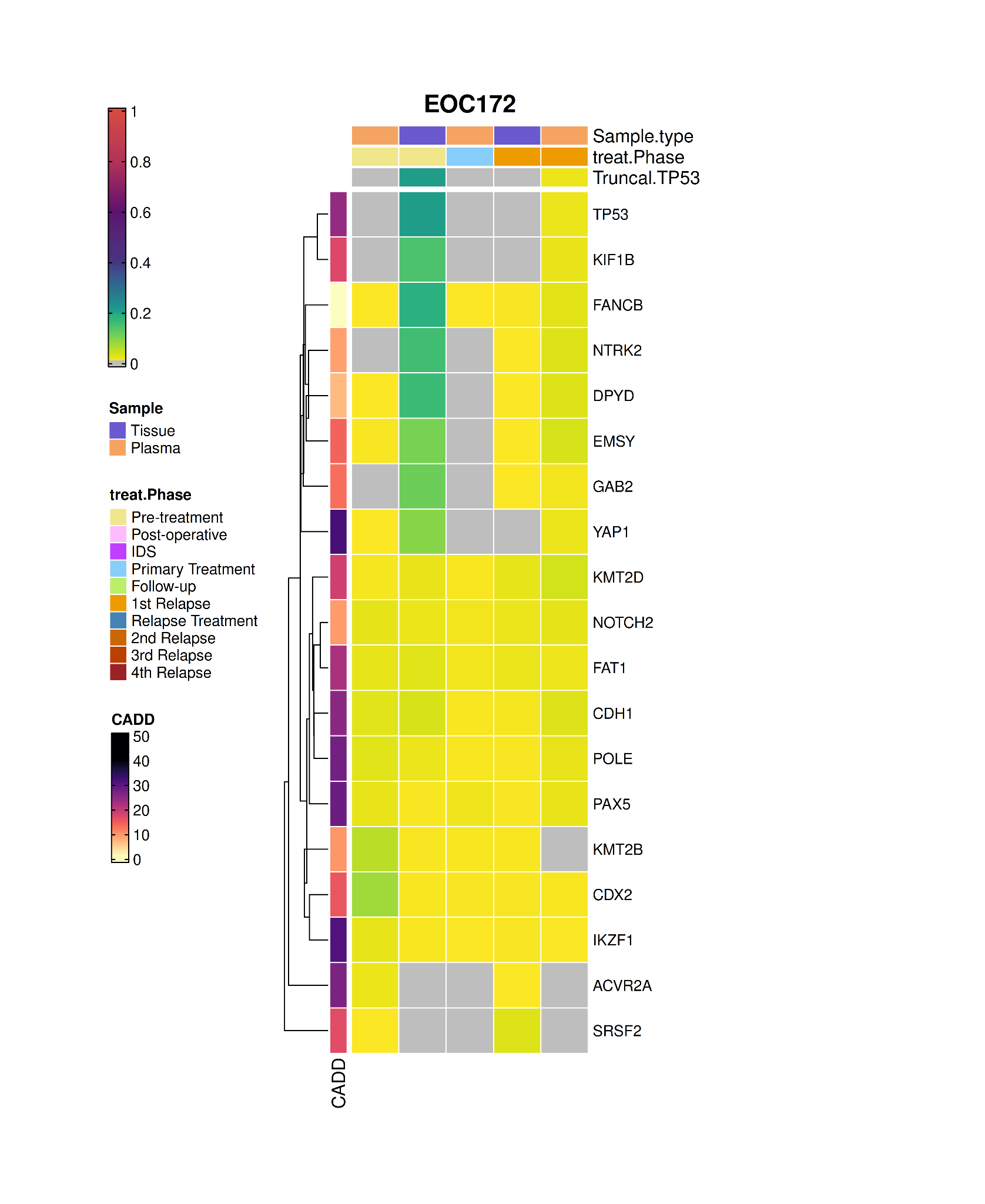 |
| 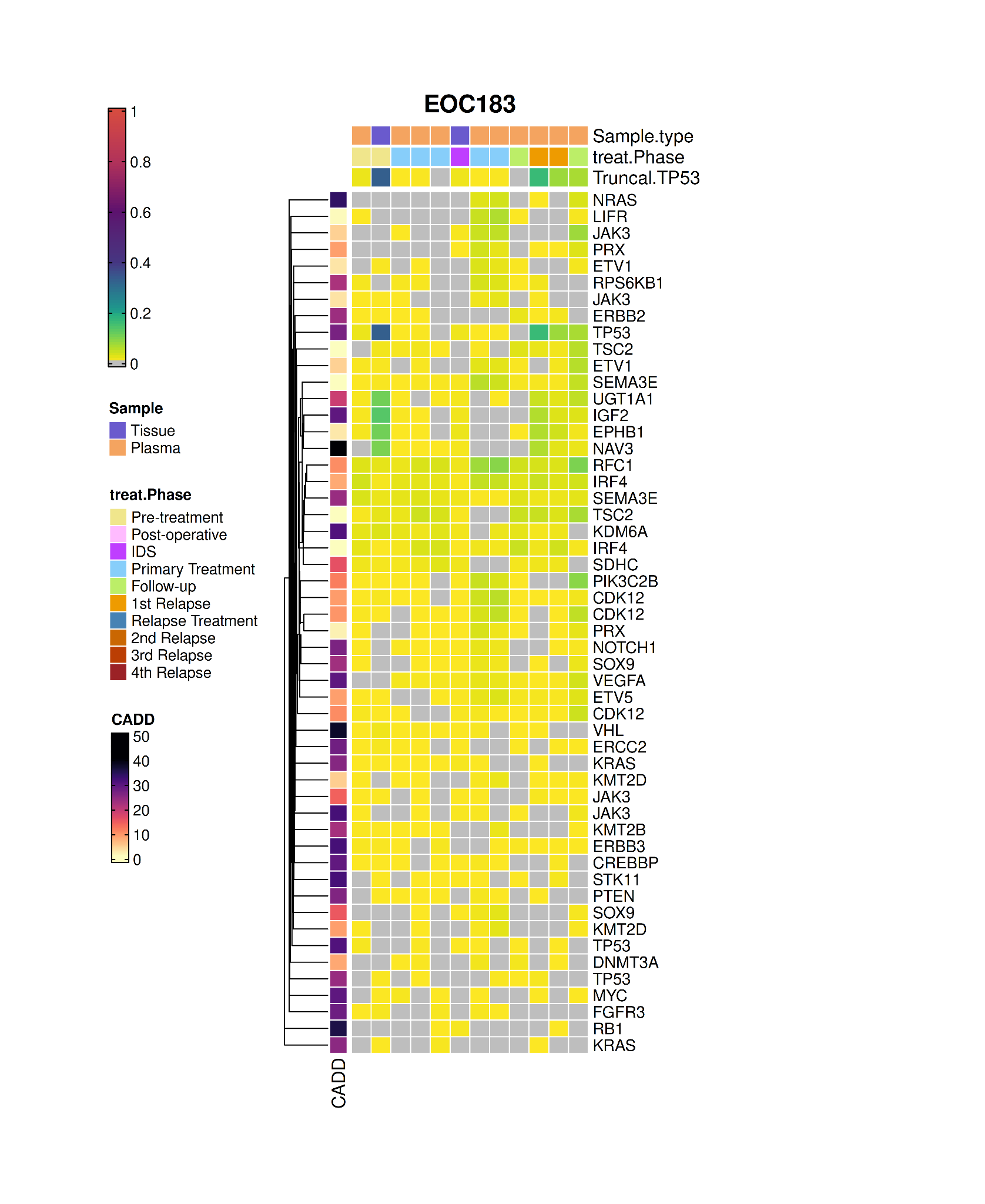 | 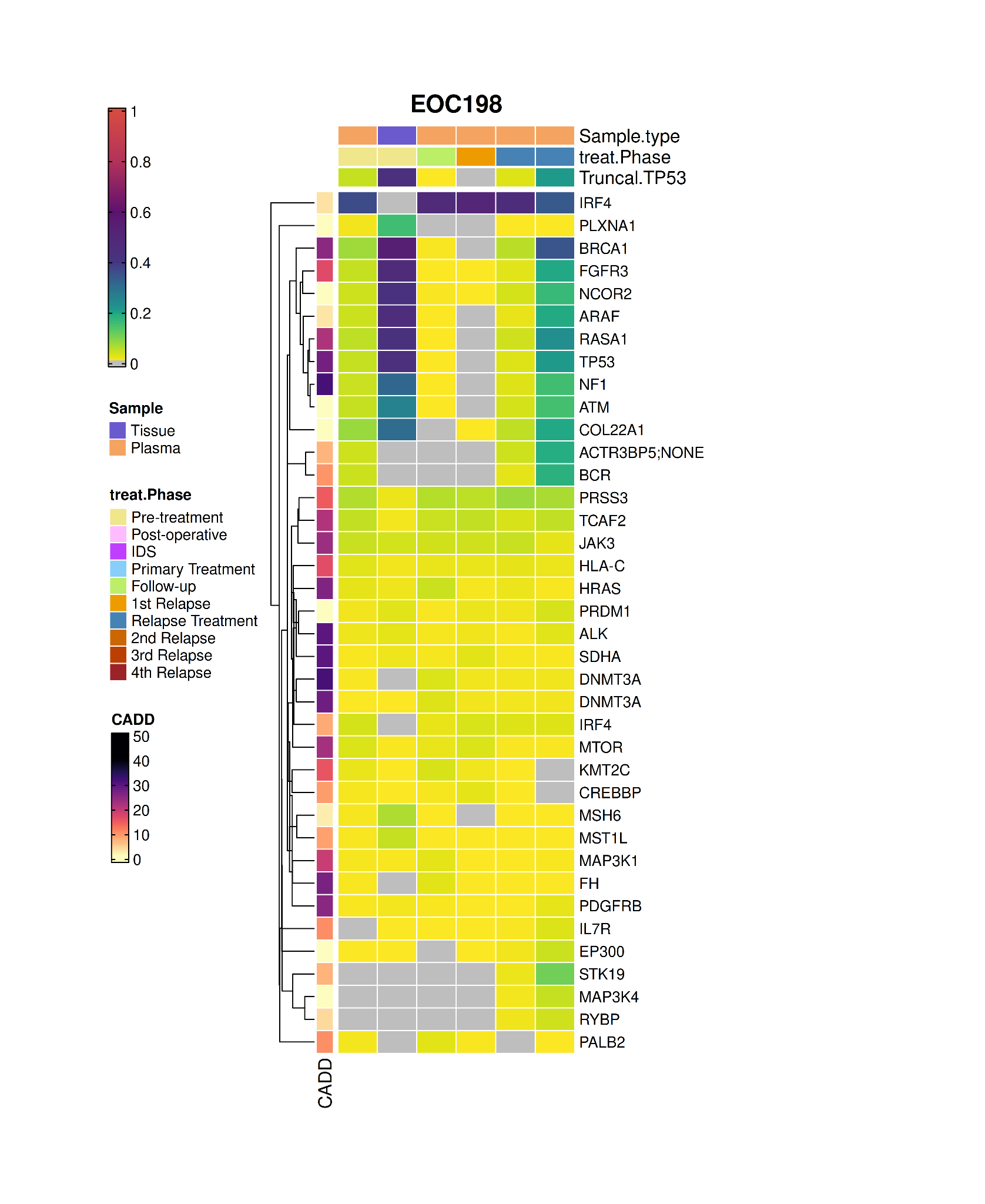 |
| 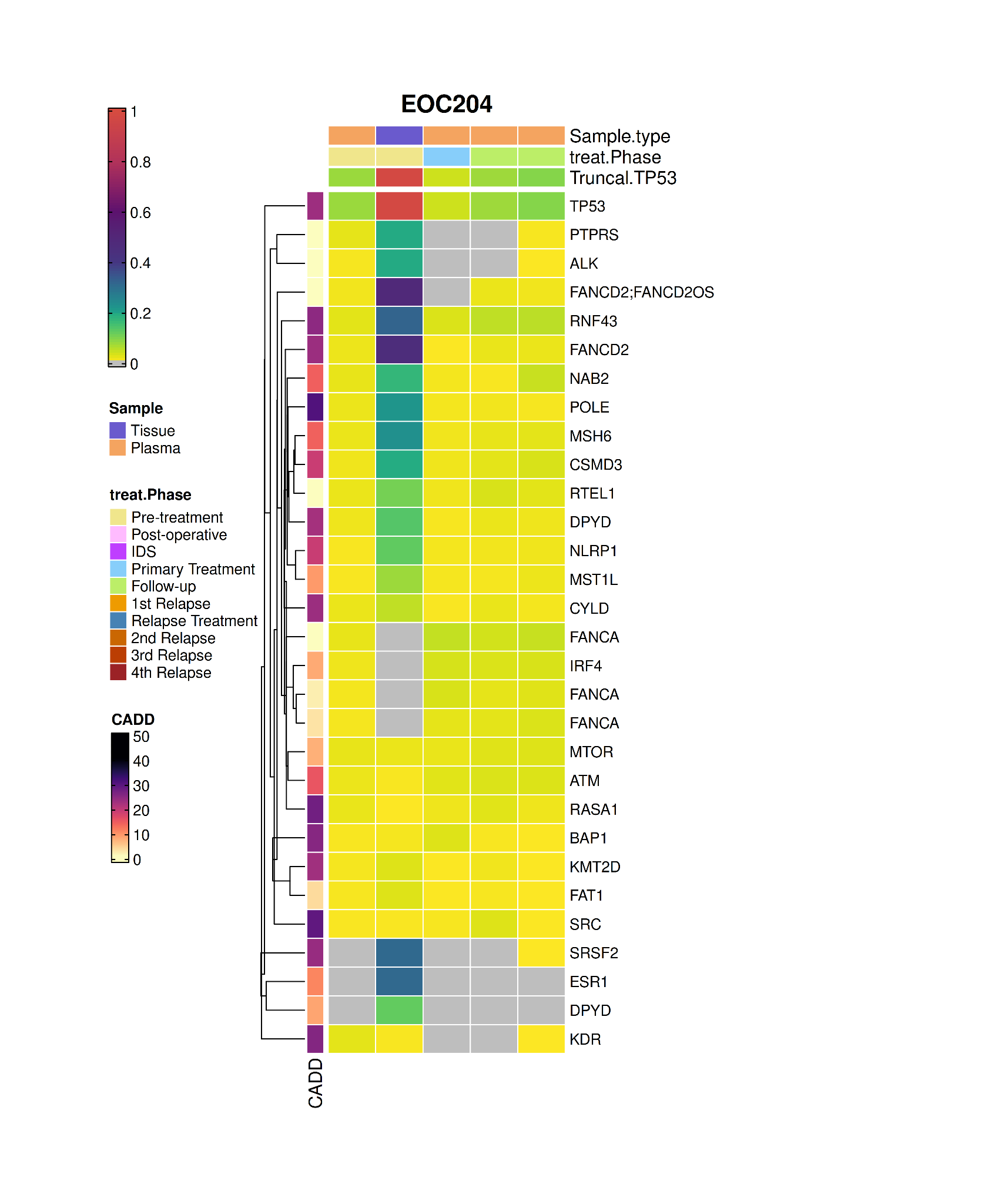 | 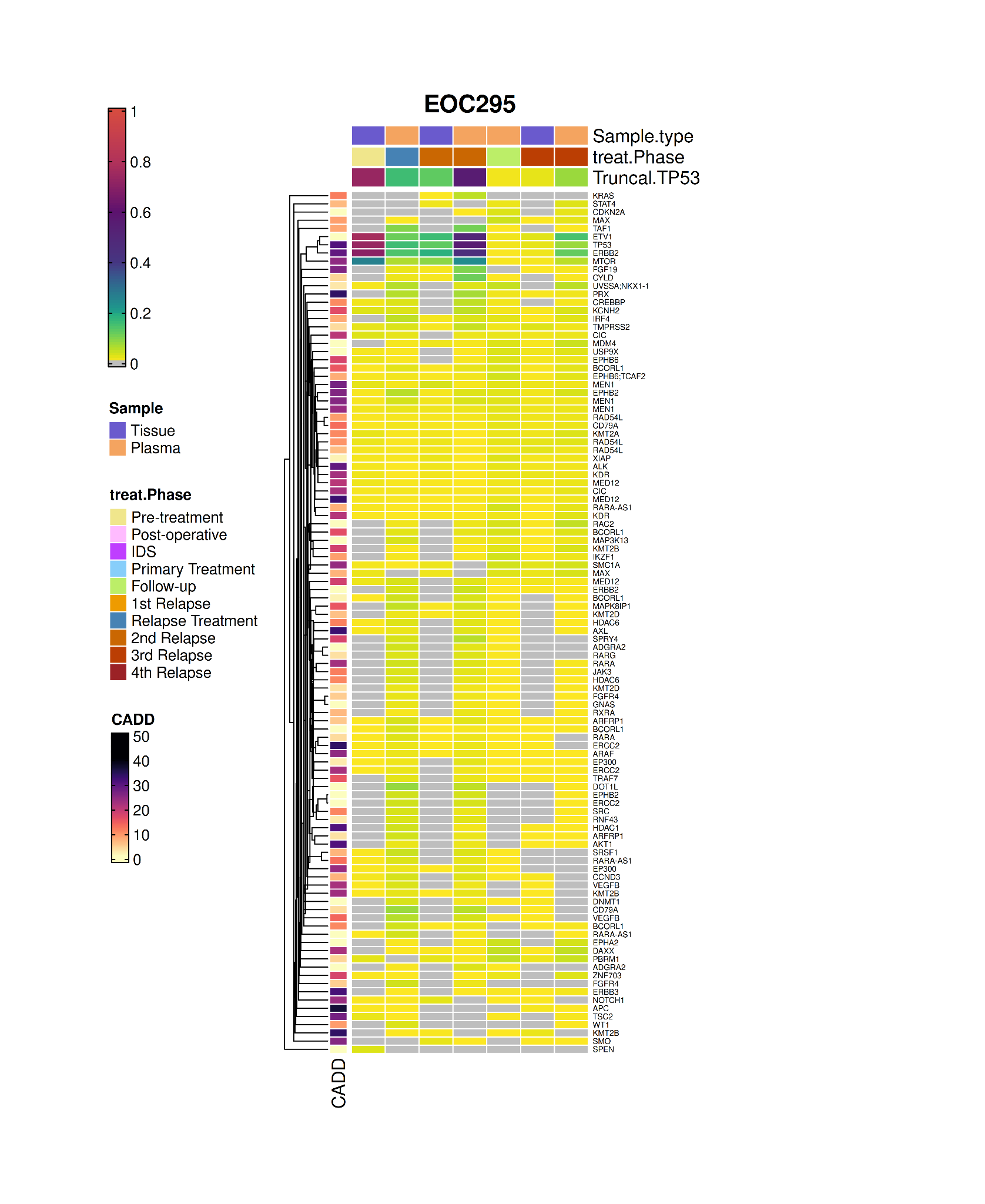 |
| 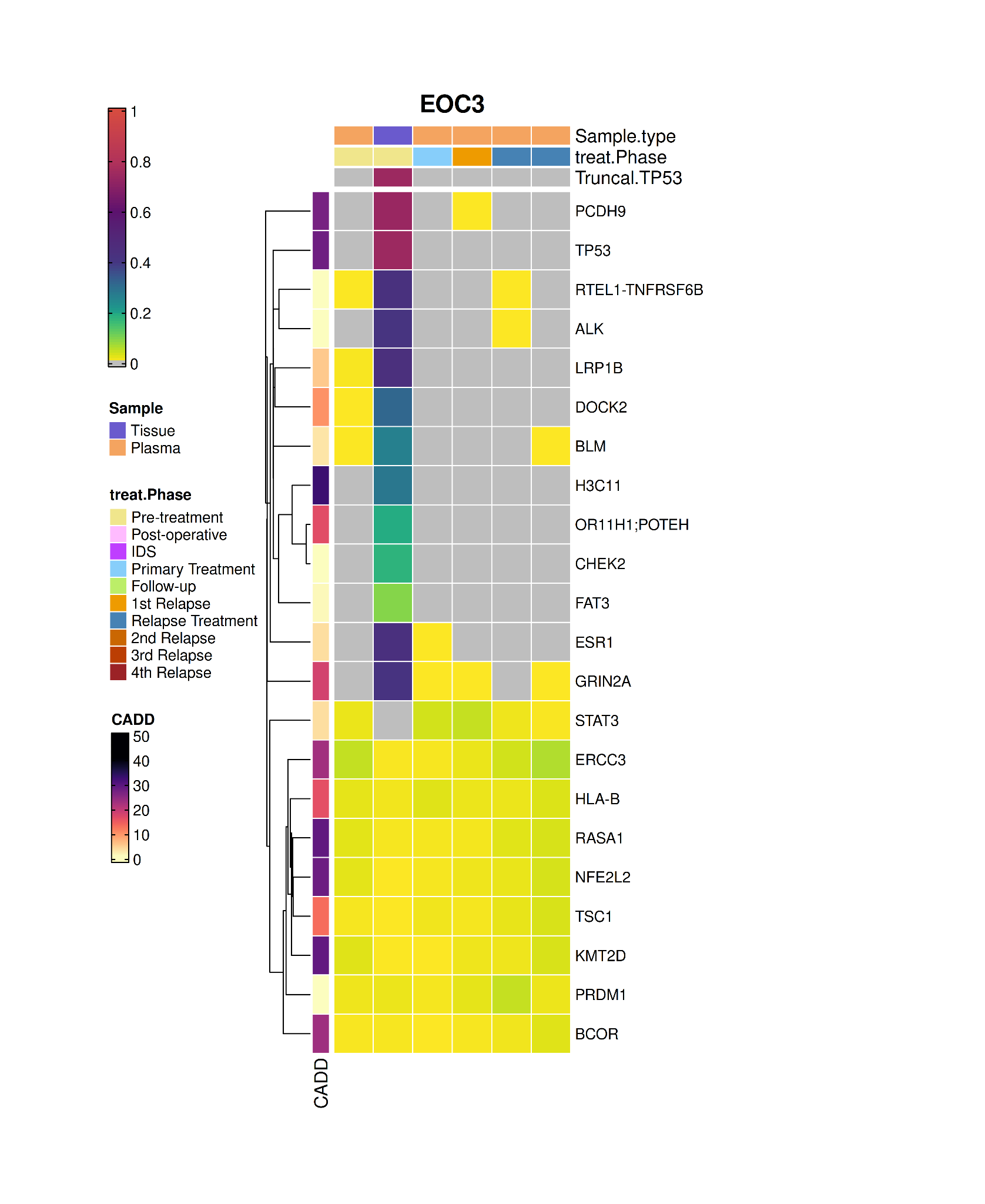 | 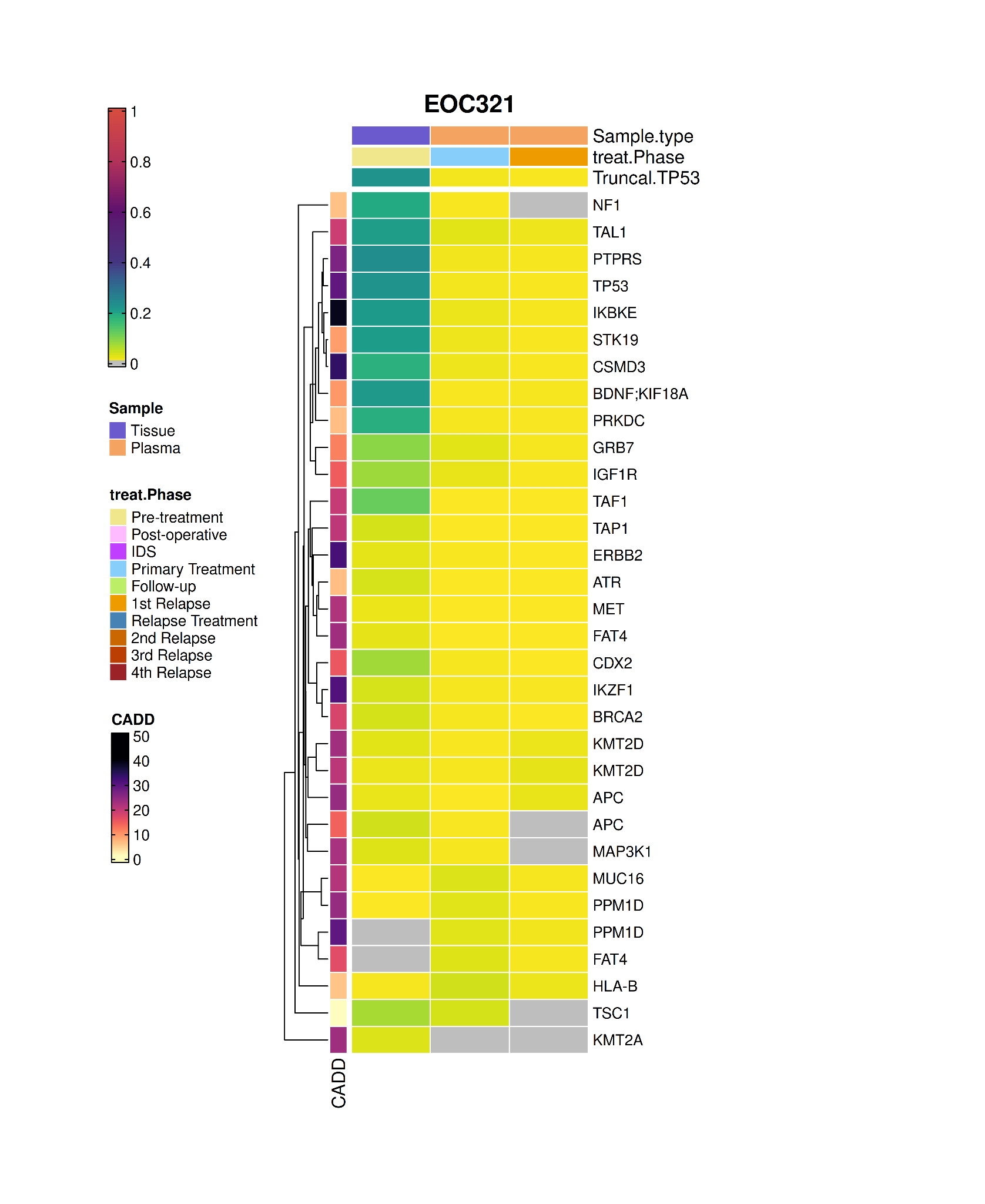 |
| 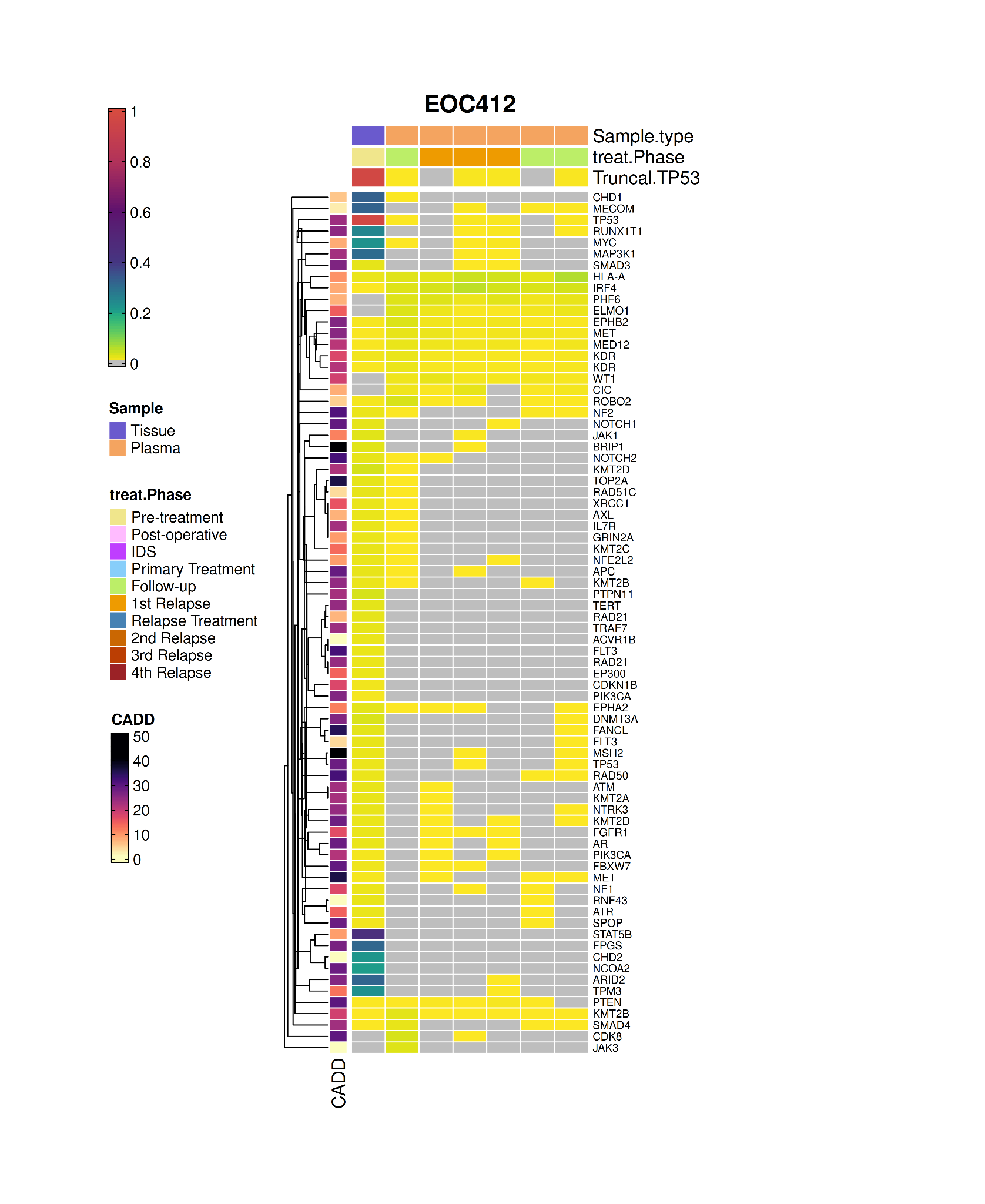 | 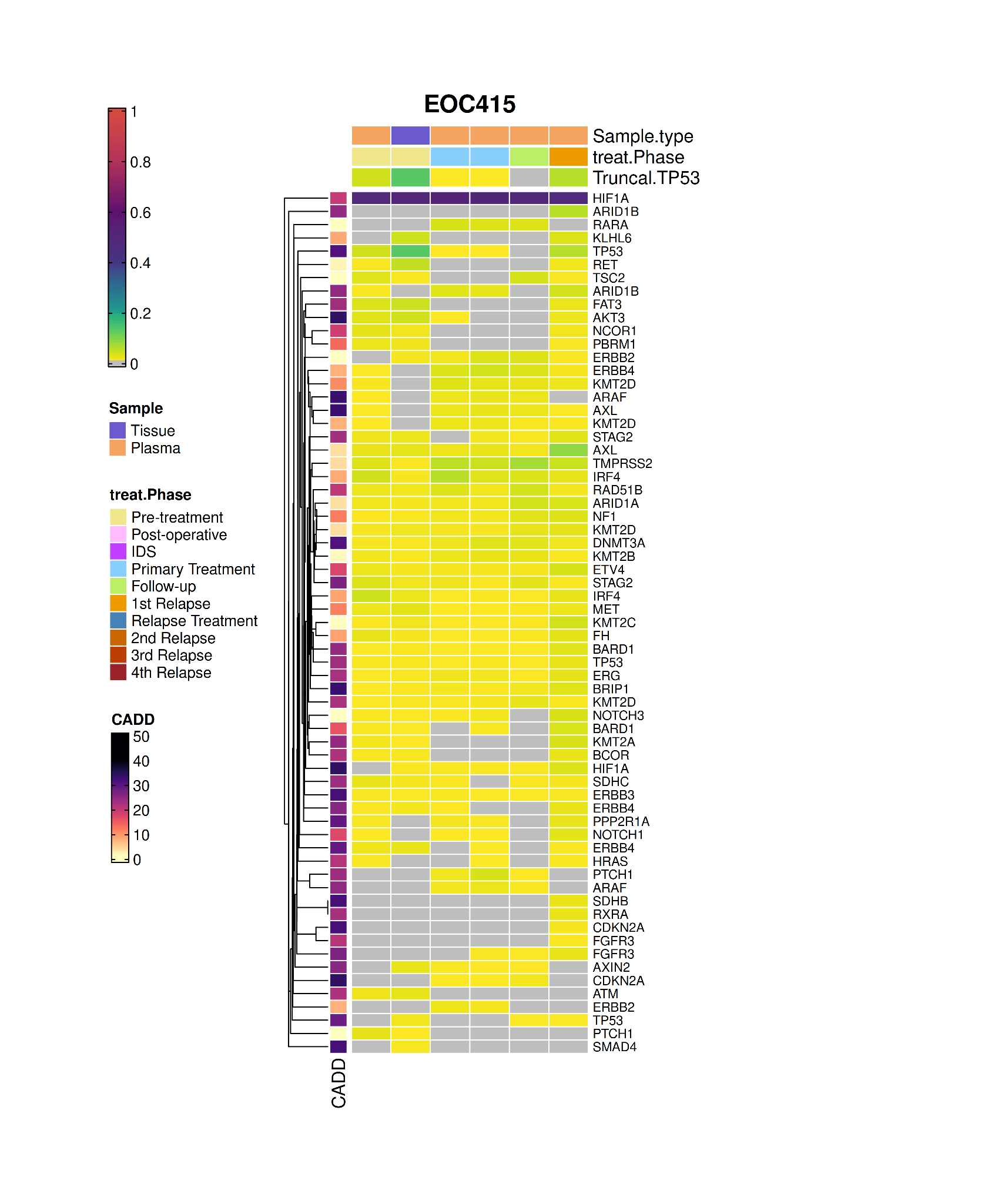 |
| 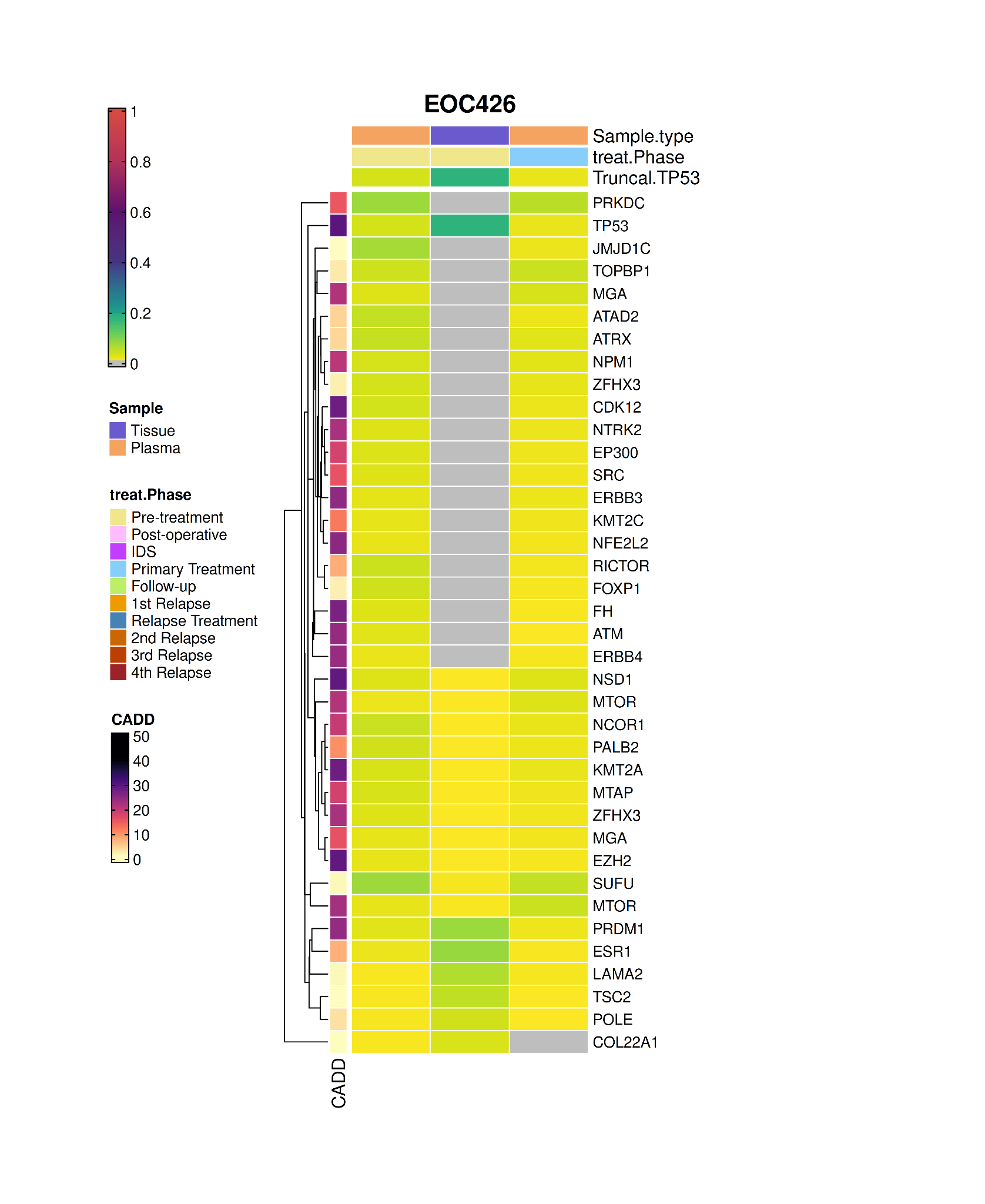 | 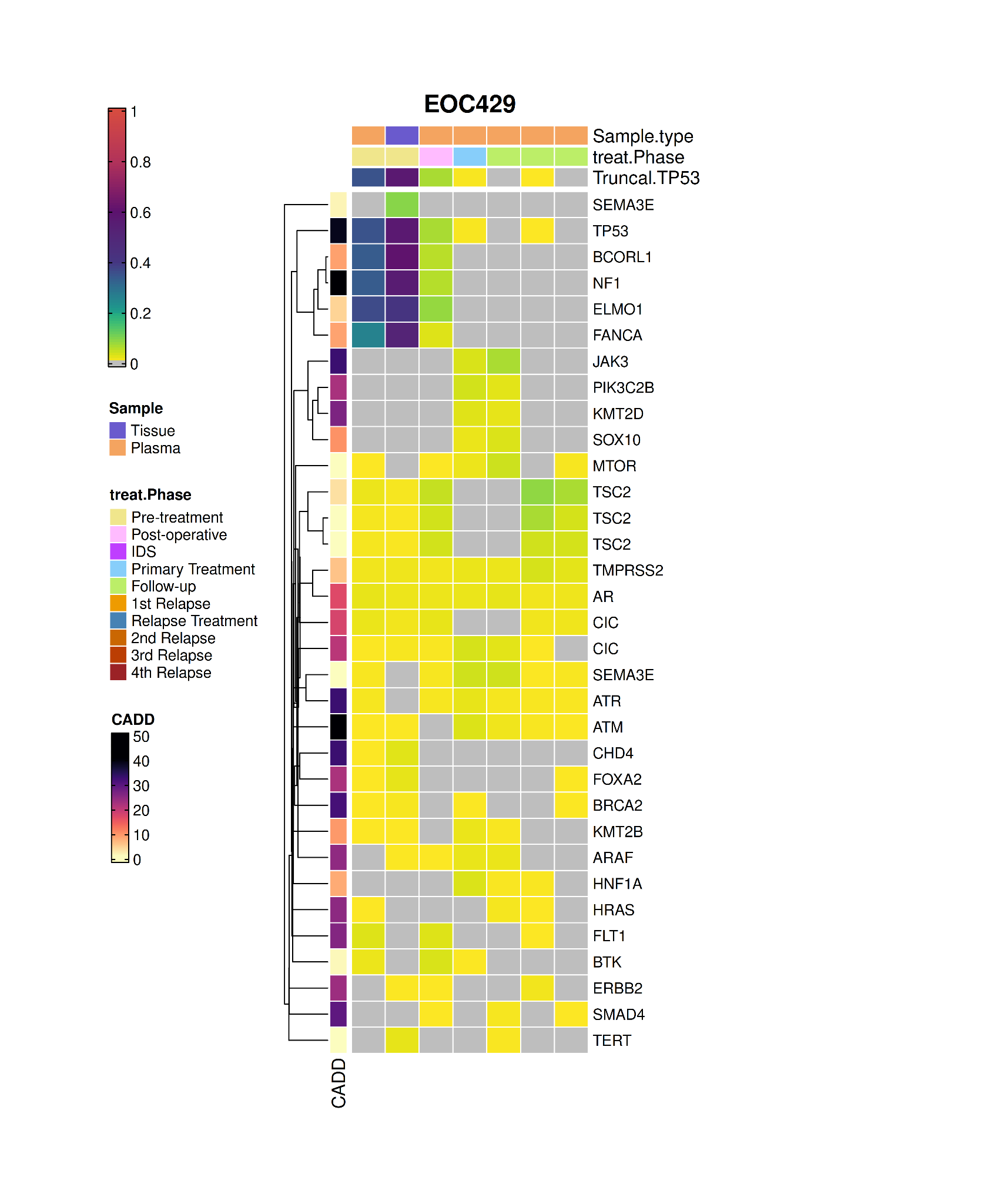 |
| 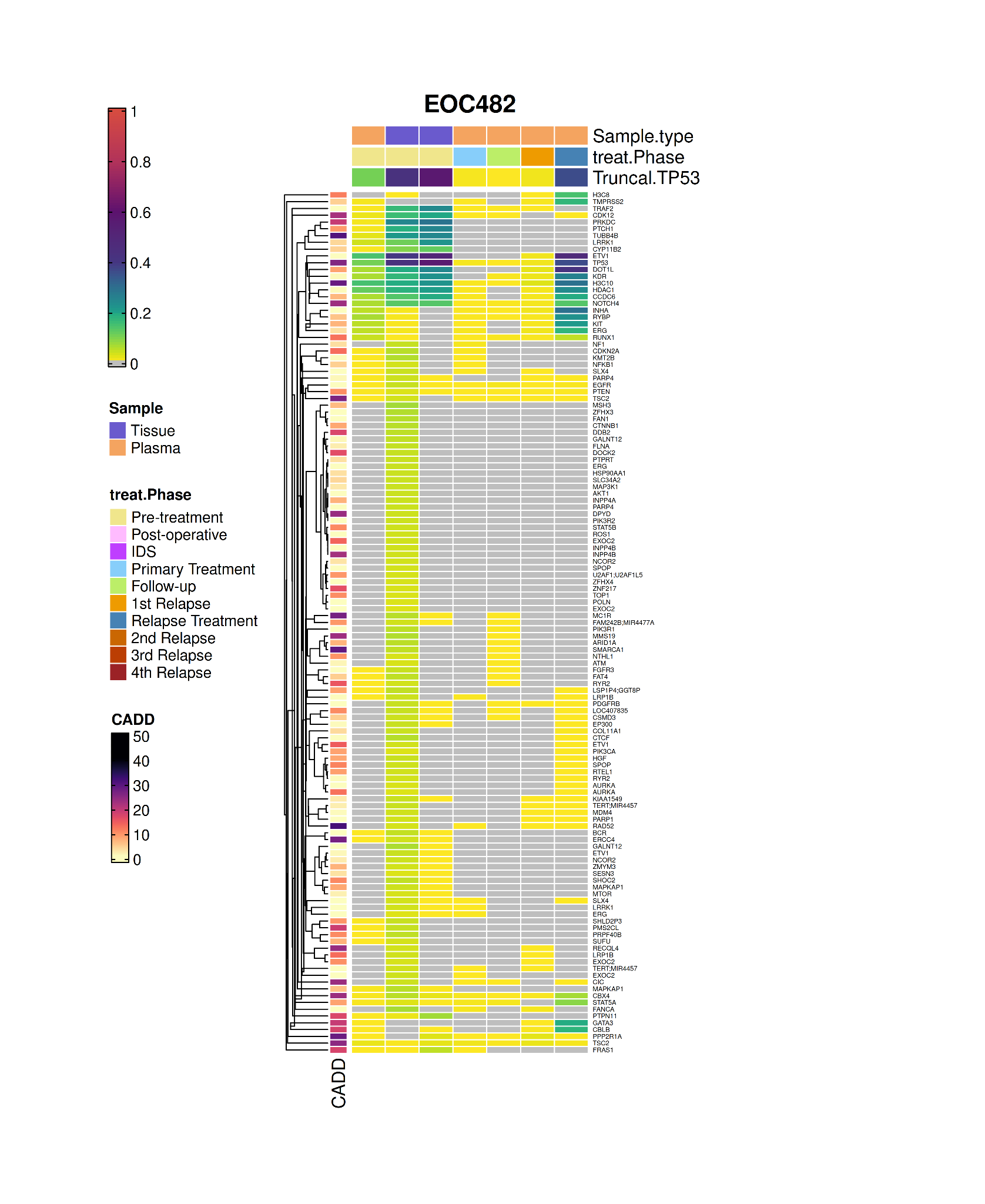 | 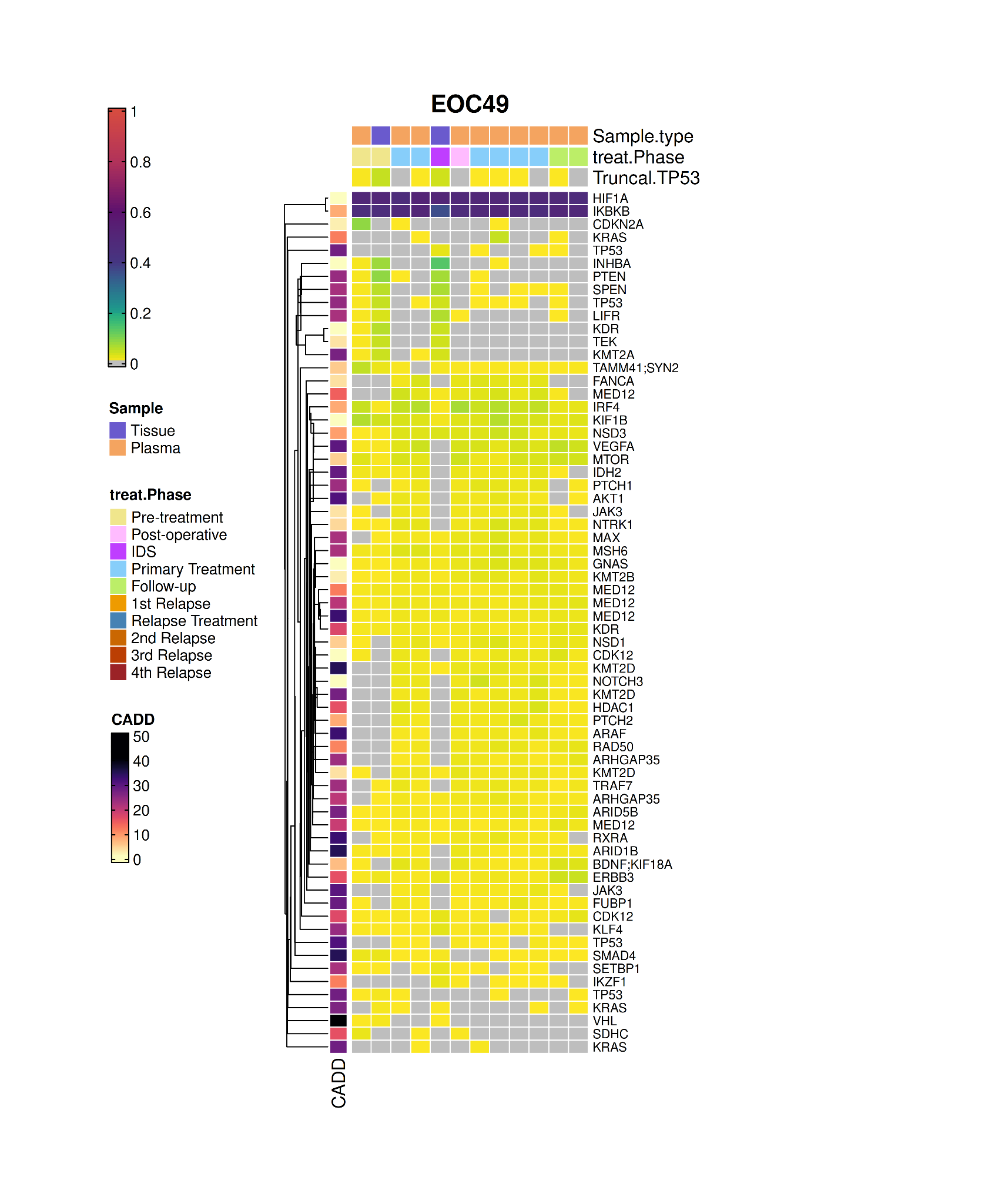 |
| 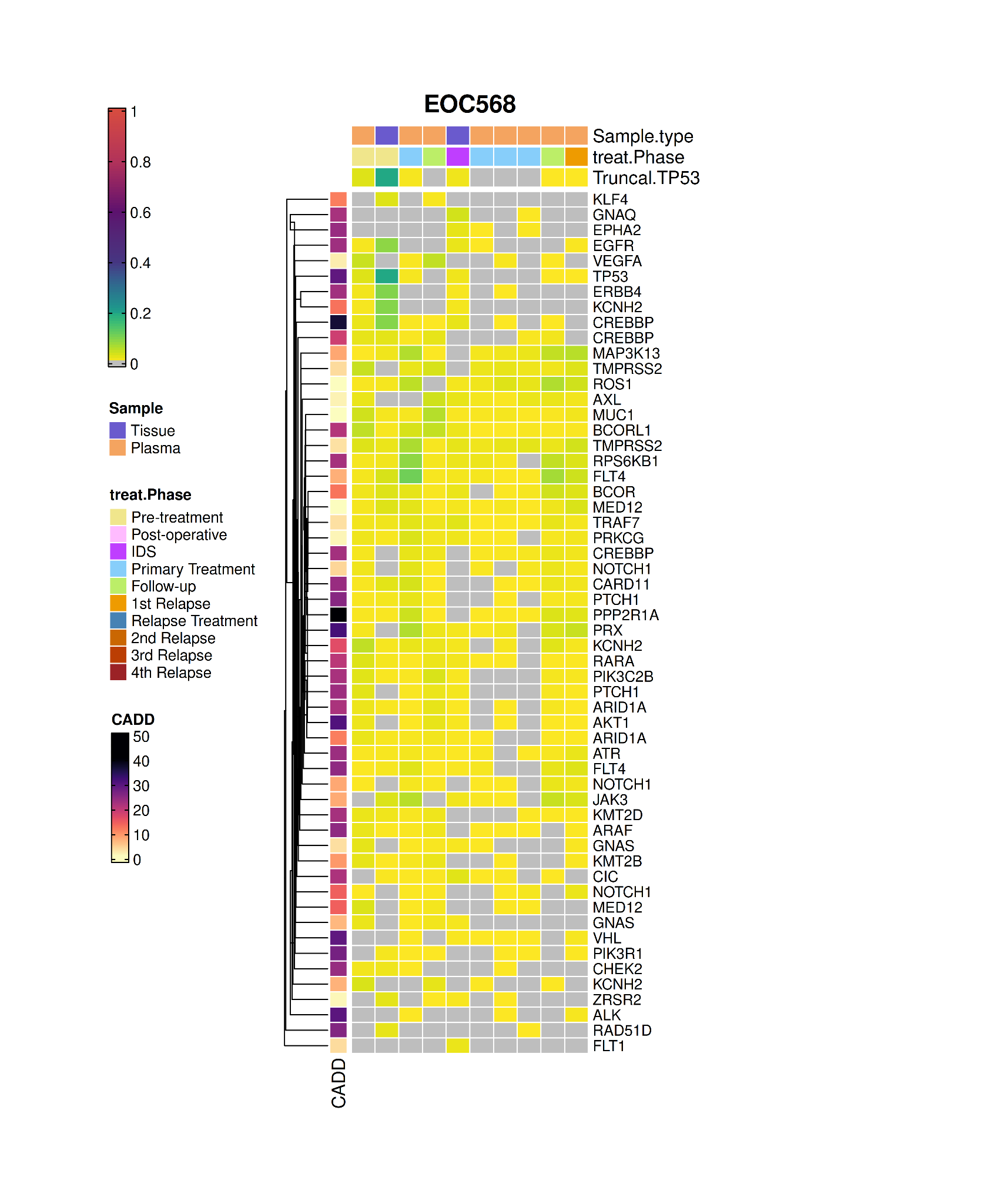 | 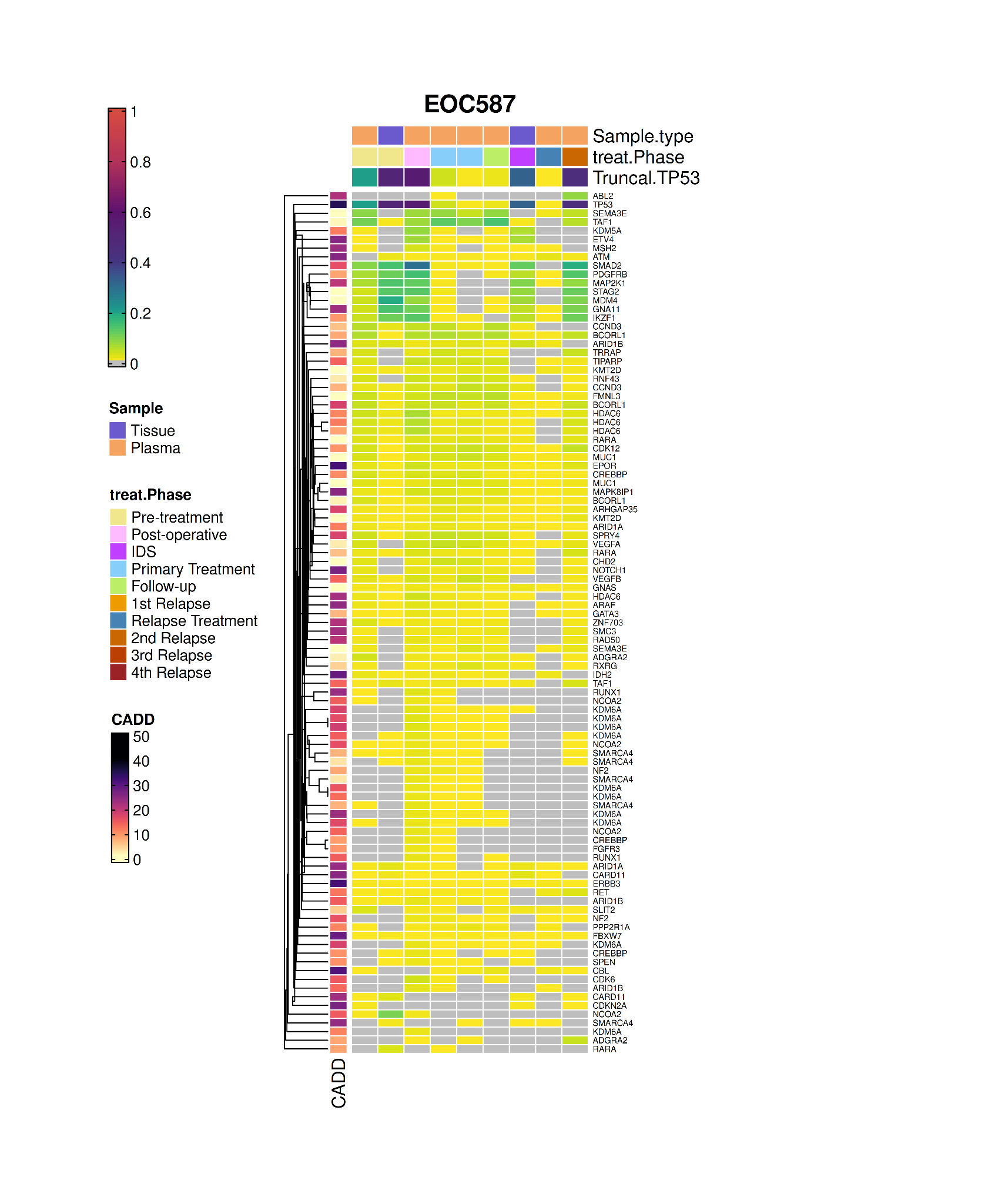 |
| 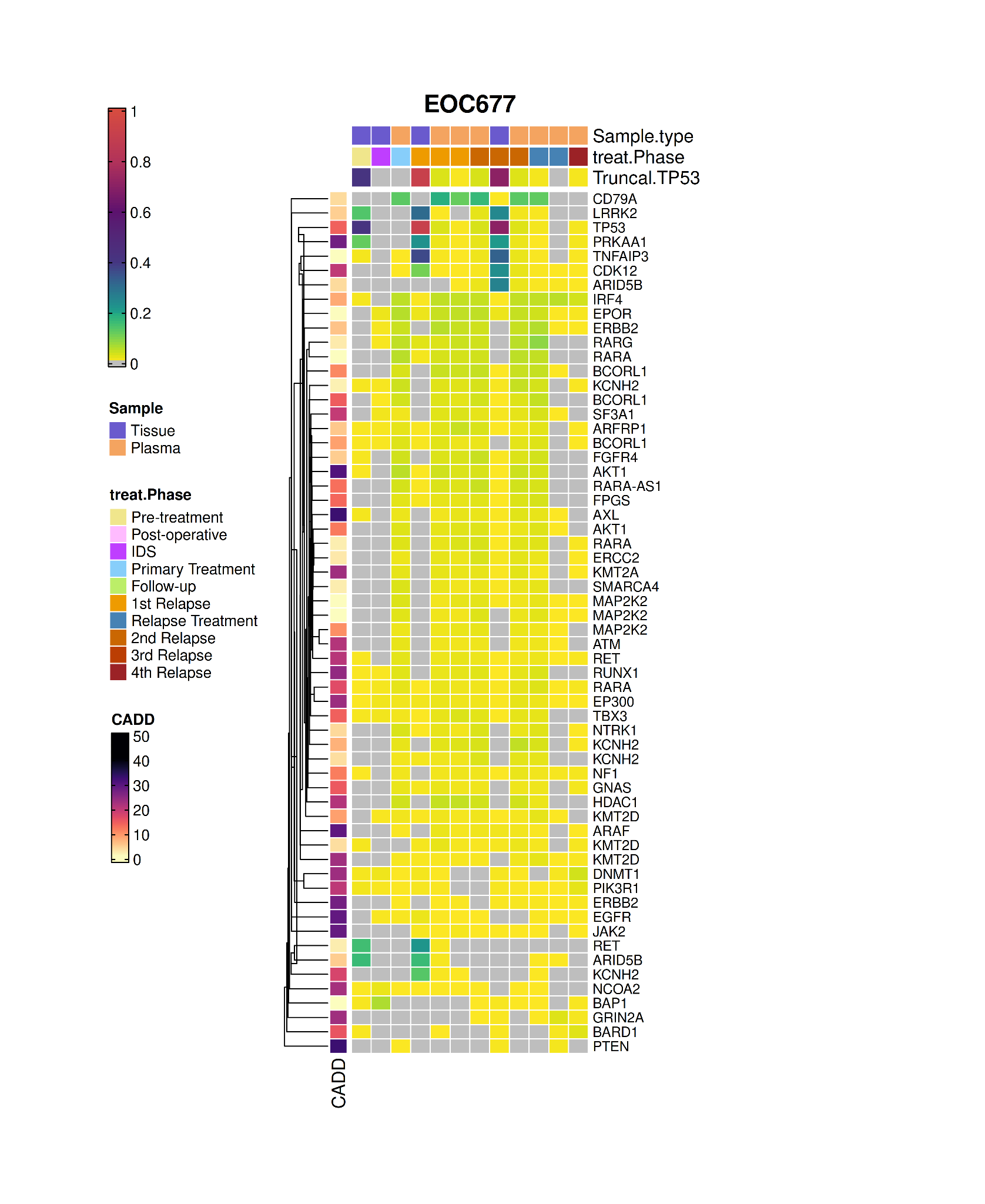 | 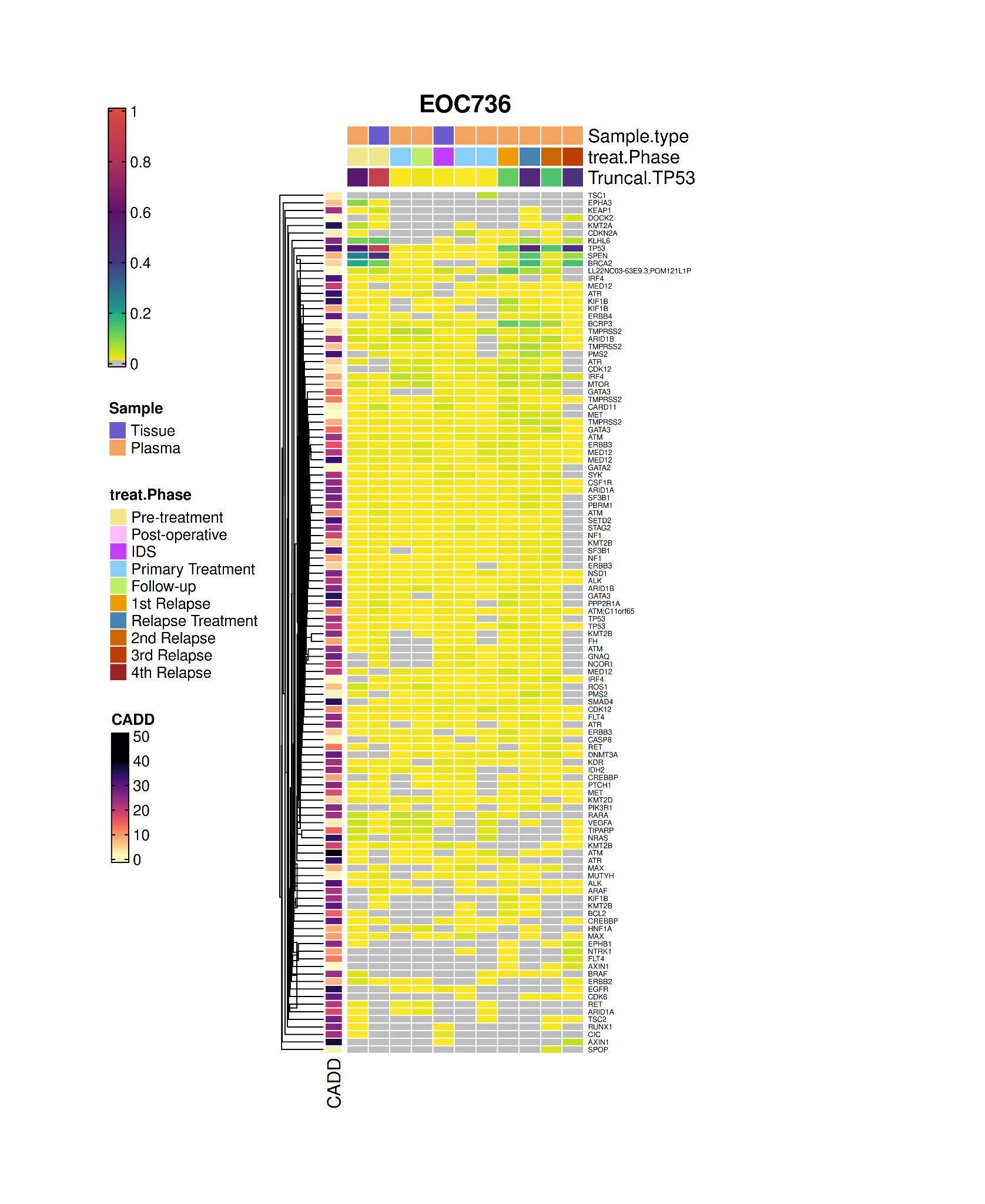 |
| 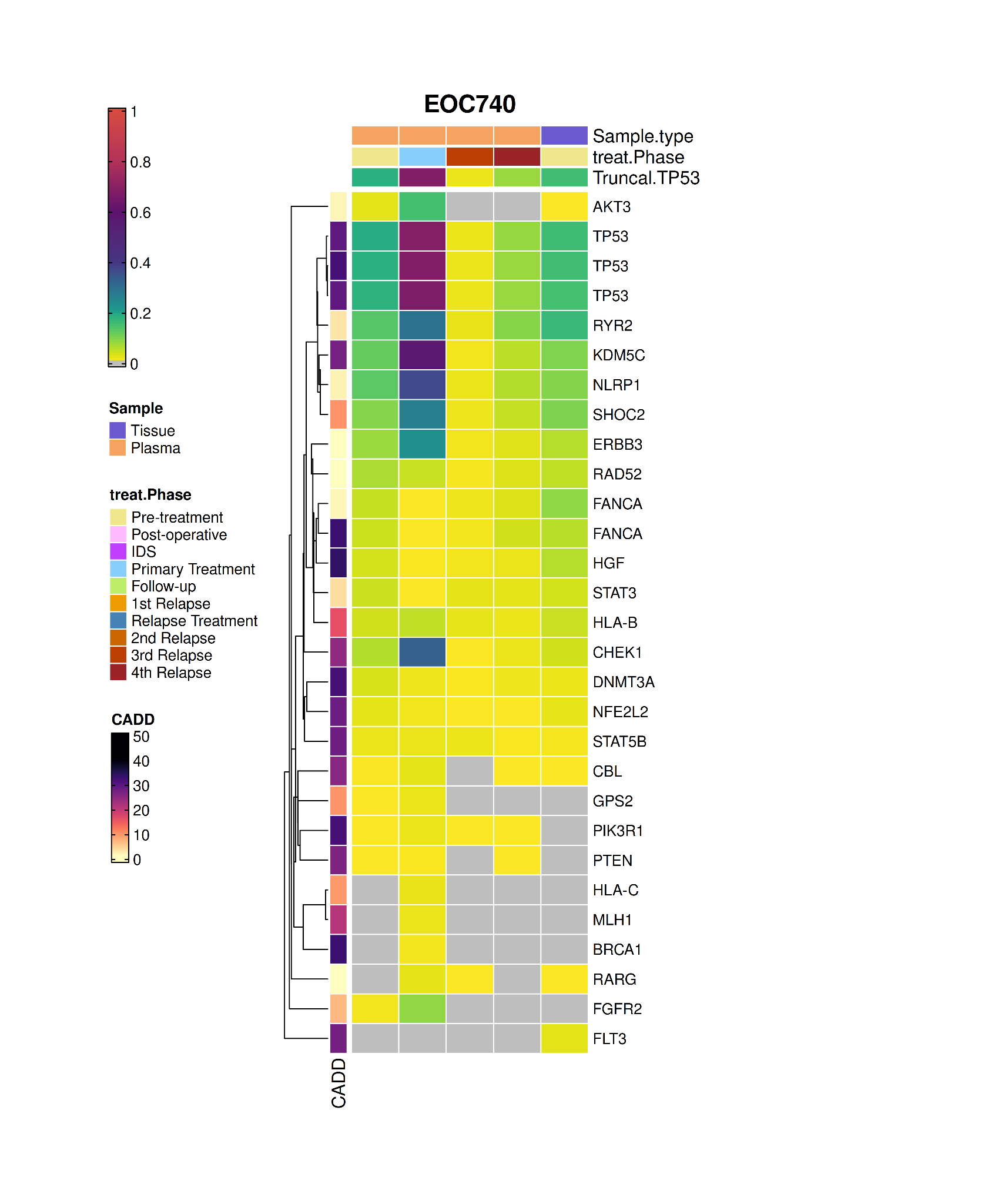 | 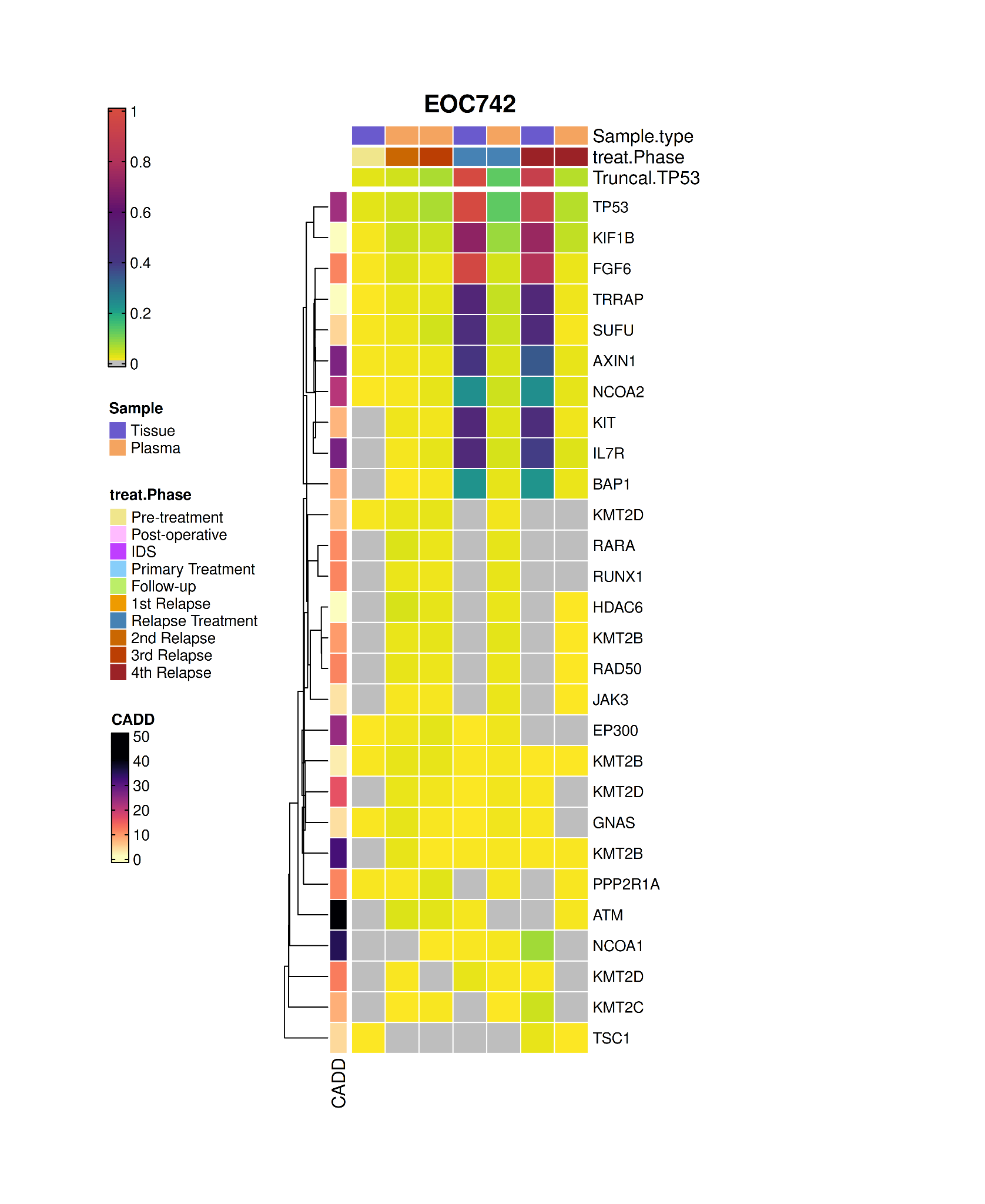 |
|  |  |
| 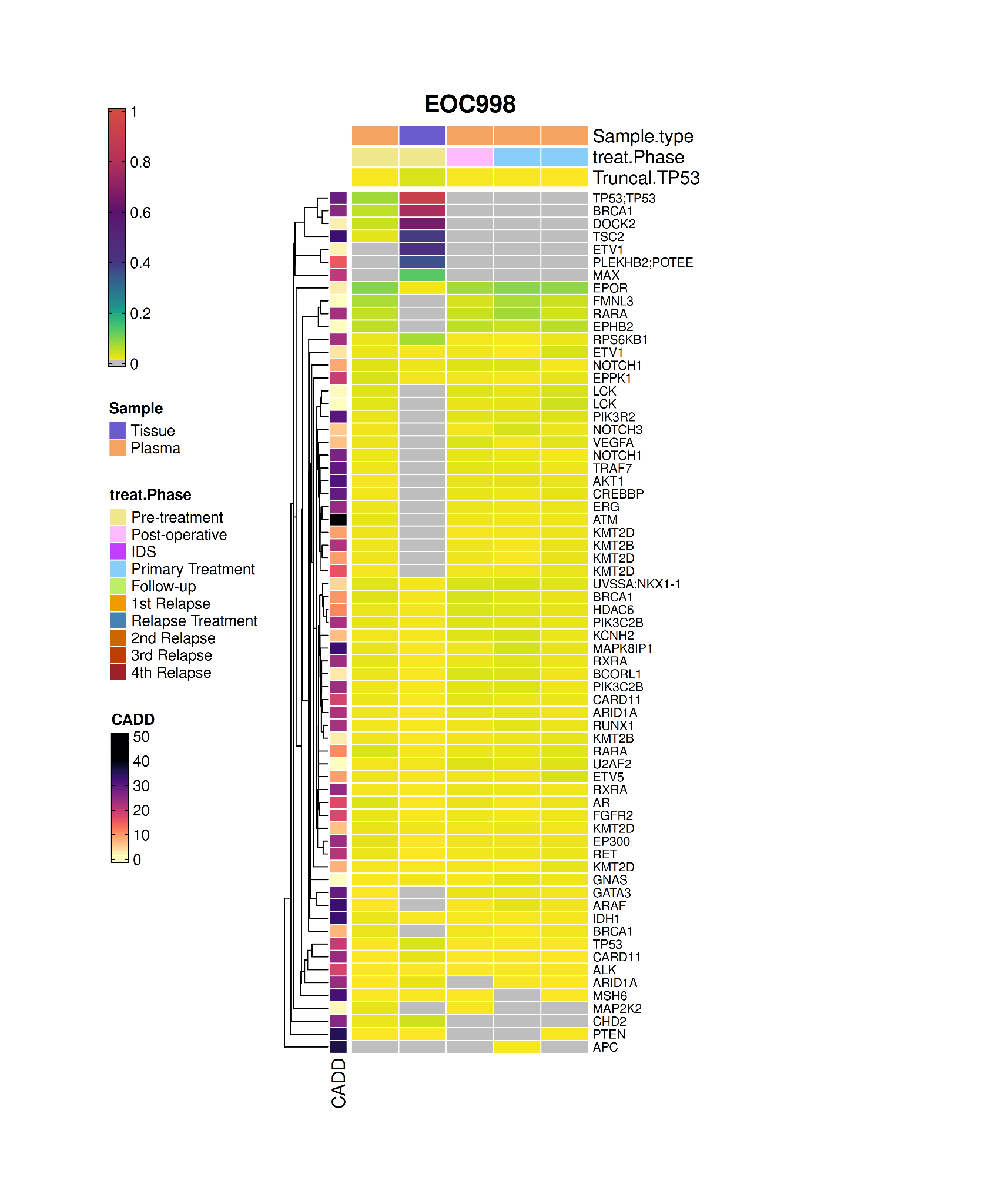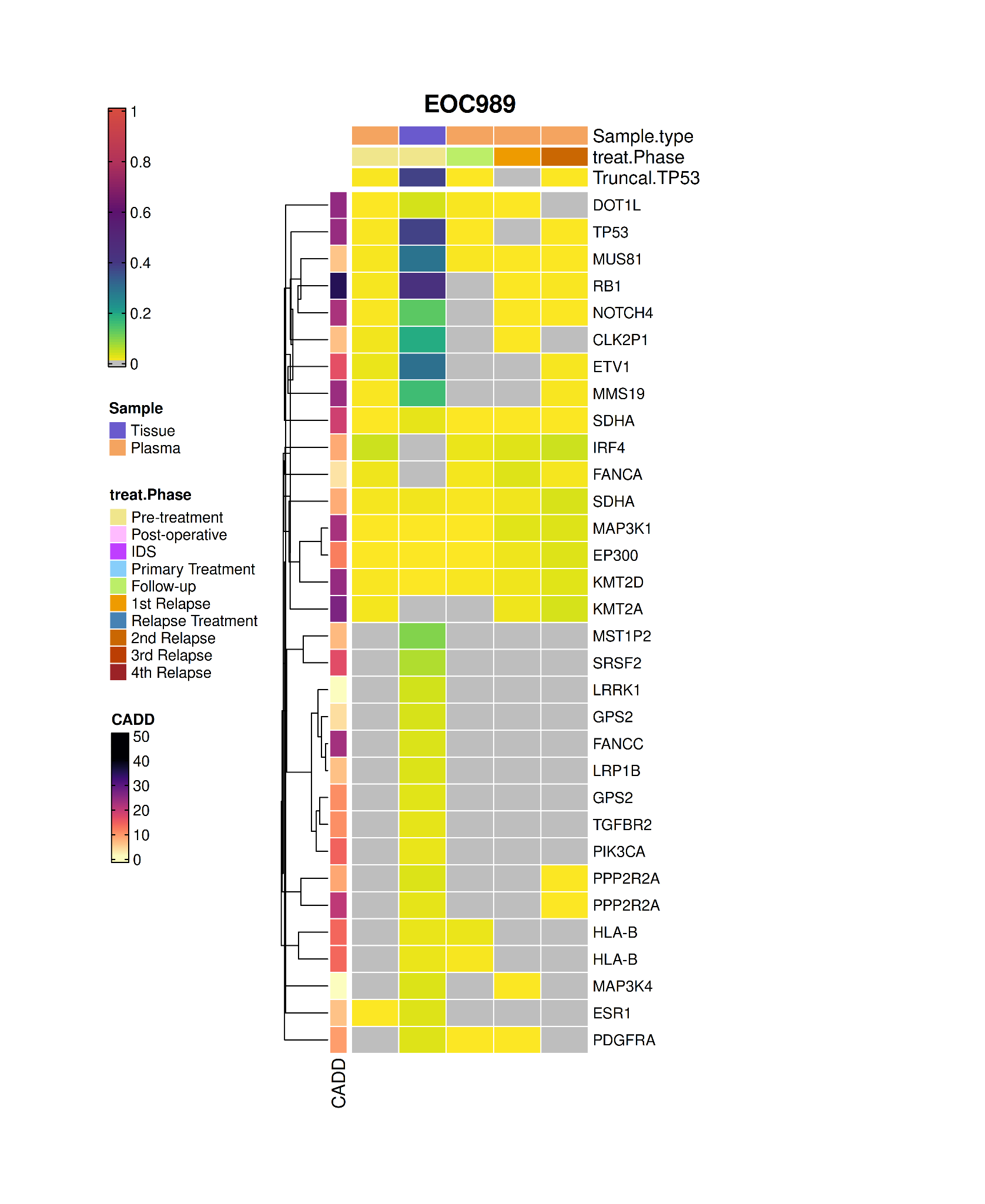 | 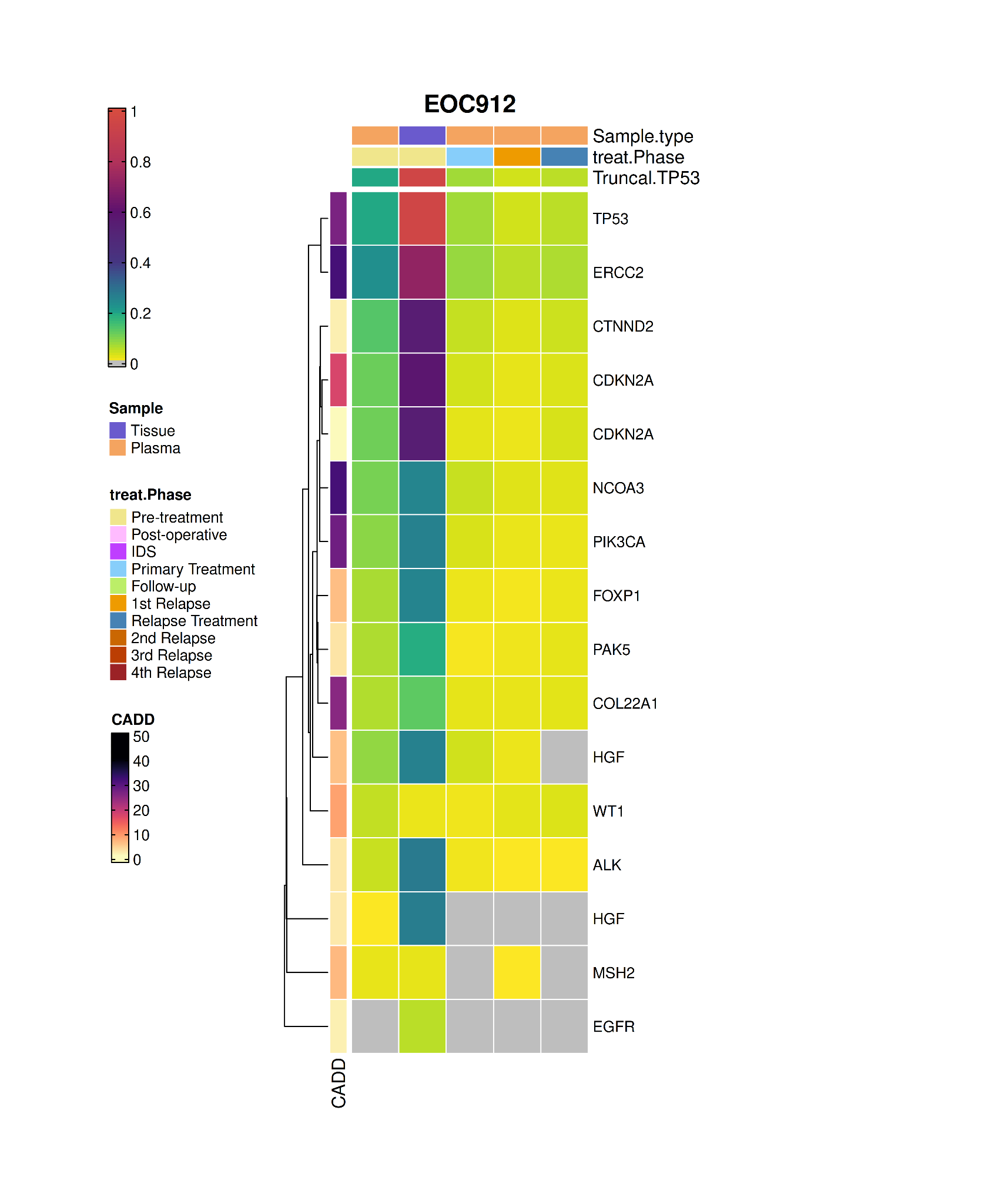 |

**Additional tables**

**Table S1** List of the two (Gene set1 and Gene set2) protein coding gene sets used in the DNA sequencing step.

*Separate file*

**Table S2**

**Clinical parameters and timing of longitudinal plasma samples per patient. ***follow up time, patient is alive

*Separate file*

**Supplementary references**

[1] Andrews, S., ‘FastQC:  A Quality Control Tool for High Throughput Sequence Data [Online]. Available online at: http://www.bioinformatics.babraham.ac.uk/projects/fastqc/’.

[2] A. M. Bolger, M. Lohse, and B. Usadel, ‘Trimmomatic: a flexible trimmer for Illumina sequence data’, *Bioinformatics*, vol. 30, no. 15, pp. 2114–2120, Aug. 2014, doi: 10.1093/bioinformatics/btu170.

[3] H. Li and R. Durbin, ‘Fast and accurate short read alignment with Burrows–Wheeler transform’, *Bioinformatics*, vol. 25, no. 14, pp. 1754–1760, Jul. 2009, doi: 10.1093/bioinformatics/btp324.

[4] ‘Picard tool. http://broadinstitute.github.io/picard/’. Broad Institute, 2018.

[5] A. McKenna *et al.*, ‘The Genome Analysis Toolkit: A MapReduce framework for analyzing next-generation DNA sequencing data’, *Genome Res.*, vol. 20, no. 9, pp. 1297–1303, Sep. 2010, doi: 10.1101/gr.107524.110.

[6] ‘SAGE: New algorithm for analysis of tumor DNA reveals mutations previously not found’. Hartwig Medical Foundation, Jul. 23, 2020. [Online]. Available: https://www.hartwigmedicalfoundation.nl/en/sage-nieuw-algoritme-voor-analyse-van-tumor-dna-onthult-mutaties-die-eerder-niet-waren-gevonden/

[7] N. A. O’Leary *et al.*, ‘Reference sequence (RefSeq) database at NCBI: current status, taxonomic expansion, and functional annotation’, *Nucleic Acids Res.*, vol. 44, no. D1, pp. D733–D745, Jan. 2016, doi: 10.1093/nar/gkv1189.

[8] J. Morales *et al.*, ‘A joint NCBI and EMBL-EBI transcript set for clinical genomics and research’, *Nature*, vol. 604, no. 7905, pp. 310–315, Apr. 2022, doi: 10.1038/s41586-022-04558-8.

[9] J. Navarro Gonzalez *et al.*, ‘The UCSC Genome Browser database: 2021 update’, *Nucleic Acids Res.*, vol. 49, no. D1, pp. D1046–D1057, Jan. 2021, doi: 10.1093/nar/gkaa1070.

[10] J. G. Tate *et al.*, ‘COSMIC: the Catalogue Of Somatic Mutations In Cancer’, *Nucleic Acids Res.*, vol. 47, no. D1, pp. D941–D947, Jan. 2019, doi: 10.1093/nar/gky1015.

[11] M. J. Landrum *et al.*, ‘ClinVar: improving access to variant interpretations and supporting evidence’, *Nucleic Acids Res.*, vol. 46, no. D1, pp. D1062–D1067, Jan. 2018, doi: 10.1093/nar/gkx1153.

[12] X. Liu, C. Li, C. Mou, Y. Dong, and Y. Tu, ‘dbNSFP v4: a comprehensive database of transcript-specific functional predictions and annotations for human nonsynonymous and splice-site SNVs’, *Genome Med.*, vol. 12, no. 1, p. 103, Dec. 2020, doi: 10.1186/s13073-020-00803-9.

[13] The 1000 Genomes Project Consortium *et al.*, ‘A global reference for human genetic variation’, *Nature*, vol. 526, no. 7571, pp. 68–74, Oct. 2015, doi: 10.1038/nature15393.

[14] K. J. Karczewski *et al.*, ‘The mutational constraint spectrum quantified from variation in 141,456 humans’, *Nature*, vol. 581, no. 7809, pp. 434–443, May 2020, doi: 10.1038/s41586-020-2308-7.

[15] P. Rentzsch, D. Witten, G. M. Cooper, J. Shendure, and M. Kircher, ‘CADD: predicting the deleteriousness of variants throughout the human genome’, *Nucleic Acids Res.*, vol. 47, no. D1, pp. D886–D894, Jan. 2019, doi: 10.1093/nar/gky1016.

[16] Smit, AFA, Hubley, and R & Green, ‘P. RepeatMasker Open-4.0. 2013-2015 <http://www.repeatmasker.org>.’

[17] A. Morgulis, E. M. Gertz, A. A. Schäffer, and R. Agarwala, ‘WindowMasker: window-based masker for sequenced genomes’, *Bioinformatics*, vol. 22, no. 2, pp. 134–141, Jan. 2006, doi: 10.1093/bioinformatics/bti774.
